# Functional bipartite invariance in mouse primary visual cortex receptive fields

**DOI:** 10.1038/s41593-026-02213-3

**Published:** 2026-02-25

**Authors:** Zhiwei Ding, Dat Tran, Kayla Ponder, Zhuokun Ding, Rachel Froebe, Lydia Ntanavara, Paul G. Fahey, Erick Cobos, Luca Baroni, Maria Diamantaki, Eric Y. Wang, Andersen Chang, Stelios Papadopoulos, Jiakun Fu, Taliah Muhammad, Christos Papadopoulos, Santiago A. Cadena, Alexandros Evangelou, Konstantin Willeke, Fabio Anselmi, Sophia Sanborn, Jan Antolik, Emmanouil Froudarakis, Saumil Patel, Edgar Y. Walker, Jacob Reimer, Fabian H. Sinz, Alexander S. Ecker, Katrin Franke, Xaq Pitkow, Andreas S. Tolias

**Affiliations:** 1 Center for Neuroscience and Artificial Intelligence, Baylor College of Medicine, Houston, TX USA; 2 Department of Neuroscience, Baylor College of Medicine, Houston, TX USA; 3 Department of Ophthalmology, Byers Eye Institute, Stanford University School of Medicine, Stanford, CA USA; 4 Stanford Bio-X, Stanford University, Stanford, CA USA; 5 Wu Tsai Neurosciences Institute, Stanford University, Stanford, CA USA; 6 Faculty of Mathematics and Physics, Charles University, Prague, Czech Republic; 7 Institute of Molecular Biology & Biotechnology, Foundation of Research & Technology - Hellas, Heraklion, Greece; 8 School of Medicine, University of Crete, Heraklion, Greece; 9 The Salk Institute for Biological Studies, La Jolla, CA USA; 10 Institute of Computer Science and Campus Institute Data Science, University of Göttingen, Göttingen, Germany; 11 Institute for Bioinformatics and Medical Informatics, University of Tübingen, Tübingen, Germany; 12 Department of Mathematics, Informatics and Geoscience, University of Trieste, Trieste, Italy; 13 Department of Physiology and Biophysics, University of Washington, Seattle, WA USA; 14 Computational Neuroscience Center, University of Washington, Seattle, WA USA; 15 Department of Electrical and Computer Engineering, Rice University, Houston, TX USA; 16 Max Planck Institute for Dynamics and Self-Organization, Göttingen, Germany; 17 Institute for Ophthalmic Research, University of Tübingen, Tübingen, Germany; 18 Department of Computer Science, Rice University, Houston, TX USA; 19 Neuroscience Institute, Carnegie Mellon University, Pittsburgh, PA USA; 20 Department of Machine Learning, Carnegie Mellon University, Pittsburgh, PA USA; 21 Department of Electrical Engineering, Stanford University, Stanford, CA USA

**Keywords:** Neural encoding, Object vision, Pattern vision

## Abstract

Sensory systems support generalization by representing features that persist under input variation; however, identifying the neuronal basis of these invariances remains difficult due to high-dimensional and nonlinear neural computations. Here we leverage the inception loop paradigm, iterating between large-scale recordings, predictive models and in silico experiments with in vivo verification, to characterize neuronal invariances in mouse primary visual cortex (V1). We synthesize varied exciting inputs (VEIs), dissimilar images that drive target neurons. These VEIs revealed a new bipartite invariance: one subfield encodes a shift-tolerant high-frequency texture and the other encodes a fixed low-frequency pattern. This division aligns with object boundaries defined by spatial frequency differences in highly activating images, suggesting a contribution to segmentation. Analysis of the MICrONS dataset revealed a hierarchy of excitatory neurons in mouse V1 layers 2/3: postsynaptic neurons exhibited greater invariance than their presynaptic inputs, while neurons with lower invariance formed more connections. Together, these results provide insights and scalable methodology for mapping neuronal invariances.

## Abstract (web summary)

This study uses the inception loop framework to map neuronal invariances in mouse V1, revealing a bipartite receptive-field organization linked to segmentation and a synaptic-level hierarchy of increasing invariance supported by the MICrONS dataset.

## Main

A central challenge of visual perception is to infer latent features despite fluctuations in raw sensory inputs. Recognizing a familiar face in a crowd requires extracting relevant features across changes in distance, three-dimensional (3D) pose, scale and illumination. While these variations are considered ‘nuisance’ variables, the brain must represent them because they are crucial for other tasks, such as navigating the crowd to approach the familiar face.

To understand how brains effectively disentangle high-dimensional sensory inputs and robustly extract latent variables^1^, we must identify the features to which neurons exhibit selectivity (features that evoke maximal responses) and invariance (feature variations that preserve a high response magnitude). Identifying neuronal invariances is extremely challenging because of the enormous search space of visual stimuli, the nonlinear information processing in the brain and the limited experimental time. Consequently, previous studies have been limited to parametric stimuli (for example, gratings) or semantic categories (for example, objects and faces)^2–5^, chosen based on strong assumptions about the invariance structure. The classic example is Hubel and Wiesel’s complex cells in the primary visual cortex (V1)^6^, which are tuned to gratings of a preferred orientation but invariant to spatial phase, in contrast to simple cells, which are selective to both orientation and spatial phase. Beyond such classical parametric invariances, however, we still lack a systematic, general framework for uncovering other forms of invariances.

Here, we take a data-driven, systematic approach to study neuronal invariances, leveraging the previously introduced ‘inception loop’ paradigm^7^. Using large-scale calcium imaging data, we trained a deep neural network model to accurately predict mouse V1 neuronal responses to arbitrary, new natural images. This model enables high-throughput in silico experiments, revealing neuronal response properties unattainable through traditional in vivo methods.

**Fig. 1:**
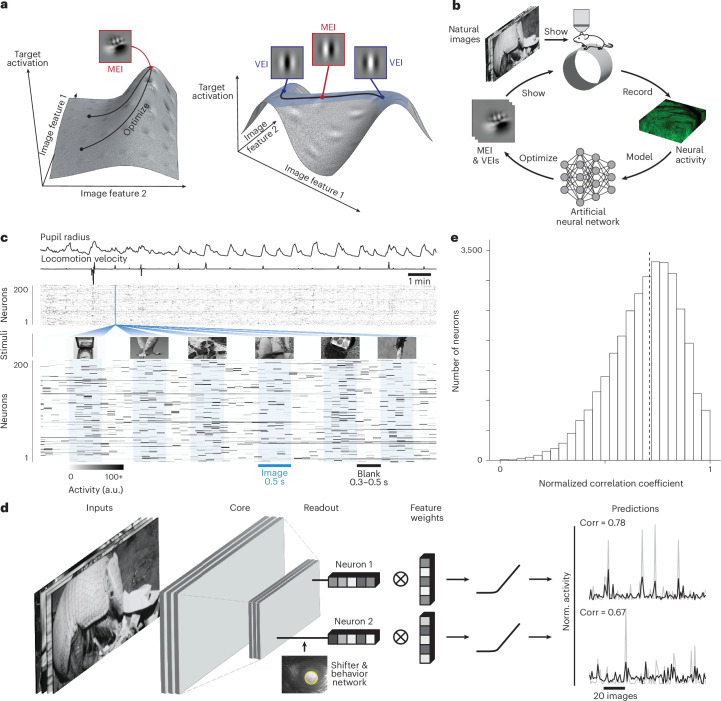
A deep neural network model accurately predicts mouse V1 responses to natural scenes. **a**, Schematic of the optimization of MEIs and VEIs. The vertical axes depict the activation of two model neurons as a function of two example image features. Left, neuron without obvious invariance; right, neuron with phase invariance to its optimal stimulus. Black curves illustrate optimization trajectories for MEI from different initializations (left) and for VEIs as perturbations starting from the MEI along the invariance ridge (right). **b**, Schematic of the inception loop paradigm. On day 1, we presented sequences of natural images and recorded in vivo neuronal activity using two-photon calcium imaging. Overnight, we trained an ensemble of CNNs to reproduce the measured neuronal responses and synthesized artificial stimuli for each target neuron in silico. On day 2, these stimuli were presented to the same neurons in vivo to compare measured and predicted responses. **c**, We presented 5,100 unique natural images to an awake mouse for 500 ms each, interleaved with gray screen gaps of random length between 300 and 500 ms. A subset of 100 images was repeated ten times to estimate neuronal response reliability. Neuronal activity in V1 L2/3 was recorded at 8 Hz using wide-field two-photon microscopy. Behavioral traces including pupil dilation and locomotion velocity were also recorded. **d**, CNN model architecture schematic. The network is composed of a three-layer convolutional core with a single-point readout predicting neuronal responses, a shifter network accounting for eye movements and a behavioral modulator predicting neuron-specific adaptive gain^7,57^. Average responses (gray) to test images for two example neurons are plotted with corresponding model predictions (black). **e**, Performance of the model ensemble, measured as the normalized correlation coefficient between predicted and observed responses to the 100 held-out images (CC_norm_)^12^. Data were pooled over 33,714 neurons from 14 mice (median 0.71, dashed line). Excessively noisy neurons (CC_max_ < 0.1) were excluded (0.2% of all neurons). Neurons with CC_norm_ outside [0, 1] were clipped (1.2%) for visualization. Source data

Using the trained model as a ‘digital twin’, we synthesized, for each neuron, a set of stimuli that elicited strong responses while being maximally different from one another called ‘varied exciting inputs’ (VEIs; Fig. 1a). Variation across a neuron’s VEIs reveals the visual features that define its invariances. To validate these model-generated predictions, we closed the loop by presenting VEIs back to the animal while recording the activity of the same neurons in vivo. Our results confirm the model’s predictions, demonstrating that VEIs reliably evoke strong responses in their target neurons.

The structure of the VEIs reveals a new functional invariance in V1 neurons, which we refer to as ‘bipartite invariance’. These VEIs partition the receptive field (RF) into two distinct, nonoverlapping subfields: a variable subfield that responds robustly to different crops from a preferred texture and a fixed subfield that responds strongly only to a particular spatial pattern. These neurons also prefer stimuli in which the two subfields differ in spatial frequency content, with the variable subfield biased toward higher spatial frequencies. This spatial and frequency division suggests that bipartite V1 neurons may serve as specialized detectors of object boundaries defined by abrupt changes in texture and spatial frequency.

Finally, we adapted our methodology to analyze the MICrONS functional connectomics dataset^8^ using a state-of-the-art foundation model^9^. This analysis reveals a hierarchical organization among excitatory neurons in V1 layers 2/3 (L2/3) in which postsynaptic neurons exhibit greater invariance than their presynaptic partners, and neurons with lower invariance form more synapses than those with higher invariance. Our findings collectively suggest a new principle of RF organization in the mouse primary visual cortex, offering new insights into how the brain might extract visual features from complex backgrounds and advancing our understanding of circuit-level mechanisms underlying neuronal invariance.

## Results

### VEIs identify neuronal invariances

In this study, we employed inception loops^7,10^, a closed-loop experimental paradigm in which we first recorded neuronal responses to natural images, then trained a deep predictive model and used it to synthesize stimuli that were subsequently presented back to the same neurons to investigate single-neuron invariances in mouse V1 (Fig. 1b).

We presented 5,100 unique natural images from ImageNet (ILSVRC2012)^11^ to awake, head-fixed mice while recording the activity of thousands of V1 L2/3 excitatory neurons using two-photon calcium imaging (Fig. 1c). We used the recorded neuronal activity to train convolutional neural networks (CNNs) to predict the responses of these neurons to arbitrary natural images (Fig. 1d). Model performance, assessed on held-out repeated images using a noise-normalized correlation metric^12^(Supplementary Fig. 1), reached a median of 0.71 across 33,714 neurons, comparable to state-of-the-art mouse V1 models^13–15^ (Fig. 1e).

We adapted and extended recently developed optimal stimulus synthesis frameworks to map both the selectivity^7,10^ and invariance^16^ of individual neurons in silico. In our study, ‘selectivity’ refers to the specific image features eliciting maximal neuronal responses, while ‘invariance’ denotes image variations preserving high response magnitude. Expanding on our previous work^7^, which identified a single most exciting input (MEI) for each neuron, we now generate a set of 20 VEIs (Fig. 1a) to characterize neuronal functional invariance. These VEIs, which we also refer to as ‘nonparametric VEIs’ for ease of comparison in subsequent analyses, are defined as images that are maximally dissimilar in the pixel space but all strongly activate the target neuron, with each VEI constrained to elicit at least 85% of the MEI response in silico (Methods).

**Fig. 2:**
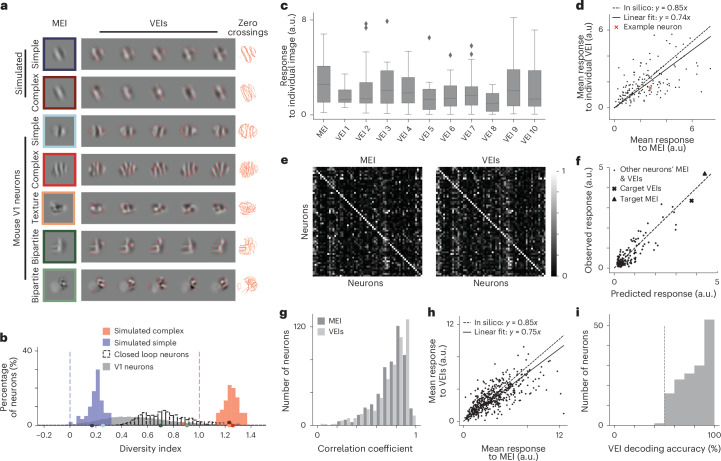
VEIs evoked strong and selective responses in target neurons while exhibiting population-decodable differences. **a**, Examples of MEI and VEIs for simulated simple and complex cells, and mouse V1 neurons. For each neuron, zero-crossing contours from individual VEIs (locations where the image intensity transitions from positive to negative values or vice versa) were overlaid. **b**, Diversity indices for 60 simulated complex cells (red), 60 simulated simple cells (blue) and 10,228 V1 neurons pooled from 14 mice (gray), including 500 tested in closed-loop experiments from eight mice (unfilled). Diversity index is defined as the normalized average pairwise Euclidean distance in pixel space across the VEIs. Diversity indices for noiseless simple cells (0, blue dashed) and complex cells (1, red dashed) were shown for reference. V1 neuron diversity indices differed from simulated simple and complex cells (*P* = 3.1 × 10^−49^ and 1.2 × 10^−67^, two-sided Welch’s *t*-test with 72.4 and 69.0 d.f., respectively). For closed-loop experiments, we randomly selected neurons with high diversity indices (Methods). Example neurons from **a** were indicated on the x axis with the corresponding colors. Diversity indices <−0.25 were clipped to −0.25 for visualization (0.09% of all V1 neurons). **c**, Response of an example neuron to its MEI and ten random VEIs. Both MEI and individual VEI were averaged across 20 repeats. Only two out of the ten VEIs elicited responses lower than 85% of the MEI response (one after Benjamini–Hochberg (BH) correction for multiple comparison). **d**, Comparison of mean responses to MEI and one random VEI per neuron. VEIs stimulated in vivo responses in target neurons close to the level predicted in silico relative to MEI (74 ± 4% versus 85%) (two-sided Wilcoxon signed-rank test, *W* = 4,902, *P* = 0.19) with only 274 of 1,490 VEIs (18.4%) showing responses lower than 85% of the corresponding MEI response (3.0% after BH correction) (*P* < 0.05, one-sided Welch’s *t*-test with 32.6 average d.f.). Data were pooled over 149 neurons from two mice. **e-h**, VEI responses were averaged across 20 different VEIs with each presented once. **e**, Both MEI and VEIs activated neurons with high specificity. Confusion matrices showed responses of each neuron to MEI (left) and VEIs (right) for 61 neurons in one mouse. Responses of each neuron were normalized, with each row scaled so the maximum response across all images equaled 1. Neurons’ responses to their own MEI and VEIs (along the diagonal) were larger than those to other MEIs and VEIs, respectively (two-sided permutation test, *P* < 10^−4^ for both cases). **f**, Predicted versus observed responses of one example neuron to its own MEI and VEIs and 79 other neurons’ MEI and VEIs. **g**, Our model exhibited high predictive accuracy for both MEI and VEI responses (Pearson correlation coefficient between predicted and observed neuronal responses *r* = 0.74 and 0.75, respectively). **h**, VEIs stimulated in vivo responses close to the level predicted in silico relative to MEI (75 ± 3% versus 85%) (two-sided Wilcoxon signed-rank test, *W* = 51,360, *P* = 4.9 × 10^−4^), with only 9.6% of all neurons showing different responses between VEIs and 85% of MEI (1.2% after BH corrections) (*P* < 0.05, two-sided Welch’s *t*-test with 34.06 average d.f.). **g**,**h**, Data were pooled over 500 neurons from eight mice. **i,** In vivo population responses in mouse V1 L2/3 discriminated between a randomly selected pair of VEIs for each neuron. VEI identity in individual trials was decoded using a logistic regression classifier (see Methods for details), with decoding accuracies across neurons (median 80%) exceeded chance level (50%, dashed; one-sample *t*-test, *t* = 28.0, *P* = 5.0 × 10^−61^). Data were pooled over 149 neurons from two mice. Source data

Our VEI synthesis method successfully reproduced the expected functional invariances in simulated Hubel and Wiesel simple and complex cells (Fig. 2a). For simulated simple cells, VEIs resembled Gabor patches with identical orientation, spatial frequency and phase (Fig. 2a, simulated simple), aligning with linear–nonlinear model predictions^17,18^. In contrast, simulated complex cell VEIs included Gabor patches with different phases, reflecting their known phase invariance (Fig. 2a, simulated complex).

VEIs from mouse V1 neurons strongly resembled their corresponding MEIs, while exhibiting specific variations indicative of different invariance types (Fig. 2a, mouse V1 neurons; see more examples in Supplementary Fig. 2). Some neurons produced nearly identical VEIs, suggesting a lack of invariance akin to simulated simple cells (Fig. 2a, mouse simple). A small subset of V1 neurons exhibited VEIs with varying phases while maintaining consistent orientation and spatial frequency, closely resembling the behavior of simulated complex cells (Fig. 2a, mouse complex).

Among neurons strongly activated by non-Gabor stimuli^7,13^, some were strongly activated by VEIs that appeared as patches sampled from a common underlying texture canvas, demonstrating global shift invariance (Fig. 2a, mouse texture). We termed these neurons ‘texture cells’, in analogy to similar units observed in hidden layers of deep artificial neural networks trained for object recognition^16,19^. Of note, many neurons exhibited a new type of invariance that we denoted as ‘bipartite RF invariance’ or equivalently, ‘bipartite invariance’, where one portion of their RF preferred a fixed spatial pattern, while the other responded robustly to different spatial translations of a specific texture image (Fig. 2a, mouse bipartite). In other words, the neuron’s response to the variable subfield remained strong when different crops of an underlying texture canvas were presented. We referred to these neurons as ‘bipartite cells’. To quantify these phenomena, we computed a diversity index for each neuron based on the average pairwise dissimilarity among its VEIs (Methods). The diversity indices of mouse V1 neurons spanned a continuous spectrum, with those of simulated simple and complex cells at the opposite extremes (Fig. 2b).

**Extended Data Fig. 1:**
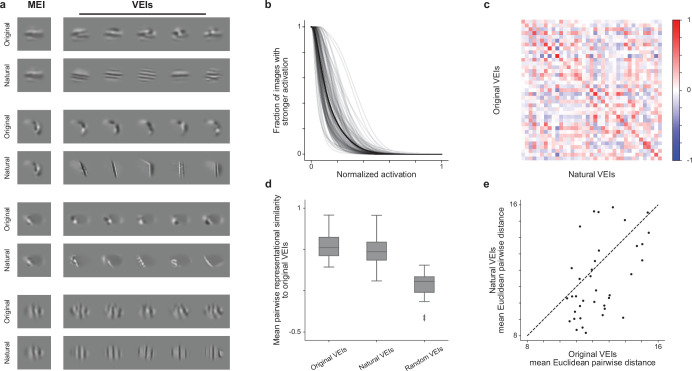
VEIs capture invariance observed in natural images with VEI-like activation. **a**, Examples of MEI, VEIs, and ‘natural VEIs’ for 4 example neurons. **b**, Neuronal responses to masked natural images are sparse, and their amplitudes are smaller than those evoked by MEIs and VEIs. The gray lines show the fraction out of 41 million masked images that elicit a given activation or higher for 100 model target neurons; black is the average. Responses from each cell are divided by the response to its MEI. On average, 1.2% of images produced activations above 50%, 0.02% above 75%, and 0.006% above 85% of the MEI activation. **c**, Natural VEIs maintained high specificity to their target neuron. Confusion matrices showed in silico representational similarity between original VEIs and highly activating natural crops. Representational similarity between original VEIs and highly activating natural crops for the same neurons (diagonal) was larger than cross-neuron similarity (off-diagonal) (two-sided permutation test, *P* < 10^−4^ for all conditions after BH corrections). **d**, Natural VEIs closely resembled the original VEIs. The original VEIs were more similar to natural VEIs of the same neuron than original VEIs of other random neurons (two-sided Wilcoxon signed-rank test, *W* = 0, *P* = 1.1 × 10^−7^). **e**, Original VEIs have higher mean Euclidean pairwise distances than those of natural VEIs (two-sided Wilcoxon signed-rank test, *W* = 179, *P* = 0.01). Data were pooled over 100 neurons randomly sampled from eight mice. Source data

To assess whether the invariances captured by VEIs also appear in natural images, we screened over 41 million crops to identify those that elicited VEI-like activation (Extended Data Fig. 1a). We found that only a small fraction (0.006%) of these images produced responses comparable to VEIs in silico (≥85% of the MEI activation; Extended Data Fig. 1b) and only 37% of neurons yielded more than 20 such highly activating natural stimuli. Notably, the highly activating natural crops closely resembled VEIs (Extended Data Fig. 1c,d), albeit with lower diversity (Extended Data Fig. 1e), highlighting the extreme lifetime sparsity of the neural code^20^. Collectively, these findings suggest that VEIs effectively capture naturally occurring invariances.

**Extended Data Fig. 2:**
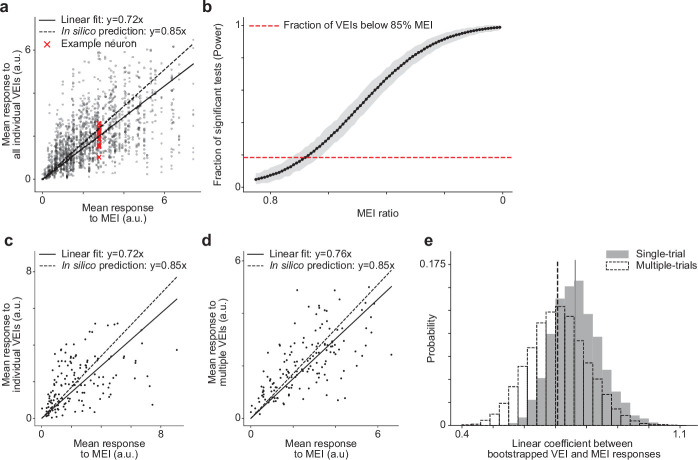
Individual VEI activated their target neuron strongly. **a**, For each neuron, we randomly selected ten VEIs (red) from the set of 20 VEIs. Individual VEIs stimulated in vivo closely to the level predicted in silico with respect to MEI (72 ± 4% versus 85%) (two-sided Wilcoxon signed-rank test, *W* = 519434, *P* = 0.03), with only 274 out of all 1490 individual VEIs (18.4%) evoked responses lower than 85% of the corresponding MEI (3.0% after BH correction; *P* < 0.05, one-sided Welch’s *t*-test with 32.5 average d.f.). **b**, Fraction of correctly rejected null hypotheses as a function of response reduction from the 85% threshold; the shaded region indicates the 95% confidence interval. **c,d**, To quantify the in vivo response relationship between MEI and VEIs, we performed bootstrapping by either averaging across 20 randomly selected trials from a single VEI (**c**) or 20 randomly selected trials from 10 different VEIs (**d**) (Methods). **e**, The linear coefficients estimated using individual VEI (**c**, median=0.71) were similar to those estimated using multiple VEIs (**d**, median=0.76) (*P* = 0.59, two-sided bootstrapped mean difference against 0). Data were pooled over 149 neurons from two mice. Source data

To test whether model-synthesized VEIs indeed elicit strong neuronal responses as predicted, we presented MEIs and VEIs back to the same neurons in awake mice under two protocols that varied the number of VEIs and repeats per image: (1) randomly selecting ten VEIs from the set of 20 VEIs synthesized per neuron and presenting each stimulus 20 times; and (2) presenting all 20 VEIs once for each neuron (Methods). Across all closed-loop experiments, individual VEIs robustly activated their target neurons in vivo (Fig. 2c,d and Extended Data Fig. 2a). Figure 2c illustrates this for one example neuron, with only two out of ten VEIs eliciting responses lower than 85% of the MEI response. Across all neurons tested in two mice, only 274 out of 1,490 VEIs (18.4%) elicited responses lower than 85% of their corresponding MEI. Power analysis based on resampled MEI trials suggested that individual VEI responses typically ranged between 64 and 73% of the MEI response (95% CI at power 0.184; Extended Data Fig. 2b). Consistently, when searching 41 million natural image patches, only 0.17% produced responses exceeding 64% of the MEI response, highlighting the extreme sparsity of high-activating stimuli in natural vision. We also evaluated whether a set of 20 VEIs, each presented once, elicited the same overall activation as a single randomly selected VEI presented 20 times. Our results demonstrated that this was the case, validating that VEI sets provide a reliable measure of neuronal activation (Extended Data Fig. 2c--e). In subsequent experiments, we utilized VEI sets alongside control stimuli and systematic manipulations of VEIs to investigate their collective properties.

Similar to MEIs, VEIs were selective for the neurons they were optimized to activate, consistently eliciting higher activity in their target neurons compared to non-target neurons (Fig. 2e and Supplementary Fig. 3). In addition, the digital twin accurately predicted the magnitude of neuronal responses to synthesized MEIs and VEIs, yielding median Pearson correlation coefficients of 0.74 and 0.75, respectively, between predicted and observed responses (Fig. 2f,g), further validating our approach. Of note, VEIs strongly activated their target neurons in vivo, achieving 75 ± 3% of their corresponding MEI activation when responses were averaged across sets of 20 distinct VEIs, each presented once (Fig. 2h), close to the model prediction of 85%. This effect remained robust after controlling for eye movements (Supplementary Fig. 4).

One potential concern was that differences across VEIs might be indistinguishable to the animal, given the spatial acuity limits of the mouse visual system. To address this, we presented one randomly selected VEI pair per neuron and used the in vivo V1 population activity to decode VEI identity with a logistic classifier. Decoding accuracy substantially exceeded chance (median 80%; Fig. 2i), demonstrating that the V1 population can reliably discriminate between VEIs and that the observed single-neuron invariances correspond to perceptually accessible image transformations.

**Fig. 3:**
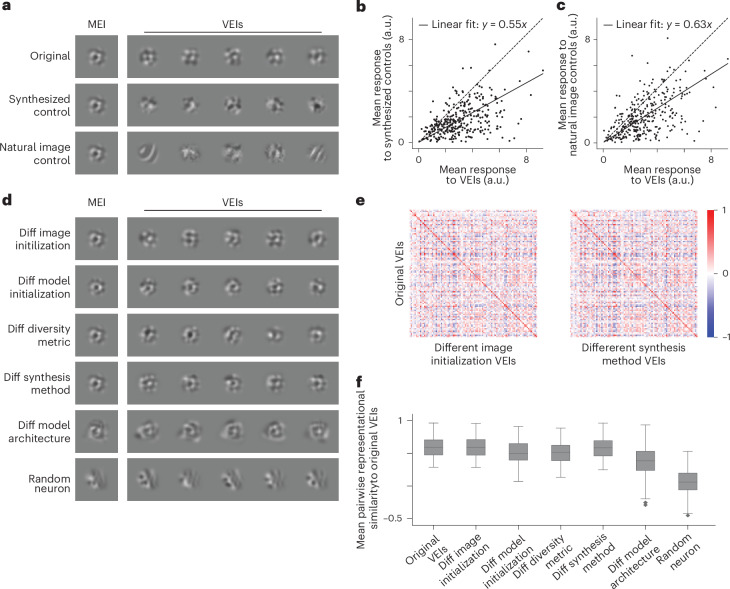
VEIs evoked stronger responses than controls and generalized across different synthesis conditions. **a**, MEI, VEIs (top), synthesized controls (middle) and natural controls (bottom) for one example neuron. Synthesized controls were generated by perturbing MEI in random directions, while natural controls were selected by searching through random natural patches. For each neuron, both controls were restricted to be closer to the MEI than all the VEIs as measured by Euclidean distance in pixel space. **b**, Synthesized controls failed to stimulate their target neurons in vivo compared to VEIs (55 ± 2% of VEI activation, two-sided Wilcoxon signed-rank test, *W* = 3,258, *P* = 2.3 × 10^−41^), with 36.8% neurons showing lower responses to synthesized controls compared to VEIs (20.8% after BH corrections; *P* < 0.05, two-sided Welch’s *t*-test with 30.4 average d.f.). **c**, Natural controls failed to stimulate their target neurons in vivo compared to VEIs (63 ± 3% of VEI activation, two-sided Wilcoxon signed-rank test, *W* = 6,442, *P* = 9.4 × 10^−31^), with 31.5% neurons showing lower responses to natural controls compared to VEIs (16.0% after BH corrections; *P* < 0.05, two-sided Welch’s *t*-test with 31.1 average d.f.). **b**,**c** Response to each stimulus type was averaged over 20 different images with single repeat. Data were pooled from 318 neurons across five mice. **d**, MEI and VEIs for the same neuron in **a**, synthesized under various conditions: (1) different image initialization, (2) different model initialization, (3) different diversity metric, (4) different synthesis method^21^, and (5) different model architecture^14^. **e**, VEIs synthesized under different conditions maintained high specificity to their target neuron. Confusion matrices showed in silico representational similarity between original VEIs and VEIs from different image initialization (left) or VEIs from a different synthesis method^21^ (right) (for other conditions, see Extended Data Fig. 4a). Each entry represents the mean pairwise cosine similarity between two sets of VEIs (Methods). Representational similarity between original VEIs and VEIs synthesized from different conditions for the same neurons (diagonal) was larger than cross-neuron similarity (off-diagonal) (two-sided permutation test, *P* < 10^−4^ for all conditions after BH corrections). **f**, VEIs synthesized under different conditions closely resembled the original VEIs. The original VEIs were more similar to VEIs generated from various modifications in **d** than random neurons’ VEIs generated using the original method (two-sided Wilcoxon signed-rank test, *W* = 0, 0, 1, 0, 0, 426, *P* = 1.2 × 10^−17^, 1.2 × 10^−17^, 1.3 × 10^−17^, 1.2 × 10^−17^, 1.2 × 10^−17^, and 2.2 × 10^−17^, respectively, after BH correction). Box plots show center line (median); box bounds (25th to 75th percentiles, IQR); whiskers extend to the most extreme data points within 1.5 × IQR of the quartiles; caps mark whisker ends; points beyond whiskers are plotted as outliers. **e**,**f**, Data were pooled from 97 neurons randomly sampled across eight mice. Source data

We next asked whether VEIs simply lie near the MEI in image space or instead follow specific directions that preserve activation. As controls, we constructed two sets of images matched in pixel-wise distance to the MEI: synthetic perturbations along random directions from the MEI (equation (6)) and natural image patches close to the MEI in the pixel space (Fig. 3a and Methods). Both control sets drove substantially weaker responses than VEIs in closed-loop experiments (Fig. 3b,c), indicating that VEIs capture specific directions in the image manifold along which activation is preserved, rather than generic proximity to the MEI.

**Extended Data Fig. 3:**
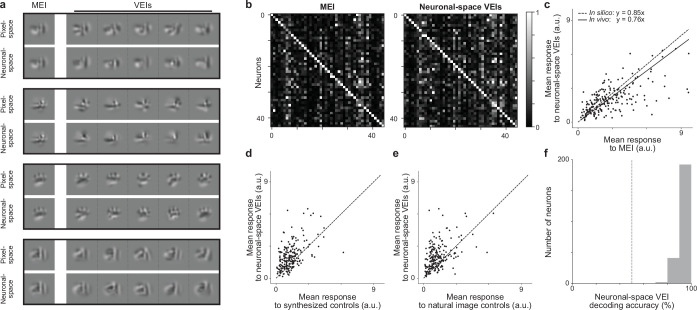
Neuronal-space VEIs evoked strong and selective in vivo responses in target neurons while exhibiting population-decodable differences. **a**, Examples of MEI and VEIs synthesized with image diversity evaluated by Euclidean distance in pixel space (‘pixel-space’) or by cosine distance in the in silico population neuronal response space (‘neuronal-space’) for four example neurons. **b**, The confusion matrices showed the responses of each neuron to MEI (left) and neuronal-space VEIs (right) of 44 neurons. The responses of each neuron were normalized, and each row was scaled so the maximum response across all images equals 1. Neurons responded more strongly to their own MEI and neuronal-space VEIs (along the diagonal) compared to MEIs and neuronal-space VEIs from other neurons, respectively (two-sided permutation test, *P* < 10^−4^ for both cases). **c**, Neuronal-space VEIs stimulated in vivo closely to the level predicted in silico with respect to MEI (76 ± 3% versus 85%) (two-sided Wilcoxon signed-rank test, *W* = 8464, *P* = 7.7 × 10^−3^), with only 11.6% of all neurons showing different responses between neuronal-space VEIs and 85% of MEI (0.48% after BH correction; *P* < 0.05, two-sided Welch’s *t*-test with 32.61 average d.f.). **d,e**, Neuronal-space VEIs activated their target neurons more strongly than synthesized (**d**) and natural image (**e**) controls (two-sided Wilcoxon signed-rank test, *W* = 3441, *P* = 2.1 × 10^−17^ and *W* = 3466, *P* = 2.7 × 10^−17^ respectively) with 13.5% and 19.8% of all neurons showing higher responses to neuronal-space VEIs (0.0% and 2.5% after BH correction; *P* < 0.05, two-sided Welch’s *t*-test with 31.0 and 30.6 average d.f., respectively). **c-e**, Data were pooled over 207 neurons from five mice. **f**, In vivo population responses in mouse V1 L2/3 discriminated between the most dissimilar pair of neuronal-space VEIs. VEI identity in individual trials was decoded using a logistic regression classifier, with decoding accuracies across neurons (median 93%) exceeded chance level (50%, dashed line; one-sample *t*-test, *t* = 138.5, *P* = 1.7 × 10^−180^). Data were pooled over 235 neurons from three mice. Source data

**Extended Data Fig. 4:**
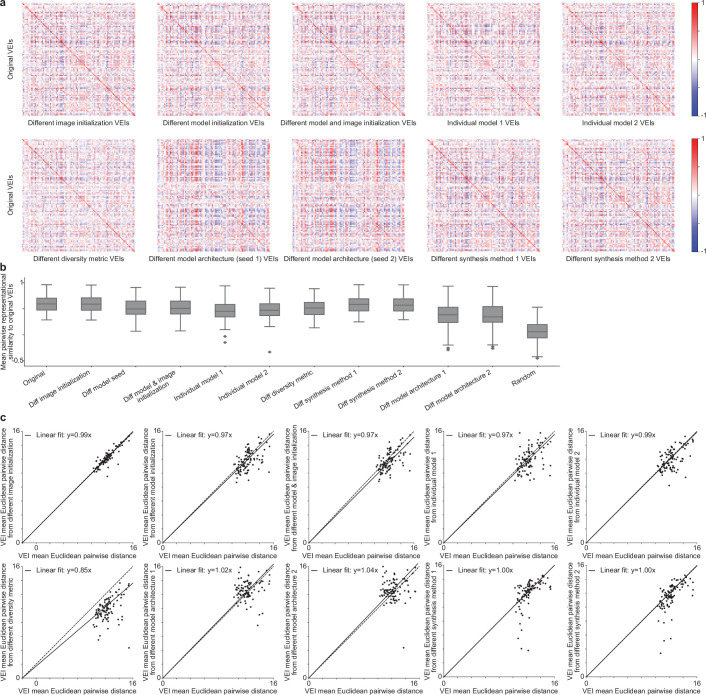
VEIs generalized across different conditions. **a**, VEIs synthesized under different conditions remained highly specific to their target neurons. The confusion matrices showed the representational similarity between the original VEIs and VEIs generated from (1) different image initializations for VEIs synthesis, (2) different model initializations, (3) different image and model initializations, (4,5) synthesis using a single model from the ensemble, (6) a different diversity metric based on in silico population responses, (7,8) a different model architecture^14^ with two different model seeds, (9,10) a different synthesis method using implicit 1D and 2D periodic latent space neural representation model and contrastive regularization^21^. Each entry represents the mean pairwise cosine similarity between two sets of VEIs (Methods). Representational similarity between original VEIs and VEIs synthesized from different conditions for the same neurons (diagonal) was larger than cross-neuron similarity (off-diagonal) (two-sided permutation test, *P* < 10^−4^ for all conditions after BH correction). **b**, VEIs synthesized from different conditions were more similar to original VEIs than random neurons’ VEIs (two-sided Wilcoxon signed-rank test, *W* = 0, 0, 1, 1, 34, 0, 0, 0, 0, 426, and 357, with *P* = 1.2 × 10^−17^, 1.2 × 10^−17^, 1.3 × 10^−17^, 1.3 × 10^−17^, 3.5 × 10^−17^, 1.2 × 10^−17^, 1.2 × 10^−17^, 1.2 × 10^−17^, 1.2 × 10^−17^, 2.2 × 10^−17^, and 3.7 × 10^−17^, respectively, after BH correction). **c**, The mean Euclidean pairwise distances between VEIs generated using different methods were strongly correlated with those of the original VEIs (Pearson *r* = 0.89, 0.56, 0.60, 0.43, 0.54, 0.45, 0.18, 0.08, 0.62 and 0.66, with *P* = 1.5 × 10^−34^, 2.5 × 10^−9^, 2.2 × 10^−10^, 1.3 × 10^−5^, 1.4 × 10^−8^, 3.5 × 10^−6^, 0.07, 0.41, 1.4 × 10^−11^, and 1.2 × 10^−13^, respectively, two-sided *t*-test). Data were pooled over 97 neurons randomly sampled from eight mice. Source data

Finally, we assessed the robustness of VEIs to changes in synthesis conditions. We varied image and model initialization, the diversity metric (pixel space versus a neuronal population latent space), the synthesis method (including an implicit neural representation model), and the predictive model architecture^14,21^ (Fig. 3d and Methods). Across these manipulations, VEIs remained highly specific to their target neurons and showed high representational similarity to the original VEIs, while preserving comparable diversity (Fig. 3e,f and Extended Data Figs. 3 and 4). These results indicate that the VEI-based invariances reflect intrinsic neuronal properties rather than artifacts of a particular model or synthesis pipeline.

### Bipartite parameterization of VEIs

**Fig. 4:**
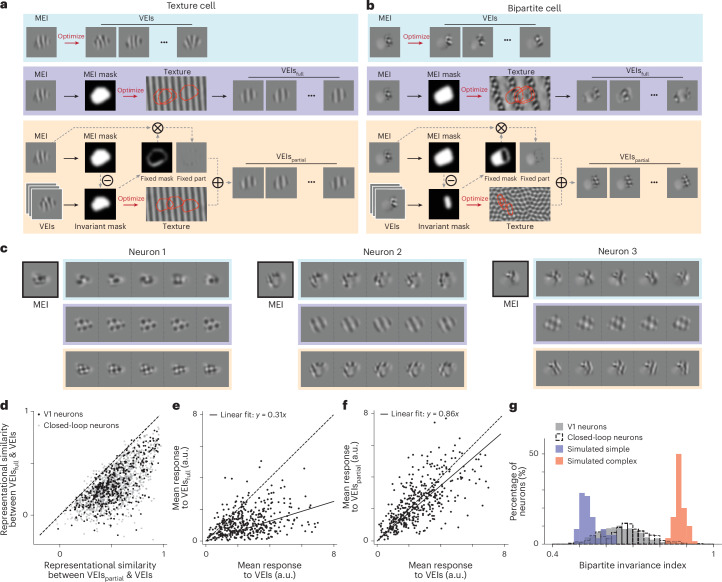
Bipartite parameterization reproduces the visual features and in vivo responses of nonparametric VEIs. **a**,**b**, Schematic of VEI synthesis using the nonparametric approach (VEIs, blue), full-texture parameterization (VEIs_full_, purple), and partial-texture parameterization (VEIs_partial_, orange) for an example V1 texture cell (left) and V1 bipartite cell (right). VEIs_full_ were synthesized by optimizing an underlying texture canvas, from which random crops masked by the MEI mask maximally activated the target neuron. In contrast, VEIs_partial_ comprised two distinct, nonoverlapping subfields: a fixed subfield directly masked from the MEI, and a shift-invariant subfield preferring random crops from a texture image synthesized similarly to VEIs_full_, but using only part of the MEI mask for texture optimization. **c**, MEI, VEIs, VEIs_full_ and VEIs_partial_ for three example neurons, with each VEI type indicated by the corresponding color from **a**. **d**, VEIs_partial_ were more similar to their corresponding nonparametric VEIs than VEIs_full_ for both random V1 neurons and closed-loop neurons (two-sided Wilcoxon signed-rank test, *W* = 2,783, *P* = 4.6 × 10^−195^ and *W* = 65, *P* = 3.0 × 10^−67^, respectively). **e**, VEIs_full_ failed to stimulate their target neurons in vivo compared to nonparametric VEIs (31 ± 2% of VEI activation, two-sided Wilcoxon signed-rank test, *W* = 4,389, *P* = 6.2 × 10^−54^) with 43.4% of all neurons showing different responses to VEIs_full_ than VEIs (29.4% after BH corrections) (*P* < 0.05, two-sided Welch’s *t*-test with 29.4 average d.f.). **f**, VEIs_partial_ activated their target neurons in vivo similarly to nonparametric VEIs (86 ± 4% of VEI activation, two-sided Wilcoxon signed-rank test, *W* = 32,429, *P* = 7.0 × 10^−4^) with only 8.5% of all neurons showing different responses (0.0% after BH corrections) (*P* < 0.05, two-sided Welch’s *t*-test with 33.5 average d.f.). **e**,**f**, In vivo responses to VEIs, VEIs_full_ and VEIs_partial_ were averaged across 20 different images with single repeat. **g**, Bipartite invariance indices of V1 neurons were larger than those of simulated simple cells (60 cells, blue) and lower than those of simulated complex cells (60 cells, red) (*P* = 1.4 × 10^−38^ and 1.1 × 10^−138^, two-sided Welch’s *t*-test with 95.5 and 213.8 d.f., respectively). Data were pooled from six mice, displaying a total of 1,200 neurons for random V1 neurons; closed-loop neurons comprised 401 neurons pooled from eight mice. Source data

To move beyond the qualitative description of bipartite invariances, we next developed simple quantitative models with interpretable parameters. We first modeled global shift invariance by synthesizing, for each neuron, a full-field texture that maximized the average in silico response to random crops within its RF, extending previous work from Cadena et al.^16^ (Fig. 4a,b, middle rows). Random crops from this optimized texture (‘full-texture VEIs’, VEIs_full_) captured neurons whose entire RF was approximately shift-invariant, akin to classical complex cells. For many neurons, this global shift-invariant model proved inadequate and produced stimuli that deviated visually from the original nonparametric VEIs (Fig. 4b,c, middle versus top rows). This suggested a more nuanced form of invariance in V1 neurons with heterogeneous RFs (Fig. 2a,b). We therefore introduced a ‘partial shift invariance’ model that parameterized VEIs as the sum of two nonoverlapping subfields within the RF: a fixed subfield, taken directly from the MEI and held constant across VEIs, and a variable subfield in which different crops of an optimized texture maintained high responses (Fig. 4a,b, bottom rows).

**Extended Data Fig. 5:**
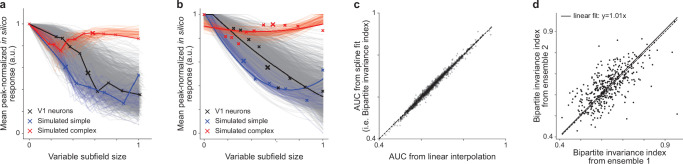
Bipartite invariance quantification. **a,b**, Mean peak-normalized in silico response as a function of the variable subfield size with linear interpolation (**a**) and quadratic-smoothing spline (**b**) for 1200 random V1 neurons from six mice, 60 simulated simple cells (blue), and 60 simulated complex cells (red). **c**, Bipartite invariance index was defined as the ‘area under the curve” (AUC) from the quadratic-smoothing spline in **b**. AUC values were highly consistent regardless of whether linear interpolation or quadratic-smoothing spline was used (Pearson *r* = 0.995, *P* < 10^−6^, two-sided *t*-test). **d**, Bipartite invariance indices were consistent across model ensembles initialized with different random seeds (Pearson *r* = 0.66, *P* = 3.2 × 10^−52^, two-sided *t*-test). **a–d**, Data were pooled over 1200 neurons from six mice. Source data

To identify the fixed and variable subfields, we used the spatial variance pattern across nonparametric VEIs to define a candidate variable subfield and its complement as the fixed subfield; for each candidate variable subfield size, we optimized a corresponding texture and generated texture-based VEIs by combining texture crops with the fixed MEI subfield (Methods and Extended Data Fig. 5a). We quantified partial shift invariance with a bipartite invariance index (BII) that summarizes the trade-off between in silico activation and variable subfield size (Methods and Extended Data Fig. 5b,c). Simulated simple cells, complex cells, and V1 neurons exhibited low, high and intermediate BII values (medians 0.53, 0.87 and 0.65, respectively; Fig. 4g), with estimates robust across model ensembles (Extended Data Fig. 5d). For each neuron, we then selected the variable subfield by maximizing the harmonic mean of response strength and diversity of the resulting texture-based VEIs (equation (9)), defining the corresponding stimuli as ‘partial-texture VEIs’ (VEIs_partial_). Remarkably, VEIs_partial_ visually resembled the nonparametric VEIs more closely than VEIs_full_, as quantified by representational similarity (Fig. 4d). During closed-loop experiments, VEIs_partial_ activated neurons at levels comparable to nonparametric VEIs (86% of the VEI response), whereas VEIs_full_ elicited much weaker responses (31% of the VEI response) (Fig. 4e,f). Notably, we still observed strong in vivo responses to both VEIs_partial_ and nonparametric VEIs even after excluding neurons whose VEIs_partial_ were dominated by fixed subfields resembling the MEI (Supplementary Fig. 5).

**Extended Data Fig. 6:**
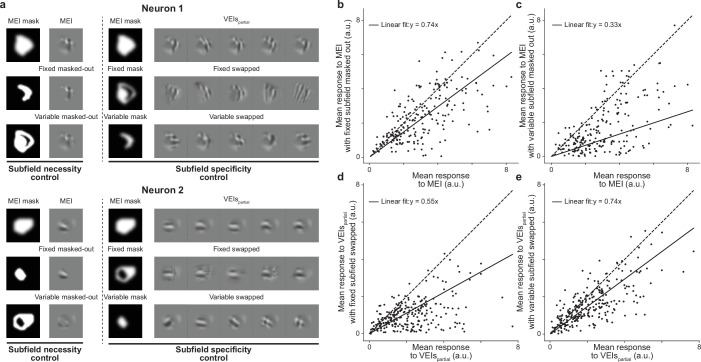
Both subfields of the partial-texture VEIs are necessary and specific for evoking high in vivo responses. **a**, Examples of the MEI, MEI with either fixed or variable subfield masked out, VEIs_partial_, and VEIs_partial_ with either fixed or variable subfield swapped with random content for two example neurons. The fixed subfield content was replaced with 20 different random natural patches while the variable subfield was replaced with patches cropped from 20 different random non-self neurons’ preferred textures. **b,c**, MEI with fixed (**b**) or variable (**c**) subfield masked out evoked weaker in vivo responses than MEI (two-sided Wilcoxon signed-rank test, *W* = 5347, *P* = 7.0 × 10^−12^ and *W* = 1570, *P* = 4.1 × 10^−28^, respectively) with 15.4% and 38.1% of all neurons showing weaker responses than their MEIs (1.9% and 27.9% after BH correction) (*P* < 0.05, two-sided Welch’s *t*-test with 33.2 and 29.9 average d.f., respectively). Data were pooled over four mice, displaying a total of 215 neurons. **d,e**, VEIs_partial_ with either fixed (**d**) or variable (**e**) subfield swapped evoked weaker in vivo responses than VEIs_partial_ (two-sided Wilcoxon signed-rank test, *W* = 1960, *P* = 6.5 × 10^−35^, and *W* = 5820, *P* = 7.0 × 10^−20^, respectively) with 30.1% and 12.7% of all neurons showing weaker responses than their partial-texture VEIs (18.9% and 0.0% after BH correction) (*P* < 0.05, two-sided Welch’s *t*-test with 30.0 and 32.5 average d.f., respectively). Data were pooled over 259 neurons from five mice. Source data

We next tested the necessity and specificity of the two subfields in VEIs_partial_ by isolating or swapping the content within each subfield (Extended Data Fig. 6a). We found that both subfields were necessary for high activation, masking out the fixed or variable subfield content from the MEI reduced in vivo responses in target neurons to 74% and 33%, respectively (Extended Data Fig. 6b,c). Similarly, the contents within both subfields were highly specific. Replacing the fixed subfield content with random natural image patches, or swapping the optimized texture for the variable subfield with textures from other neurons in VEIs_partial_ decreased activity to 55% and 74%, respectively (Extended Data Fig. 6d,e). While our closed-loop validation primarily focused on neurons exhibiting high levels of invariance, we also randomly selected neurons from all reliable and well-predicted V1 neurons (corresponding to 79.0% ± 0.5% of all neurons imaged per scan) for closed-loop verification. This confirmed that our findings generalized to the broader population (Supplementary Fig. 6).

**Extended Data Fig. 7:**
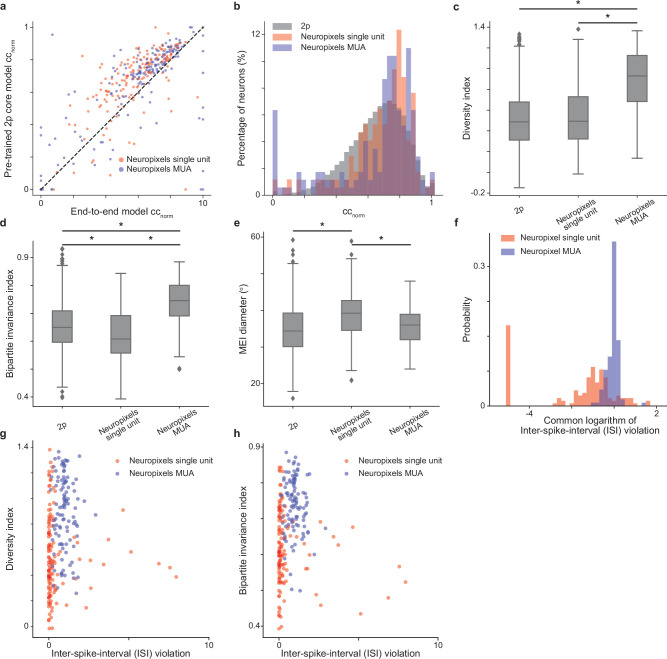
Quantification of diversity and bipartite invariance indices from electrophysiological data. **a**, Normalized correlation coefficients (*CC*_norm_) of the model fine-tuned from a pretrained core on two-photon recordings (denoted as ‘pretrained 2p core’) were higher than those from the model trained from scratch (denoted as ‘end-to-end’) (two-sided Wilcoxon signed-rank test, *W* = 10762, *P* = 2.1 × 10^−24^) with color representing units classified as either ‘single units’ or ‘multi-unit activity’ (denoted as ‘Neuropixels single’ and ‘Neuropixels multi-unit activity’ (Neuropixels MUA), respectively). **b**, Histogram of the normalized correlation coefficient (*CC*_norm_) for neurons modeled from two-photon recording (denoted as ‘2p’, data were pooled over 33,714 neurons from 14 mice), and Neuropixels units modeled using electrophysiological data with pretrained 2p core (data were pooled over 364 spikes sorted units from six mice). Excessively noisy neurons (*CC*_max_ < 0.1) were excluded (0.2%, 7.6%, and 4.6% of 2p, Neuropixels single, Neuropixels MUA, respectively) and neurons with values outside of 0 and 1 were clipped (1.2%, 4.5%, and 6.1% for 2p, Neuropixels single, and Neuropixels MUA, respectively) for visualization. **c**, Neuropixels MUA (0.88±0.29) exhibited larger diversity indices than Neuropixels single units (0.55±0.31) and 2p neurons (0.51±0.27) (*P* = 9.3 × 10^−16^ and 1.7 × 10^−37^, two-sided Welch’s *t*-test with 230.3 and 122.6 d.f., respectively) while Neuropixels single units had similar diversity indices to 2p neurons (*P* = 0.21, two-sided Welch’s *t*-test with 158.4 d.f.). **d**, Neuropixels MUA showed larger bipartite invariance indices (BIIs, 0.74±0.08) than Neuropixels single units (0.62±0.10) and 2p neurons (0.65±0.08) (*P* = 7.5 × 10^−21^ and 1.3 × 10^−22^, two-sided Welch’s *t*-test with 237.8 and 129.5 d.f., respectively) while Neuropixels single units had marginally lower bipartite invariance indices than 2p neurons (*P* = 2.8 × 10^−6^, two-sided Welch’s *t*-test with 155.7 d.f.). **e**, Neuropixels single units (38.8±6.36°) had larger RF sizes than both Neuropixels MUA (35.6±4.99°) and 2p neurons (34.9±6.64°) (*P* = 3.3 × 10^−5^ and 1.7 × 10^−10^, two-sided Welch’s *t*-test with 235.5 and 161.3 d.f., respectively) while MUA had similar RF sizes to those of 2p neurons (*P* = 0.30, two-sided Welch’s *t*-test with 141.6 d.f.). **f**, Histogram of the common logarithm of the inter-spike-interval (ISI) violations for Neuropixels single and Neuropixels MUA. Neuropixels MUA exhibited high ISI violations (median 0.94), whereas Neuropixels single units displayed a smaller mean but broader distribution of ISI violations (median 0.09). **g**, Diversity indices were not correlated with the ISI violations for Neuropixels single units (Pearson *r* = − 0.03 with *p* = 0.76). **h**, Bipartite invariance indices were not correlated with the ISI violations for Neuropixels single units (Pearson *r* = − 0.05 with *p* = 0.55). **g–h**, Units with excessively large ISI violation (> 10) were excluded for visualization (two single units and one MUA). **c–h**, Neurons were selected to have oracle scores larger than 0.22 and model test correlations larger than 0.42. Two-photon data were pooled over 1,154 neurons from six mice; electrophysiological data pooled over 240 spike sorted units from six mice. Source data

**Extended Data Fig. 8:**
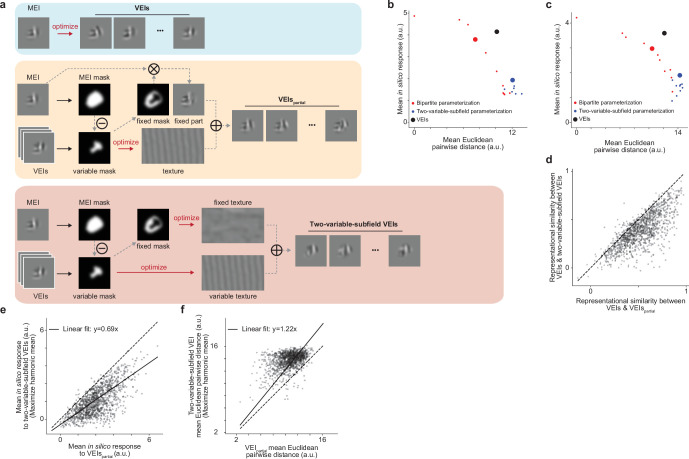
VEIs cannot be well explained by shift invariance in both subfields. **a**, Schematic illustrating three VEIs generation methods for an example V1 bipartite cell: nonparametric optimization (VEIs), bipartite parameterization (VEIs_partial_), and ‘two-variable-subfield’ parameterization. **b, c**, Example of in silico response and mean Euclidean pairwise distance for VEIs optimized using bipartite and two-variable-subfield parameterization. For each parameterization, we selected the sets of VEIs to maximize the harmonic mean between in silico response and image diversity (indicated by the larger dot). **d**, VEIs_partial_ were more similar to nonparametric VEIs than two-variable-subfield VEIs as measured by representational similarity (two-sided Wilcoxon signed-rank test, *W* = 23640, *P* = 5.7 × 10^−173^). **e**, VEIs_partial_ evoked higher responses in their target neurons in silico than two-variable-subfield VEIs (two-sided Wilcoxon signed-rank test, *W* = 1977, *P* = 1.1 × 10^−195^). **f**, VEIs_partial_ exhibited lower diversity than two-variable-subfield VEIs (two-sided Wilcoxon signed-rank test, *W =* 4842, *P* = 1.4 × 10^−192^). Data were pooled over 1200 randomly selected neurons from six mice. Source data

**Extended Data Fig. 9:**
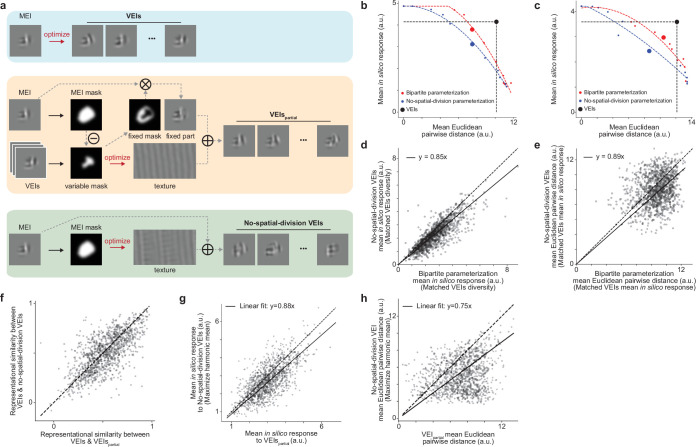
Spatial division is necessary for explaining VEIs. **a**, Schematic illustrating three VEI generation methods for an example V1 bipartite cell: nonparametric optimization (VEIs), bipartite parameterization (VEIs_partial_), and ‘no-spatial-division’ parameterization. **b, c**, Example of in silico response and mean Euclidean pairwise distance for VEIs optimized using bipartite and no-spatial-division parameterization. For each parameterization, we selected the sets of VEIs to maximize the harmonic mean between in silico response and image diversity (indicated by the larger dot). For each parameterization, a quadratic-smoothing spline fit was used to estimate: 1) the in silico response at the diversity level matching that of the nonparametric VEIs (denoted as ‘matched VEIs diversity’) and 2) the diversity level at the in silico response matching that of the nonparametric VEIs’ response (‘matched VEIs mean in silico response’). **d**, Bipartite parameterization evoked higher in silico responses than no-spatial-division parameterization when VEI diversity were matched (two-sided Wilcoxon signed-rank test, *W* = 82385, *P* = 1.4 × 10^−118^). **e**, Bipartite parameterization showed greater diversity than no-spatial-division parameterization when VEI mean in silico responses were matched (two-sided Wilcoxon signed-rank test, *W* = 89053, *P* = 5.3 × 10^−23^). **f**, VEIs_partial_ were more similar to the original nonparametric VEIs than no-spatial-division VEIs as measured by representational similarity (two-sided Wilcoxon signed-rank test, *W* = 293622, *P* = 2.8 × 10^−8^). **g**, VEIs_partial_ evoked stronger in silico responses than no-spatial-division VEIs (two-sided Wilcoxon signed-rank test, *W* = 199252, *P* = 5.1 × 10^−41^). **h**, VEIs_partial_ exhibited higher diversity compared to no-spatial-division VEIs (two-sided Wilcoxon signed-rank test, *W* = 241714, *P* = 5.5 × 10^−113^). Data were pooled over 1200 randomly selected neurons from six mice. Source data

**Extended Data Fig. 10:**
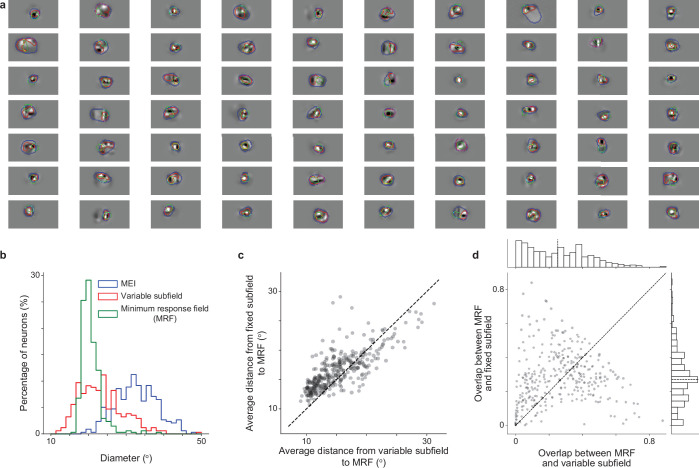
Bipartite receptive field cannot be explained by the center-surround structure. **a**, Example MEIs overlaid with their overall boundaries (blue), ‘Minimum Response Field’ (MRF) boundaries (green), and variable subfield boundaries (red) for visualization. **b**, Histogram of MEI, MRF, and variable subfield diameters. The mean diameters of MEI, MRF, and variable subfield across all neurons were (mean ± s.e.m.): 32.9 ± 0.02, 20.6 ± 0.01, 23.3 ± 0.02 degrees. **c**, The fixed subfield was located further from the MRF than variable subfield, as quantified by the mean pairwise distance between the MRF and each of the two subfields (two-sided Wilcoxon signed-rank test, *W* = 14278, *P* = 5.1 × 10^−16^). **d**, However, the fixed subfield overlapped more with the MRF than the variable subfield (two-sided Wilcoxon signed-rank test, *W* = 24373, *P* = 0.01; median = 27.1% and 25.1%, respectively). Data were pooled over 340 neurons from two mice. Source data

We also conducted several additional controls to rule out alternative explanations for the bipartite structure. First, Neuropixels recordings^22^ from mouse V1 showed similar diversity and BIIs to those measured with two-photon imaging, and these indices showed no dependence on inter-spike-interval (ISI) violations, a standard marker of spike contamination^23^, arguing against imaging artifacts or multi-unit contamination (Extended Data Fig. 7 and Supplementary Fig. 7). Second, alternative parameterizations that either allowed both subfields to be texture-modulated (‘two-variable-subfield’ models; Extended Data Fig. 8) or removed the spatial division between them (‘no-spatial-division’ models; Extended Data Fig. 9) produced stimuli that were less similar to the original VEIs and less effective at driving responses, indicating that the specific bipartite parameterization better captures the functional properties of these neurons. Third, analyses combining bipartite masks with classical RF structure showed that the bipartite organization cannot be explained by standard center-surround structure (Extended Data Fig. 10). Finally, VEIs and BIIs were stable across models trained on trials stratified by eye-movement amplitude, excluding trial-to-trial eye movements as a trivial source of the observed bipartition (Supplementary Fig. 8). Collectively, these findings demonstrate that V1 neurons’ highly activating input manifolds are best characterized by a bipartite structure, featuring one subfield that prefers a fixed spatial pattern and another that optimally responds to random crops of an underlying texture image.

### Bipartite structure aligns with natural object boundaries defined by spatial frequency differences

Previous studies have demonstrated that MEIs capture complex spatial features prevalent in natural scenes^7^. Given the bipartite RF organization revealed by VEIs_partial_, we asked whether mouse V1 neurons contribute to visual segmentation by preferentially responding to object boundaries defined by texture discontinuities^24^.

**Fig. 5:**
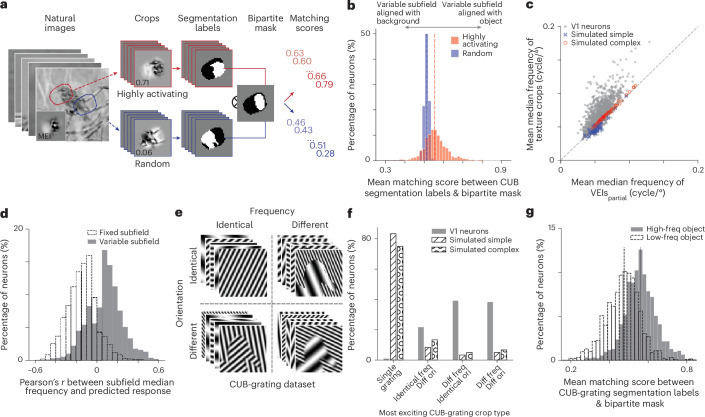
Bipartite structure aligns with natural object boundaries formed by spatial frequency differences. **a**, We screened over 1 million crops from the Caltech-UCSD Birds-200-2011 (CUB) dataset using our predictive model to identify the 100 most highly activating (red) and 100 random (blue) crops for each neuron. For each crop, we computed a matching score by comparing its segmentation label (object = white, background = black) and the neuron’s ‘bipartite mask’ derived from its VEIs_partial_ (variable subfield = white, fixed subfield = black). **b**, Highly activating natural crops with object boundaries yielded higher matching scores than random natural crops with object boundaries (two-sided Wilcoxon signed-rank test, *W* = 82,849, *P* = 6.5 × 10^−118^), with 51.4% of all neurons showing greater matching scores for highly activating crops than random natural crops (46.4% after BH correction) while only 4.9% showing lower matching scores to highly activating crops (4.1% after BH correction) (*P* < 0.05, two-sided Welch’s *t*-test with 76.2 average d.f.). One neuron (0.08%) was excluded from this analysis as it strictly preferred crops without object boundaries. **c**, Most V1 neurons preferred higher spatial frequency content in the variable subfield. The median frequency of texture crops exceeded that of VEIs_partial_ (two-sided Wilcoxon signed-rank test, *W* = 34,381, *P* = 3.1 × 10^−162^), with 77.0% of neurons showing higher median spatial frequency in the variable subfield than the fixed (76.4% after BH correction), and only 4.8% showing the opposite (4.8% after BH correction) (*P* < 0.05, two-sided Welch’s *t*-test with 33.2 average d.f.). In contrast, simulated simple cells (blue cross) preferred higher median frequency in the fixed subfield (two-sided Wilcoxon signed-rank test, *W* = 302, *P* = 6.4 × 10^−6^) and simulated complex cells (red circle) preferred higher median frequency in the variable subfield (two-sided Wilcoxon signed-rank test, *W* = 14, *P* = 3.3 × 10^−11^), albeit both with very marginal effect. **d**, Model-predicted V1 neuronal responses correlate with spatial frequency within the variable and fixed subfield. For the majority of neurons (79.08%), the fixed subfield’s median frequency negatively correlated with the predicted response (median −0.14, one-tailed one-sample *t*-test against mean of 0, *t* = −33.38, *P* = 3.7 × 10^−169^, d.f. = 1,089). In contrast, for most neurons (64.75%), the variable subfield’s median frequency showed a positive correlation (median 0.09, one-tailed one-sample *t*-test against mean of 0, *t* = 16.23, *P* = 1.6 × 10^−53^, d.f. = 1,083). Four neurons were excluded from the fixed subfield analysis due to excessively small fixed subfield size. **e**, Parametric ‘CUB-grating’ dataset constructed from CUB segmentation masks, with object and background replaced by synthetic gratings. **f**, Using the CUB-grating dataset, we identified the most activating crop for each neuron. Simulated simple and complex cells predominantly preferred single grating images (83.3% and 75%, respectively). In contrast, V1 neurons exhibited a different pattern of preference (one-way chi-squared test, *χ*^2^ = 8,510, *P* < 10^−308^, and *χ*^2^ = 5,538, *P* < 10^−308^ for comparison against simulated simple and complex cells, respectively). While most simulated simple (83.3%) and complex (75%) cells preferred single grating images, V1 neurons almost exclusively preferred images with object boundaries (99.1%). V1 neurons showed preferences for boundaries defined by differences in spatial frequency alone (39.2%), orientation alone (21.6%), or a combination of both (38.3%). The marginal difference in preference was greater for spatial frequency than for orientation (*P* < 10^−4^, two-sided marginal difference bootstrapping). **g**, Top-100 activating crops from ‘high-frequency object’ images yielded higher mean matching scores than those from ‘low-frequency object’ images (two-sided Wilcoxon signed-rank test, *W* = 340,648, *P* = 2.0 × 10^−52^). Overall, 66.4% of neurons showed higher matching scores for ‘high-frequency object’ crops (same after BH correction), whereas 23.4% showed lower scores (23.3% after correction) (*P* < 0.05, two-sided Welch’s *t*-test, 170.2 average d.f.). **a-g**, Data were pooled from six mice, including 1,200 randomly selected neurons. Simulated simple and complex cells included 60 neurons each. Source data

To test this hypothesis, we utilized a natural image dataset with manual segmentation labels, Caltech-UCSD Birds-200-2011 (CUB)^25^. The CUB dataset is a comprehensive collection of 11,788 images spanning 200 bird species, each annotated with pixel-resolution segmentation masks for object and background. We screened over a million crops from the CUB dataset in silico, matching mean and root mean square (RMS) contrast to the MEI and VEIs, to identify highly activating crops for each V1 neuron (Fig. 5a). Across the population, highly activating crops were more likely to contain object boundaries than random crops (Supplementary Fig. 9c). To further quantify the alignment between the bipartite RF structure and the object boundaries in highly activating CUB crops, we computed a matching score between the segmentation label and the ‘bipartite mask’ defined by VEIs_partial_ (Fig. 5a and Methods). Highly activating image crops exhibited better alignment between bipartite subfield divisions and object boundaries than random crops, indicating a preferential response to object-background divisions (Supplementary Fig. 9a and Fig. 5b).

Next, we investigated which low-level visual statistics contribute to this alignment. Analysis of VEIs_partial_ revealed that most V1 neurons (76.5%) preferred spatial patterns with higher median frequency in the variable subfield compared to the fixed subfield (Fig. 5c and Methods), whereas simulated simple and complex cells showed no such bias when subjected to the same optimization procedure (Fig. 5c). Consistent with this pattern, natural image patches with higher-frequency content in the variable subfield tended to elicit stronger responses (64.8%), while patches with lower-frequency content in the fixed subfield were associated with stronger activation (79.1%) (Fig. 5d). These findings led us to hypothesize that V1 neurons are particularly sensitive to object boundaries defined by differences in spatial frequency.

To explicitly test this hypothesis, we created a modified CUB dataset (‘CUB-grating’) in which object and background regions were replaced with grating stimuli of varying spatial frequencies and orientations while preserving naturalistic boundaries, and presented crops from this dataset in silico (Fig. 5e). Our analysis revealed striking differences between simulated cells and V1 neurons. While most simulated simple (83.3%) and complex (75%) cells preferred single grating images, V1 neurons almost exclusively preferred images with object boundaries (99.1%) (Fig. 5f). Specifically, V1 neurons showed preferences for boundaries defined by differences in spatial frequency alone (39.2%), orientation alone (21.6%), or a combination of both (38.3%). Notably, the difference in preference was greater for spatial frequency than for orientation (Fig. 5f). Highly activating CUB-grating crops also showed strong alignment between their segmentation labels and bipartite masks (Fig. 5g), but in this case the variable and fixed subfields corresponded systematically to high- and low-frequency regions, respectively, rather than to object and background per se. These results generalized across different inclusion criteria used to identify patches containing object boundaries (Supplementary Fig. 10). Thus, our analysis revealed that mouse V1 neurons preferentially responded to object boundaries defined by frequency discontinuities, with the variable subfield favoring higher spatial frequency than the fixed subfield.

### The MICrONS dataset reveals synaptic connectivity reflecting a functional invariance hierarchy in V1 L2/3

To relate neuronal response invariances to synaptic-level cortical architecture, we leveraged the MICrONS functional connectomics dataset, which combines large-scale functional recordings with dense electron microscopy reconstructions of synaptic connectivity in mouse V1^8^. To quantify functional invariances, we employed a dynamic digital twin model of the MICrONS mouse that uses the foundation model from Wang et al.^9^ (Methods), which accurately predicted responses to various stimulus domains including natural movies, static images, and artificial parametric stimuli.

**Fig. 6:**
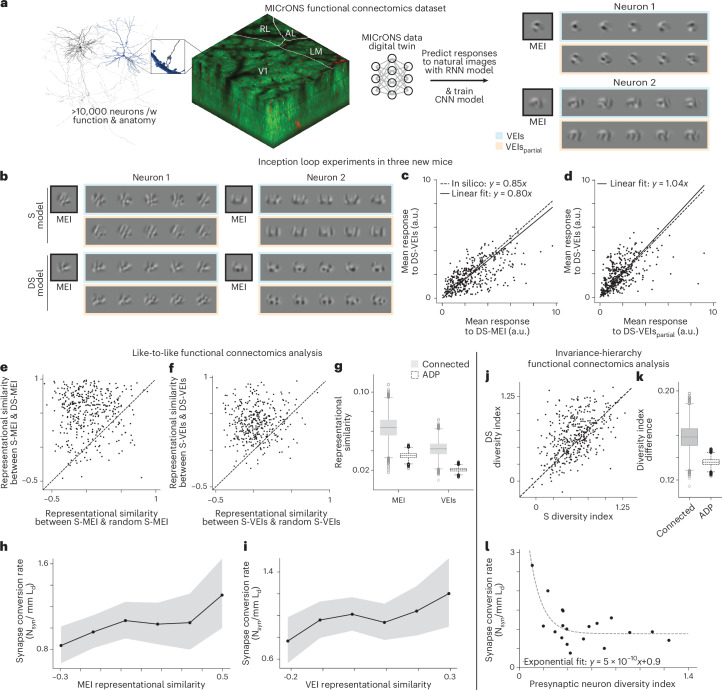
MICrONS functional connectomics analysis. **a**, Schematic of the MICrONS functional connectomics dataset^8^, comprising responses of >75,000 neurons to dynamic stimuli and their reconstructed subcellular connectivity from electron microscopy data. We employed the MICrONS ‘digital twin’^9^, trained on dynamic stimuli (denoted as a ‘dynamic’ model; recurrent neural network, RNN) to predict responses to natural images used in our experiments. A new CNN model was trained on these in silico predictions (‘dynamic-static’ or DS model) and used to synthesize MEIs, VEIs and VEIs_partial_. **b**, MEIs and VEIs optimized using our standard model (‘static’ or S model) and DS model for two example neurons. **c**, DS-VEIs stimulated neurons in vivo at 80 ± 3% of DS-MEI activation, close to the in silico prediction of 85% (two-sided Wilcoxon signed-rank test, *W* = 31,534, *P* = 2.8 × 10^−4^), with only 10.3% of all neurons showing different responses between VEIs and 85% of MEI (0.25% after BH correction) (*P* < 0.05, two-sided Welch’s *t*-test with 32.0 average d.f.). **d**, DS-VEIs_partial_ activated target neurons similarly to DS-VEIs (two-sided Wilcoxon signed-rank test, *W* = 29,878, *P* = 1.4 × 10^−5^) with only 9.5% of all neurons showing different responses (0.0% after BH correction) (*P* < 0.05, two-sided Welch’s *t*-test with 32.0 average d.f.). **e**, DS-MEIs were more similar to S-MEIs of the same neuron than S-MEIs of other random neurons (two-sided Wilcoxon signed-rank test, *W* = 4,537, *P* = 4.0 × 10^−53^). **f**, Similarly, DS-VEIs were more similar to S-VEIs of the same neuron than S-VEIs of other random neurons (two-sided Wilcoxon signed-rank test, *W* = 3,969, *P* = 8.8 × 10^−55^). **g**, The mean MEI and VEI similarities of connected pairs (0.06 ± 0.02 and 0.04 ± 0.02) were higher than those of the ADP control pairs^26^ (0.03 ± 0.01 and 0.021 ± 0.004; *P* = 0.02 and *P* < 10^−4^, respectively, two-sided bootstrapped mean difference after BH correction). **h**,**i**, Synapse conversion rate (N_syn_/mm L_d_ where N_syn_ denotes the number of synapses between two neurons and L_d_ denotes the axon-dendrite co-travel distance in mm) increased linearly with the MEI (**h**) and VEI (**i**) representational similarity for neuron pairs (*P* = 0.014 and 0.0034, respectively, two-sided *t*-test for linear coefficient against 0 using Poisson generalized linear mixed model with random intercepts). Neuron pairs were binned by their MEI and VEI similarity, respectively. Shaded areas represented 95% CIs from bootstrapping. **j**, Diversity indices from the DS model highly correlated with those from the S model (Pearson *r* = 0.46, *P* = 1.2 × 10^−22^, two-sided *t*-test). **k**, The mean diversity index increase for connected pairs was greater than that for ADP control pairs (0.16 ± 0.02 and 0.14 ± 0.01, respectively; *P* = 0.04, two-sided bootstrapped mean difference against 0 after BH correction). **l**, Presynaptic neurons with lower diversity indices showed higher synapse conversion rate (Spearman’s rank correlation coefficient *ρ* = − 0.49, *P* = 0.03, two-sided *t*-test). This relationship was well-modeled by an exponential decay (*R*^2^ = 0.58). **g**,**k**, Box plots show center line (median); box bounds (25th to 75th percentiles, IQR); whiskers extend to the most extreme data points within 1.5 × IQR of the quartiles; caps mark whisker ends; points beyond whiskers are plotted as outliers. **c–f**,**j**, Data for in vivo verification of the DS model were pooled over 399 neurons from three mice. **g–i**,**k**,**l**, Data for MICrONS functional connectomics analysis were pooled over 19 presynaptic neurons forming 706 connected pairs and 18,162 ADP controls. Source data

Before analyzing MICrONS, we validated that VEIs derived from the dynamic digital twin faithfully captured neuronal invariances. In three additional mice, we recorded responses to static natural images and to the same natural and parametric movie clips used in MICrONS, and for each animal trained two CNNs with the same architecture as in our main experiments: a ‘static’ (S) model fit directly to in vivo responses to static images, and a ‘dynamic-static’ (DS) model fit to in silico responses of the animal’s dynamic digital twin to the same static images (Fig. 6a). Both models accurately predicted held-out responses (Supplementary Fig. 11a), and MEIs, VEIs synthesized from the DS model were highly similar to those from the S model (Fig. 6b,e,f and Supplementary Fig. 11e). Diversity and BIIs were also strongly correlated between models (Pearson *r* = 0.46 and 0.66, respectively; Fig. 6j and Supplementary Fig. 11f). When presented back to the animals, DS-derived MEIs, VEIs, and VEIs_partial_ robustly activated their target neurons: VEIs reached 80 ± 3% of MEI responses in vivo, close to the predicted 85%, and VEIs_partial_ were as effective as VEIs (Fig. 6c,d and Supplementary Figs. 12–14). These results validate the use of dynamic digital twins to synthesize highly activating stimuli and to quantify neuronal invariances.

For MICrONS, we focused on V1 L2/3 excitatory neurons with matched electron microscopy reconstructions, high reliability and accurate digital twin predictions^26^. This yielded 19 presynaptic neurons and 570 postsynaptic partners, forming 706 synaptically connected pairs. A well-established principle in the functional connectomics domain is the like-to-like connectivity rule (excitatory neurons with similar response properties are more likely to form connections^26–28^). We re-examined this rule using MEI- and VEI-based similarities, leveraging the synaptic-level resolution of the MICrONS dataset. To control for anatomical opportunity, we compared synaptically connected pairs to axonal–dendritic proximity (ADP) control pairs, which share local axon–dendrite overlap but lack synapses^26,29^. Connected pairs showed higher MEI and VEI representational similarity than ADP controls (Fig. 6g), indicating that like-to-like connectivity is expressed at synaptic resolution rather than being simply a byproduct of broader spatial patterns of neuronal organization. Moreover, the synapse conversion rate (the number of synapses per unit axon–dendrite overlap) increased with MEI and VEI similarity (Fig. 6h,i), consistent with a higher likelihood of connection among functionally similar neurons^26,28^. These findings further corroborate the results reported by Ding et al.^26^, who demonstrated that the like-to-like connectivity rule in the feature domain operates at the synaptic level across different types of connections, both within and across cortical layers and areas.

We next investigated the relationship between neuronal invariance and circuit structure. Hierarchical models of the cortex have long speculated that complex functional invariance could arise from the convergence of excitatory presynaptic inputs with simpler invariances, as in Hubel and Wiesel’s account of complex-cell phase invariance arising from aligned simple cells^6,30,31^; however, evidence for this decades-old model has primarily relied on correlational analyses^32^, with direct evidence remaining elusive due to the challenge of simultaneously measuring both physiology and wiring of the same neurons.To test whether synaptic connectivity is associated with systematic differences in functional invariance, we compared synaptically connected neuron pairs with ADP controls. We found that connected pairs exhibited greater increases in diversity index than ADP controls (Fig. 6k), suggesting that the increase in functional invariance occurs at the synaptic level. Notably, there was no difference between the mean diversity indices of postsynaptic partners and ADP controls (Supplementary Fig. 15). Furthermore, we found that the synapse conversion rate decreased exponentially as the presynaptic neuron’s diversity index increased (Fig. 6l), implying that excitatory neurons with lower functional invariance are more likely to form intralaminar connections in V1 L2/3. Collectively, these findings provide evidence for a hierarchical organization among excitatory neurons in mouse V1 L2/3 that enhances single-neuron functional invariance.

## Discussion

Invariant object recognition is central to visual perception. In the object manifold disentanglement framework, each object corresponds to a continuous manifold generated by natural transformations such as translation, rotation, scaling and lighting changes^1^. In pixel space these manifolds overlap, and visual processing progressively separates them through hierarchical stages, enabling linear readout of object identity in higher areas. Single neurons in higher visual cortex are thought to integrate simpler feature detectors from earlier stages to build invariances to these transformations^6,33,34,1,31,30^^,53^, a principle that inspired convolutional neural network architectures^35^.

Despite this conceptual framework, systematic characterization of single-neuron invariances has remained difficult. The stimulus space is vast, neuronal computations are nonlinear, and experimental time is limited, so most classical examples come either from simple parametric stimuli in early areas^6,36^ or from semantically meaningful stimuli in higher areas^2–5,37^. Because both approaches sample only narrow regions of the stimulus manifold, there is no systematic methodology for characterizing neuronal invariances across the visual hierarchy. Recent advances in building digital twins of the brain and using nonparametric deep learning-based image synthesis have opened new avenues for finding the preferred stimuli of visual neurons^7,10,38^, but have largely emphasized selectivity rather than explicitly studying neuronal invariance.

Here we extend this framework to the invariance problem by synthesizing VEIs for individual neurons in mouse V1 L2/3. These stimuli reveal a new bipartite invariance that goes beyond the classical phase invariance in complex cells^6^. In this organization, one RF subfield prefers a fixed spatial pattern, whereas the other prefers random crops from a texture image. While previous work suggested a bimodal distribution of phase invariance for simple and complex cells^36^, bipartite invariance in mouse V1 L2/3 cannot be explained as a continuum of, or mixture between, these classical models. A null model that parametrizes VEIs as weighted sums of two fully overlapping subfields fails to produce VEIs that are as diverse or strongly activating as bipartite VEIs, indicating that bipartite structure is required. Moreover, shift invariance resides primarily in the variable subfield, as introducing it into the fixed subfield reduces responses.

Additionally, we show that the bipartite structure cannot be explained by classical center-surround interactions, consistent with findings from Fu et al.^39^, which demonstrated that MEIs correspond well to classical RF measurements, while extra-classical surround modulation extends far beyond the MEI. In particular, we observed no consistent spatial relationship between the minimum response field (MRF) and either the fixed or variable subfields, further ruling out center-surround mechanisms as an explanation for bipartite invariance.

The concept of invariance in neuronal responses can be defined in various ways, each with its own merits and implications. In our study, we define invariance as input transformations that preserve response magnitude (a definition particularly relevant for early visual areas such as V1, where information is represented across dense populations of broadly responsive neurons)^20,40^; however, to generalize our procedure to higher visual areas, an alternative definition of invariance may be necessary. For instance, in primate inferotemporal cortex (IT), it has been widely reported that while neurons do not preserve their response magnitude to the same objects of different sizes or placed in different positions, they typically maintain their rank-order object preference within their RFs^41,42^.

While we have focused primarily on shift invariance, it is unlikely to be the only type of invariance existing in mouse vision. As an initial effort to parameterize new empirical invariances, it is also worth acknowledging that our partial-texture model proposes a simple hypothesis of a binary division of the presence and absence of shift invariance in the RF without considering more complicated scenarios such as nonlinear cross-subfield interactions. We also acknowledge that parameterizing complex invariances (for example, 3D pose) for higher visual areas remains challenging. Future studies using photorealistic rendering engines with explicitly defined latent variables and image transformations will allow for a more generalized parameterization of invariances in a well-defined latent space, including 3D pose and other complex transformations. Nonetheless, we believe the new bipartite invariance can be of great use as a computational principle for future designs of biologically plausible or brain-inspired computer vision systems^43^ and serve as an empirical test for theoretically driven^44,45^ or data-driven models^14,46,47^ that aim to explain and predict neuronal responses in the visual system.

The two RF subfields of the bipartite structure exhibit distinct characteristics, differing in both level of invariance and preferred spatial frequency. This property closely parallels ‘high-low-frequency detectors’ observed in artificial neural networks, which detect low-frequency patterns on one side of their RF and high-frequency patterns on the other^48^, suggesting that bipartite invariance with frequency bias may be a common feature shared between biological and artificial visual systems for boundary detection. Classical simple and complex cells are strongly activated by luminance-defined edges^6,24,49^, whereas V1 bipartite neurons are biased toward boundaries constructed by second-order cues such as spatial frequency variation.

Our findings further complement behavioral studies showing that mice are able to use texture-based cues for segmentation^50,51^. While previous research emphasized boundaries constructed by orientation or phase differences^51^, our results indicate that spatial frequency variation could provide an additional visual cue for boundary detection in mouse vision. Notably, humans also use spatial frequency as a cue for object-background assignment, often perceiving higher-frequency regions as objects^52^. This preference mirrors that of V1 neurons, suggesting potential common strategies for object-background segmentation between mice and primates.

The brain’s ability to generalize has long been hypothesized to rely on a cortical hierarchy where neurons tuned to simpler features combine to build complex functional invariance^1^^,30^^,^^31^^,34^^,53^^,33^^,6^. This concept originates from Hubel and Wiesel’s model of complex cells achieving phase invariance by integrating inputs from simple cells^6^; however, empirical validation has been challenging due to the difficulty of simultaneously measuring physiology and wiring at the single-cell level^54^ and accurately modeling and quantifying functional invariance^42,53^. Our study overcomes these challenges by utilizing the MICrONS dataset, the largest functionally imaged electron microscopy dataset to date^8^, and a digital twin model from a state-of-the-art foundation model for mouse visual cortex^9^. We uncovered two key findings supporting hierarchical organization within V1 L2/3:

1. Postsynaptic neurons exhibit higher level of functional invariance than their presynaptic counterparts.
2. Lower invariance presynaptic neurons form exponentially more synapses per unit of axon–dendrite co-traveling distance.

These findings provide the first evidence of a functional invariance hierarchy at the individual neuron level within the same cortical area and layer, mediated by horizontal connections. This complements models such as HMAX^30,31,34^, which focused on hierarchies between cortical areas, reveals previously unrecognized computational flexibility, and aligns with studies demonstrating the importance of lateral connections for invariant object representation^55,56^.

As connectomics proofreading of the MICrONS volume progresses^8,29^, access to more completely reconstructed connectivity will enable finer-grained tests of how multiple presynaptic partners jointly shape bipartite properties of postsynaptic neurons. We also aim to extend our analysis to higher cortical areas to explore functional invariance across the visual processing hierarchy. Future studies using more sophisticated models or direct in vivo measurements could further validate and refine these findings, potentially uncovering additional insights into cortical processing organization. Moreover, it would be important to compare how our findings generalize to other species, such as nonhuman primates, where there are some similarities but also important differences in the functional organization of V1.

Overall, our work represents an important advance in understanding cortical processing and neuronal tuning by combining large-scale neuronal recordings with advanced deep neural network techniques to systematically characterize single-neuron invariances. The discovery of bipartite invariance in mouse V1 challenges long-held assumptions about RF homogeneity and offers new insights into natural image segmentation. By leveraging the MICrONS dataset, we also provide the first empirical evidence for a functional invariance hierarchy within V1 L2/3, validating and extending theoretical models of cortical organization. The flexibility of our paradigm opens up possibilities for exploring neuronal invariances across various cortical regions, sensory modalities and species, promising to illuminate the complex nature of neuronal coding more broadly, and potentially informing the development of more sophisticated, biologically plausible artificial intelligence systems.

## Methods

### Neurophysiological experiments

#### Two-photon calcium imaging

The following procedures were approved by the Institutional Animal Care and Use Committee of Baylor College of Medicine. Animals were housed in a controlled environment (20–22^°^C, 30–70% humidity) on a 12-h light–dark cycle, and all experiments were conducted during the subjective night. A total of 17 mice (*Mus* *musculus*: 9 male, 8 female) aged 6–17 weeks, expressing GCaMP6s in excitatory neurons via Slc17a7-Cre and Ai162 transgenic lines (stock nos. 023527 and 031562, respectively; The Jackson Laboratory) were selected for experiments. The mice were anesthetized and a 4-mm craniotomy was made over the visual cortex of the right hemisphere as described previously^20,58^. For functional imaging, mice were head-mounted above a cylindrical treadmill and calcium imaging was performed using a Chameleon Ti-Sapphire laser (Coherent) tuned to 920 nm and a large field-of-view mesoscope equipped with a custom objective (0.6 numerical aperture, 21-mm focal length)^59^. Laser power at the cortical surface was kept between 13.18 mW and 21.96 mW and maximum laser output of 61 mW was used at 245 μm from the surface.

We also recorded the rostro-caudal treadmill movement as well as the pupil dilation and movement. The treadmill movement was measured via a rotary optical encoder with a resolution of 8,000 pulses per revolution and was recorded at approximately 100 Hz to extract locomotion velocity. Light diffusing from the laser during scanning through the pupil was used to capture pupil diameter and eye movements. The images of the left eye were reflected through a hot mirror and captured with a GigE CMOS camera (Genie Nano C1920M; Teledyne Dalsa) at 20 fps with a resolution of 246–384 pixels × 299–488 pixels. A DeepLabCut model^60^ was trained on 17 manually labeled samples from 11 animals to label each frame of the compressed eye video with eight eyelid points and eight pupil points at cardinal and intercardinal positions. Pupil points with high likelihood were fitted with the smallest enclosing circle, and the radius and center of this circle were extracted.

We delineated visual areas by manually annotating the retinotopic map generated by pixel-wise response to a drifting bar stimulus across a 4,000 × 3,600 μm^2^ region of interest (ROI) (0.2 px μm^−1^) at 200 μm depth from the cortical surface. The imaging site in V1 was chosen to minimize blood vessel occlusion and maximize stability. Imaging was performed using a remote objective to sequentially collect ten 630 × 630 μm^2^ fields per frame at 0.4 px μm^−1^ *xy* resolution at approximately 8 Hz for all scans. We allowed only 5-μm spacing across depths to achieve dense imaging coverage of a 630 × 630 × 45 μm^3^ *xyz* volume. The most superficial plane positioned in L2/3 was around 200 μm from the surface of the cortex. Thanks to our dense sampling, cells in the imaged volume were heavily over-sampled, often appearing in at least two or more imaging planes. This allowed matching across days with 2.5 ± 2.6 μm vertical distance between masks (see details below). We performed raster and motion correction on the imaging data and then deployed the CNMF algorithm^61^ implemented by the CaImAn pipeline^62^ to segment and deconvolve the raw fluorescence traces. Additionally, cells were selected by a classifier^62^ trained to detect somata based on the segmented cell masks and resulted in 7,049–8,238 soma masks per scan. The full two-photon imaging processing pipeline is available at (https://github.com/cajal/pipeline).

We did not employ any statistical methods to predetermine sample sizes but our sample sizes are similar to those reported in previous publications. Data collection and analysis were not performed blind to the conditions of the experiments but no animal or collected data point was excluded for any analysis performed unless explicitly stated.

#### Electrophysiological recording

Six mice (*M.* *musculus*; two male and four female) aged 14–27 weeks were selected for experiments, with two females expressing GCaMP6s in excitatory neurons via Slc17a7-Cre and Ai162 transgenic lines (stock nos. 023527 and 031562, respectively; The Jackson Laboratory) and the rest being C57BL/6J wild-type (stock no. 000664; The Jackson Laboratory). We performed acute recordings using Neuropixels probes 1.0 in awake, head-fixed mice as described previously^22^. In brief, animals were implanted with a headpost and habituated to the experimental setup (head fixation on a treadmill) after recovery. On the recording day, the animals were briefly anesthetized with isoflurane and a 1-mm craniotomy was made above visual cortex (approximately 2.9 mm lateral to the midline sagittal suture and anterior to the lambda suture)^20^. The animals were then transferred to the experimental setup and allowed to recover from anesthesia. Location of probe insertion was chosen according to stereotaxic coordinates for targeting V1 using Pinpoint^63^, with all penetrations ranging from 600 μm to 1,100 μm on the anteroposterior axis, 2,900 μm to 3,500 μm on the mediolateral axis, and at an angle of 55^∘^ or 60^∘^ with respect to the ventrodorsal axis. One probe was smoothly lowered through the craniotomy to the final depth according to the trajectory planning with Pinpoint^63^ to cover the whole cortex (covering 1,800−2,000 μm of the probe) and allowed to settle for approximately 20 min before any recording. Visual area segmentation was performed by mapping the reversals of the retinotopy based on the RF progression along the probe as described previously^64^. Neuronal activity recordings were made with custom-written software in LabView and then automatically spike sorted with the Kilosort3 spike-sorting software^65^. An external infrared light was used as the light source for capturing pupil diameter and eye movements. A DeepLabCut model^60^ was trained on 13 manually labeled samples from 4 animals to label each frame of the compressed eye video with eight eyelid points and eight pupil points at cardinal and intercardinal positions. Pupil location and radius were extracted following the identical procedure described in ‘Two-photon calcium imaging’. From a total of nine recording sessions, 3,283 neurons were detected by the spike-sorting algorithm (136–547 per session), with 364 neurons from V1 L2/3 (12–95 per session). All V1 L2/3 neurons were compiled together for predictive model training, and then neurons classified as ‘single units’ or ‘multi-unit activity’ were used separately for downstream analysis. We evaluated the level of unit contamination using ISI violations, following the approach introduced by Hill et al.^23^. This metric represents the relative firing rate of hypothetical contaminating sources that produce these violations, with higher ISI violations indicating greater level of contamination.

#### Visual stimuli presentation

Visual stimuli were presented 15 cm away from the left eye with a 25′ LCD monitor (31.8 × 56.5 cm, ASUS PB258Q) at a resolution 1,080 × 1,920 pixels and refresh rate of 60 Hz. We positioned the monitor so that it was centered on and perpendicular to the surface of the eye at the closest point, corresponding to a visual angle of 2.2^°^ per cm on the monitor. To estimate the luminance level of the stimuli presented on the monitor, we taped a photodiode at the top left corner of the monitor and recorded its voltage during stimulus presentation, which is approximated linearly correlated with the monitor luminance. The conversion between photodiode voltage and luminance was estimated from luminance measurements from a luminance meter (LS-100 Konica Minolta) for 16 equidistant pixel values ranging from 0 to 255 while simultaneously recording the photodiode voltage. As the relationship between photodiode voltage and luminance is usually stable, we only perform such measurements every few months. In the beginning of every experimental session, we computed the gamma between pixel intensity and photodiode voltage by measuring photodiode voltage at 52 equidistant pixel values ranging from 0 to 255; then we further interpolated the corresponding luminance at each pixel intensity. For closed-loop experiments, the pixel-luminance interpolation computed on day 1 was used throughout the loop. All stimuli used in the current study were presented at gamma value ranging from 1.59 to 1.77 and monitor luminance ranging from 0.07 ± 0.16 cd m^−^^2^ to 9.58 ± 0.65 cd m^−2^.

#### Presentation of natural stimuli

To fit neurons’ responses, 5,100 natural images from ImageNet (ILSVRC2012) were cropped to fit a 16:9 monitor aspect ratio and converted to grayscale. To collect data for training a predictive model of the brain, we showed 5,000 unique images as well as 100 additional images repeated ten times each. This set of 100 images were shown in every scan for evaluating cell response reliability within and between scans. Each image was presented on the monitor for 500 ms followed by a blank screen lasting between 300 and 500 ms, sampled uniformly. Identical natural stimuli were used for two-photon imaging and electrophysiological experiments. To maintain the animal’s alertness throughout each scan, we interspersed an additional set of six brief video clips at regular intervals.

### Neuronal data processing and predictive modeling

#### Preprocessing of neuronal responses and behavioral data

Neuronal responses were deconvolved using constrained nonnegative calcium deconvolution and then accumulated between 50 and 550 ms after stimulus onset of each trial using a Hamming window^61^. All the segmented neuronal masks from each individual scan were used for model training, including duplicates resulting from dense imaging. The corresponding pupil movement and treadmill velocity for each trial were also extracted and integrated using the same Hamming window. Each dataset consists of 4,500 and 500 unique images for training and validation, respectively; an additional set of 100 images presented with ten repeats was used for model evaluation. The original stimuli presented to the animals were isotropically downsampled to 64 × 36 pixels for model training. For day-1 model training scans, input images, neuronal responses and behavioral traces were normalized (*z*-scored for input images and divided by standard deviationfor the rest) across the training set during model training and evaluation. Trials with invalid behavioral data (0.8 ± 1.2%) were excluded from model training. For closed-loop verification scans, neuronal responses and behavioral traces were normalized across all trials.

#### Predictive model architecture and model training

We followed the same network architecture and training procedure as described previously^7,57^. Each model comprises: a shared nonlinear core (157,920 parameters), neuron-specific linear readouts at six different spatial scales (579 parameters per neuron), a behavioral modulator (150 shared parameters across all neurons and 84, 007 parameters per neuron), and a pupil position shifter network shared across all neurons (57 parameters). The common core is a three-layer CNN with full skip connections. Each layer contains a convolutional layer with no bias, followed by batch normalization, and an exponential linear unit (ELU) nonlinearity. The readout models the neuronal response as an affine function of the core outputs followed by ELU nonlinearity and an offset of 1 to guarantee positiveness. Additionally, we model the location of a neuron’s RF with a spatial transformer layer reading from a single grid point that extracts the feature vector from the same location at different scales of the downsampled feature outputs. The modulator computes a gain factor for each neuron that simply scales the output of the readout layer using a two-layer fully connected multilayer perceptron (MLP) with rectified linear unit nonlinearity and a shifted exponential nonlinearity to ensure positive outputs. Finally, because training mice to fixate their gaze is impractical, we estimated the trial-by-trial RF displacement shared across all neurons using a shifter network composed of a three-layer MLP with a tanh nonlinearity. For all model training, we adhered to the methodology outlined in Walker et al.^7^, training four instances of the same network with different initializations by minimizing the Poisson loss $\frac{1}{m}{\sum }_{i=1}^{m}\left({\widehat{r}}^{(i)}-{r}^{(i)}\log {\widehat{r}}^{(i)}\right)$ where *m* denotes the number of neurons, $\widehat{r}$ the predicted neuronal response and *r* the observed response.

#### Evaluation of model performance and neuronal reliability

Predictions from all four models are averaged for model benchmarking and image generation. We computed the model performance CC_*abs*_ for each neuron on the same held-out data as the correlation between the model-predicted response $\overline{x}$ and the recorded responses $\overline{y}$ averaged across ten repetitions:

1 $${\mathrm{CC}}_{\mathrm{abs}}=\frac{Cov(\overline{x},\overline{y})}{\sqrt{Var(\overline{x})Var(\overline{y})}}.$$

To assess reliability of neuronal responses, we computed CC_max_^12^ as

2 $${\mathrm{CC}}_{\max }=\sqrt{\frac{NVar(\overline{y})-\overline{Var(y)}}{(N-1)Var(\overline{y})}},$$

where *y* is the in vivo responses and *N* is the number of trials. This metric captures the consistency of neuronal responses to identical visual stimuli in held-out data, serving as an upper bound for our model’s potential performance. We then estimated the normalized correlation coefficient (CC_norm_)^12^ as the fraction of variation in neuronal responses to identical stimuli accounted for by the model prediction:

3 $${\mathrm{CC}}_{\mathrm{norm}}=\frac{{\mathrm{CC}}_{\mathrm{abs}}}{{\mathrm{CC}}_{\max }}.$$

### Nonparametric synthesis of optimal stimuli and controls

#### Neuron selection

This section describes neuron selection for stimulus synthesis for 14 of 17 mice used for all experiments except for DS model validation. We first excluded neuronal masks within 10 μm from the edge of the imaging volume, and then ranked the remaining masks based on descending model predictive accuracy. To avoid duplicated neurons, we started from the lowest-ranked neuron and iteratively added neurons such that they are at least 25 μm apart and have functional correlation <0.4 with all neurons selected. This filtering left us with 2,081–2,676 unique neurons for each scan. We restricted all analyses to neurons that exhibit reasonable levels of response reliability as well as model predictive accuracy. We evaluated neuronal reliability using ‘oracle score’^7^ (a metric highly correlated with CC_max_^12^, Pearson *r* = 0.9) for each neuron by correlating its leave-one-out mean response with that of the remaining trial across 100 images in the held-out test set. For synthetic stimulus generation, we applied hard thresholds on oracle score and model test correlation to include 19.9% of the population for mouse 1 and 79.0 ± 0.5% of the population for mice 2–14.

#### Generation of MEI

For each individual neuron, we adapted the activation maximization procedure described by Walker et al.^7^ to find the stimulus that optimally drive each individual neuron. Starting with Gaussian white noise, we iteratively refined the image by adding the gradient of the target neuron’s predicted response using an SGD optimizer with learning rate of 1.0 for 1,000 iterations. To mitigate high-frequency artifacts in image synthesis, we applied a Gaussian filter (*σ* = 1.0) to smoothen the gradient at every optimization step. To determine the appropriate RMS contrast value for our synthetic stimuli, we conducted a pilot analysis in which we aggregated MEI masks from thousands of neurons into an average mask and measured mean contrast within this average mask across all the training set natural images presented. To prevent saturation and ensure that the synthetic stimuli remain within the well-trained contrast domain of the natural images used during model training, we standardized the image to a fixed mean of 0 and RMS contrast of 0.25 (the value obtained from the pilot analysis) following each gradient ascent step.

We computed a weighted mask for each MEI to capture the region containing the majority of the variance in the MEI image. We computed a pixel-wise *z*-score on the MEI and thresholded at *z*-score >1.5 to identify the highly contributing pixels. Then we closed small holes/gaps using binary closing, searched for the largest connected region to create a binary mask *M* where *M* = 1 if the pixel is in the largest region identified. Then, a convex hull was calculated using the identified pixels. Last, to avoid edge artifacts, we smoothed out the mask using a Gaussian filter with *σ* = 1.5 to avoid potential edge effects.

#### Generation of VEIs

We modified procedures described previously^16^ to optimize VEIs. For each individual neuron, we synthesized a set of images initiating from MEI that preserve high activation while differing as much as possible from each other. To optimize this set, we initiated from 20 instances of the target neuron’s MEI with different additive Gaussian white noises MEI + *σ*_*i*_ = *I*_*i*_ where 1 ≤ *i* ≤ 20 and iteratively minimize the loss:

4 $$L=\frac{1}{n}\mathop{\sum }\limits_{i=1}^{n}\max \left(c-\frac{{r}_{i}}{{r}_{MEI}},0\right)-\lambda \mathop{\min }\limits_{i,\,j}d({I}_{i},{I}_{j}),$$

where *r*_*i*_ and *r*_MEI_ are the model-predicted response to VEI_*i*_ and MEI, *c* is the minimum activation relative to *r*_MEI_ that we target each VEIs for, *d*(*I*_*i*_, *I*_*j*_) is the Euclidean distance in pixel space between VEI_*i*_ and VEI_*j*_ measured within the MEI mask (the neuron’s RF). The first term encourages all VEIs to achieve high activation, while the second term maximizes the minimum pairwise distance among VEIs. Specifically, we required each VEI to evoke at least 85% of their corresponding MEI (*c* = 0.85). This threshold was selected based on the previous finding that additional decrease in target response leads to marginal gain in minimum pairwise distance among VEIs for simulated complex cells^16^. Of note, the minimum, instead of average distance, was used in the second term to avoid solutions that form the set of VEIs into clusters by pushing apart the most similar pair of VEIs at every iteration. We employed the same gradient blurring and post-gradient image standardization as in MEI optimization. We optimized the VEI set for 3,000 iterations with a learning rate of 1,000 for the first 2,000 iterations and decayed to 100 for the last 1,000 iterations. This learning rate decay helped to further mitigate the occurrence of high-frequency artifacts. We performed the optimization for every target neuron with a series of diversity regularization hyper-parameter *λ*, densely sampled from 1 × 10^−4^ to 5 × 10^−2^. For each neuron, the set optimized using the largest *λ* that preserved minimal response greater than 85% of the MEI response was selected as the VEIs and used for downstream analyses and experiments.

#### Diversity index

To quantify the diversity level of each set of VEIs we derived a diversity index based on the average pairwise Euclidean distance of the VEIs. To position this metric on a meaningful spectrum with interpretable reference points, we estimated diversity levels of idealized simple and complex cells (see details in ‘Simulation of simple and complex cells’). Particularly, we estimated the lower/upper bounds (*d*_lower_ and *d*_upper_) as the median average pairwise Euclidean distance of VEIs from a population of noiseless simple/complex cells, respectively. We performed an exhaustive search through the Gabor parameter space to identify their VEIs. When standardized with a fixed mean and RMS contrast, VEIs from idealized simple cells have the same average pairwise Euclidean distance regardless of the underlying Gabor parameters. Similarly, idealized complex cells with different Gabor parameters have identical yet higher average pairwise Euclidean distance. For each real neuron, a diversity index (*D*) is calculated for each mouse V1 neuron *i* based on the average pairwise Euclidean distance of its VEIs *d*^(*i*)^ as

5 $${D}^{(i)}=\frac{{d}^{(i)}-{d}_{{\rm{lower}}}}{{d}_{{\rm{upper}}}-{d}_{{\rm{lower}}}}.$$

#### Natural image and synthesized controls for the invariance manifold

To evaluate the specificity of the invariance manifold represented by the VEIs, we designed two types of control stimuli: natural image controls and synthesized controls. Both controls were strictly closer to the MEI than all the VEIs, as quantified by the corresponding metric used in VEI generation. For each neuron, we first computed the minimum distance from the VEIs to the MEI within the MEI mask, denoted as *d*_target_, which served as the distance budget for control image selection or synthesis. For natural image controls, we searched through more than 40 million of natural image patches to identify those with distances from the MEI between 80% and 100% of *d*_target_. The synthesized controls were generated using a modified version of our VEI synthesis objective (equation (4)), where the first term aimed to match the distance from control images to the MEI to *d*_target_ rather than encouraging high activation:

6 $$L=\frac{1}{n}\mathop{\sum }\limits_{i=1}^{n}\left({d}_{\mathrm{target}}-d({I}_{i},\mathrm{MEI})\right)-\lambda \mathop{\min }\limits_{i,\,j}d\left({I}_{i},{I}_{j}\right).$$

For both control types, we created 20 different images per neuron and presented each once in vivo during closed-loop experiments.

#### Selection of natural VEIs

For each neuron, we searched through 41 million ImageNet image patches in silico to identify natural crops that elicited activations equal to or greater than 85% of the MEI response (VEI-like activation). To mitigate the effect of contrast difference at the edges of masked natural crops and MEIs, we refined MEI masks by shrinking them until the activation of masked MEI dropped below 95% of the original MEI activation, following the approach of Walker et al.^7^. Each crop was then masked using the refined MEI mask, and its mean and RMS contrast were adjusted to match those of the MEI. For neurons with at least 20 highly activating crops, we selected 20 natural VEIs (matching the number of synthesized VEIs per neuron) by greedily maximizing their minimum pairwise distance, mirroring the VEI synthesis procedure. These selected images are denoted as ‘natural VEIs’.

#### Generalization of VEIs

To test the generalizability of our VEI synthesis methodology, we modified key components of the synthesis pipeline and compared the resultant VEIs:

1. Image initialization: VEIs were initiated with full-field random white noise instead of a combination of the MEIs and random white noises.
2. Model initialization: The in silico model ensemble was trained from scratch using a different random initialization seed.
3. Individual model synthesis: VEIs were generated using the response of a single model from the ensemble rather than the average response of four models.
4. Diversity metric: VEIs were synthesized with diversity measured in neuronal representational space instead of pixel space, as detailed in ‘Generation of VEIs in neuronal representational space’.
5. Synthesis methodology: VEIs were generated using an alternative approach described in ‘Generation of VEIs with implicit neural representation model and contrastive regularization’.
6. Model architecture: VEIs were produced using the distinct model architecture outlined in Willeke et al.^14^.

We computed representational similarity (as detailed in ‘Representational similarity’) and average pairwise Euclidean distance of VEIs generated from various conditions to assess the robustness of VEI generation.

#### Generation of VEIs in neuronal representational space

We utilized the same loss function as in equation (4) but quantified pairwise VEI diversity *d*(VEI_*i*_, VEI_*j*_) as the negative Pearson correlation between model-predicted neuronal population response vectors *r*_*i*_ and *r*_*j*_ to VEI_*i*_ and VEI_*j*_:

7 $$d({\mathrm{VEI}}_{i},{\mathrm{VEI}}_{j})=-\frac{\sum ({r}_{i}-{\mu }_{i})({r}_{j}-{\mu }_{j})}{\sqrt{\sum {({r}_{i}-{\mu }_{i})}^{2}\sum {({r}_{j}-{\mu }_{j})}^{2}}},$$

where *μ*_*i*_, *μ*_*i*_ represent the mean neuronal population responses. To compute these population responses, we aligned all neurons’ RF centers with that of the target neuron for which the VEIs were being optimized. We refer to the VEIs generated through this method as ‘neuronal-space VEIs’.

#### Generation of VEIs from an implicit neural representation model with contrastive regularization

Following the approach detailed in Baroni et al.^21^, we used an implicit neural representation model (INRM) to map from a low-dimensional periodic latent space (one-dimensional (1D) or two-dimensional (2D)) to a manifold in image space representing the invariant transformations of a given neuron. The INRM we used was a fully connected feed-forward neural network mapping from pixel coordinates and latent inputs to pixel values. Our model consists of four layers of 50 hidden nodes, followed by a hyperbolic tangent nonlinearity and a sigmoid function as final nonlinearity. We used positional encoding on both the latent space and the coordinate space. Each latent input could be mapped to one image and changing the latent input corresponded to moving along one invariant dimension of the neuron. The images were standardized to a fixed mean and RMS contrast and clipped between values corresponding to the black and white pixels on the monitor before being passed to the digital twin to get the predicted response.

During training of an INRM, a jittering grid of uniformly distanced points was sampled from the latent space and mapped into a set of images. The training objective was composed of one activation term that maximizes the activation of the generated images and one contrastive term that encourages diversity across images and ensures smooth transitions in image space when navigating the latent space. Specifically, the contrastive regularization term achieved this by encouraging images corresponding to nearby points in latent space to have high cosine similarity and those corresponding to distant points in latent space to have low cosine similarity. The contrastive regularization temperature^21^ was set to 0.3. The latent space grid size was 20 points in 1D and seven points in 2D per dimension. The neighboring radius, which determined close-by points in the latent space, was set to 10% of the grid in 1D and 20% of the grid in the 2D. We used an Adam optimizer with learning rate of 0.001 to optimize the INRM weights. After a minimum of 500 weight updates, the regularization strength was decreased by a factor of 0.8 every time the activity stopped increasing (initial strength of 2.0, one check every 50 steps with patience of 5). Training was stopped when the resultant images achieved an average response larger than 85% of the MEI response and a minimum response larger than 75% of the MEI response. To avoid image artifacts, gradients were Gaussian blurred (*σ* = 1.0) and contrastive regularization was applied only on pixels within the MEI mask.

This method learns a continuous manifold of stimuli. In the 1D case, we sampled 20 VEIs corresponding to uniformly distant points in latent space. In the 2D case, as different latent dimensions could learn transformations associated with different image diversity, we obtained 20 VEIs by starting from an initial set of images corresponding to randomly sampled points in latent space and then optimizing them to maximize the minimum pairwise distance.

### Bipartite parameterization of VEIs

#### Bipartite model

We proposed a texture generative model to produce texture-based VEIs composed of two complementary subfields as follows:

8 $$\mathrm{VEIs}={m}_{V}T+{m}_{F}\mathrm{MEI},$$

where the first term is the variable subfield randomly cropped from an optimized texture canvas *T* using a mask *m*_*V*_. The second term is a fixed subfield masked directly from the original MEI. This model could be reduced to a full-texture model to describe global shift invariance if the entire RF (the MEI mask *m*_MEI_) was used as *m*_*V*_. We generated the texture *T* following Cadena et al.^16^ by maximizing the average activation of randomly sampled crops from *T* using *m*_*V*_. We followed the same loss as in nonparametric VEIs generation (equation (4)) to jointly maximize the activation and diversity of VEIs with the same regularizations (Gaussian blurring on the gradient and learning rate decay) but in this case, VEIs were parameterized as in equation (8). The same post-gradient image standardization was applied on these parametric VEIs.

To ensure that *m*_*V*_ captures the region of the original nonparametric VEIs from which we observed the most diversity, we pre-computed a series of *m*_*V*_s by varying the threshold on the pixel-wise variance across the VEIs. Specifically, starting from the pixels with the largest variance across VEIs, we kept expanding the *m*_*V*_ by requesting increasing fraction of the total variance from 0.2 to 0.6 within the variable subfield. The complement to *m*_*V*_ within *m*_MEI_ was used as the fixed subfield mask (*m*_*F*_). In general, the average predicted activation of the texture-based VEIs decreased as the size of *m*_*V*_ increased. For each set of texture-based VEIs resultant from each pair of subfield masks, we computed the harmonic mean between normalized activation and diversity index as follows:

9 $${H}=2\frac{\overline{{r}}\overline{{d}_{V\mathrm{EIs}}}}{\overline{{r}}+\overline{{d}_{V\mathrm{EIs}}}},$$

where $\overline{{r}}$ is the average activation and $\overline{{d}_{{\rm{VEIs}}}}$ is the average pairwise Euclidean distance, normalized by the maximal corresponding value across all different sets of VEIs using the series of *M*_*V*_s, respectively. We denoted the set of texture-based VEIs with the maximum harmonic mean as ‘partial-texture VEIs’ (VEIs_partial_). The set of texture-based VEIs resultant from the full-texture model were denoted as ‘full-texture VEIs’ (VEIs_full_).

#### Bipartite invariance index

The bipartite invariance index (BII) was devised to summarize the extent of partial shift invariance exhibited by a neuron. Using the series of subfield masks and their corresponding texture-based VEIs as described above, we fitted a quadratic-smoothing spline to model the relationship between the in silico neuronal activation and the variable subfield size. To capture the full range of this relationship, we uniformly sampled the variable subfield size between 0 and 1 and evaluated the predicted response at each point using the fitted spline. Finally, we calculated the area under the curve (AUC) of these predicted responses across the range of subfield sizes. This AUC value serves as our BII, encapsulating the neuron’s response profile across various subfield sizes and thus providing a comprehensive measure of its degree of partial shift invariance.

#### Preferred spatial frequency of bipartite RF subfields

Due to challenges of direct frequency analysis on small image windows (subfield masks), we employed an indirect comparative approach using two sets of images: (1) the full-field VEIs_partial_ and (2) modified versions of the full-field VEIs_partial_ where the content within the fixed subfield was substituted by random crops. These crops were drawn from the same texture optimized for variable subfield but masked using the fixed subfield mask, and standardized to have the same mean and RMS contrast as the original fixed subfield content. Both sets of images maintain the identical bipartite structure, differing only in the spatial content within the fixed subfield mask, thus providing an indirect but equitable way to compare frequency preferences of content from the two subfields. For each set of images, we first computed the radial power spectrum using ten equally spaced bins;the resulting power spectra were then averaged to obtain the mean radial power spectrum, from which the median frequency was estimated using linear interpolation.

#### Necessity and specificity of two subfields in the bipartite RF

We masked out or swapped the content of either subfield to evaluate its necessity and specificity in eliciting higher neuronal responses, respectively. When masking out a subfield, we prioritized maintaining the pixel integrity of the remaining content by applying a binary mask and restricting smoothing to regions outside the complementary subfield. This approach left portions of the complementary subfield visible in the stimulus, likely leading to an underestimation of the masked-out subfield’s necessity. For the specificity assessment, we either replaced the fixed subfields with different random natural image crops or the variable subfields by random crops masked from different random neurons’ optimized textures.

### Controls for bipartite RF structure

#### Control parameterization: ‘two-variable-subfield VEIs’

To investigate whether the fixed subfield exhibits shift invariance and if VEIs can be better explained by a more complex model, we modified the bipartite model such that both subfields are treated as shift-invariant, described by:

10 $$\mathrm{VEIs}={m}_{V}{T}_{1}+({m}_{MEI}-{m}_{V}){T}_{2},$$

Here, the first term mirrors the bipartite parameterization, while the second term represents a second variable subfield, randomly cropped from a second optimized texture canvas *T*_2_. We followed the same procedure as in ‘Bipartite model’ to sample a series of *m*_*V*_ and optimized *T*_1_ for each *m*_*V*_. Then we used the complementary subfield mask within the MEI mask *m*_MEI_ − *m*_*V*_ to optimize for a second texture canvas *T*_2_. We then combined crops masked from each subfield’s preferred texture to get sets of texture-based VEIs and selected the set with the highest harmonic mean of diversity and in silico activation as the ‘two-variable-subfield VEIs’.

#### Control parameterization: ‘no-spatial-division VEIs’

To assess the necessity of spatial division between the two subfields in the bipartite model, we developed an alternative parameterization that represents VEIs as a weighted summation of two fully overlapping subfields spanning the entire RF (estimated as the MEI mask *m*_MEI_): a fixed component directly from the MEI and a variable component cropped from a synthesized full-field texture. This model was implemented as

11 $$\mathrm{VEIs}=(1-c){m}_{\mathrm{MEI}}T+c\mathrm{MEI},$$

where the hyper-parameter *c* regulates the ratio between the variable and fixed subfield contribution. We uniformly sampled *c* between 0 and 1, where 0 signifies an ideal complex cell and 1 an ideal simple cell. For each *c*, we optimized the texture *T* following the same procedure as described in ‘Bipartite model’. We then combined the two overlapping subfields to get sets of texture-based VEIs and selected the set with the highest harmonic mean of diversity and in silico activation as ‘no-spatial-division VEIs’. We also fit the in silico activation as a quadratic-smoothing spline of the average pairwise Euclidean distance for each neuron (diversity). The spline fit was utilized to interpolate the diversity of these texture-based VEIs when their mean in silico activation was matched to that of the nonparametric VEIs. Similarly, we interpolated the mean in silico activation of these texture-based VEIs when their diversity was matched to that of the nonparametric VEIs. The same fitting and interpolation was also carried out for the bipartite model, allowing a direct comparison of how well these two parameterizations captured the diversity and in silico activation of the nonparametric VEIs.

#### Replication of bipartite structure using electrophysiological data

For Neuropixels electrophysiological data, we employed two strategies: training models from scratch, or initializing with a core pretrained on two-photon imaging data and subsequently training the remaining components (including neuron-specific readouts, shifter and modulator components) using Neuropixels data. The latter approach, particularly beneficial due to the limited number of neurons available from each Neuropixels recording session, improved the median normalized correlation coefficient (CC_norm_) from 0.64 to 0.73. We then generated MEIs, VEIs, texture-based VEIs following the same protocol as applied on the two-photon imaging models. For comparison of diversity and BIIs between neurons from imaging and electrophysiological data, we applied identical functional thresholding (oracle score > 0.22 and model test correlation > 0.42, respectively, calculated as the median threshold from 14 mice used for two-photon closed-loop experiments) on both neuron populations to ensure fair comparison.

#### Comparison of bipartite RF structure and the minimum response field

To investigate the relationship between classical RFs estimated with the minimum response field (MRF)^39^ and the bipartite RF structure, we presented sparse noise stimuli^66^ before and after the natural image stimuli (detailed in ‘Presentation of natural stimuli’) in the same two-photon imaging scan. The stimuli comprised circular bright (pixel value = 255) and dark (pixel value = 0) dots, each spanning 7° in visual angle, presented against a gray background (pixel value = 128) on a 9 × 9 grid covering 40% of the monitor’s central area. Each dot was displayed for 250 ms per location with 16 repetitions (eight before and eight after the natural stimuli). For both bright and dark dots, we aggregated neuronal responses from 50 to 300 ms post-stimulus onset for each trial, creating separate ON and OFF maps.

We then applied one-way analysis of variance to these maps to identify neurons exhibiting significant spatial variation in their responses. The MRF was determined by aggregating ON and OFF maps, maximizing the averaged response per location, and fitting a 2D Gaussian. For quality control, we excluded neurons with extreme MRF sizes (bottom 5% and top 95%) and those with low signal-to-noise ratio (SNR), calculated as $\mathrm{SNR}(x)=\frac{\mu ({x}_{\mathrm{mask}=1})}{\sigma ({x}_{\mathrm{mask}=0})}$, where the mask was obtained by thresholding the fitted Gaussian at 0.3. To evaluate the spatial relationship between the MRF and the bipartite structure, we calculated (1) the average pairwise distance between the MRF and each subfield across all pixels; (2) the overlap between the MRF and each subfield normalized by the MRF size. To estimate the diameter of MEI, variable subfield, and MRF, we first binarized their masks (threshold = 0.3) and defined the diameter as the maximum pairwise distance between points along the mask boundary.

#### Evaluation of pupil position uncertainty

To evaluate whether the bipartite structure is related to uncertainty in the trial-by-trial pupil shifts predicted by the shifter network, we trained three additional model variants using subsets of trials stratified by pupil movement. (1) In the minimal-movement model, we removed trials with pupil distance from the mean position larger than three units in the eye camera coordinate system, which corresponded to approximately one-twentieth of the median MEI diameter (2.86° visual angle); the median percentage of trials retained was 33.1%. (2) In the small-movement model, we included trials within the bottom 50th percentile of pupil movement. (3) In the large-movement model, we included trials within the top 50th percentile of pupil movement. For each model we generated MEI, VEIs, and partial-texture VEIs for every neuron.

### In vivo closed-loop verification of synthesized stimuli

#### Neuron selection

This section describes neuron section for stimulus synthesis for 14 out of the 17 mice used for all experiments except for DS model validation. For nine out of 14 mice, we randomly selected neurons with relatively high level of invariance (detailed below); for the remaining five mice, we randomly selected neurons from all candidates that survived our oracle score and model performance criteria (see above). To remove the confounding effect of RF size on neurons’ invariance level, we fit a least squares regression from the MEI mask size to the diversity index computed from VEIs (see above) using 1,000 random neurons compiled across eight pilot datasets. For each neuron, the residual between the actual mean VEI pairwise Euclidean distance and the predicted distance from the MEI mask size was calculated as its diversity residual. This diversity residual served as an size-independent evaluation of neuron’s invariance level. For each of the nine mice, we randomly selected neurons from the top 50th percentile among all neurons with positive diversity residuals.

#### Presentation of synthetic stimuli

For all MEIs, VEIs, texture-based VEIs and control stimuli, we masked the stimuli with the MEI mask and standardized all masked stimuli such that they have fixed value of mean (3.09 cd m^*−*^^2^) and RMS contrast (0.25 cd m^−2^) in the luminance space with only small amount of deviation due to clipping within the 8-bit range. The fixed mean and contrast valued were chosen to approximate those of the training set while minimizing the amount of clipping when converting synthetic stimuli from *z*-score space to the 8-bit image space. All pixels outside of the MEI mask were set to 128, the same intensity as the blank screen in between consecutive trials. For each neuron, the MEI was presented 20 times. In two mice, ten randomly selected VEIs were each presented for 20 repeats, whereas in the remaining mice, each of the 20 VEIs or control stimuli for each neuron were presented once.

#### Monitor positioning across days

To optimize the monitor position for centered visual cortex stimulation, we mapped the aggregate RF of the scan field ROI using sparse noise stimuli consisting of bright (pixel value = 255) and dark (pixel value = 0) dots. We tiled the center of the screen in a 10 × 10 grid with single dots in random locations, with ten repetitions of 200 ms presentation at each location. The RF was then estimated by averaging the calcium trace of an approximately 150 × 150 μm^2^ window in the ROI from 0.5–1.5 s after stimulus onset across all repetitions of the stimulus for each location. The resulting 2D map was fitted with an elliptic 2D Gaussian to find a center. To keep a consistent monitor placement across all imaging sessions, we positioned the monitor such that the aggregate RF of ROI in the first session was placed at the center of the monitor and then fixed the monitor position across the subsequent sessions within a closed-loop experiment. An L-bracket on a six-dimensional arm was fitted to the corner of the monitor at its location in the first session and locked in position such that the monitor could be returned to the same position between scans and across imaging sessions.

#### Cell matching across days

To return to the same image site, the craniotomy window was leveled with regard to the objective with six d.f., five of which were locked between days. A structural 3D stack encompassing the volume was imaged at 0.8 × 0.8 × 1 px^3 ^μm^−3^ *xyz* resolution with 100 repeats. The stack contained two volumes each with 150 fields spanning from 50 μm above the most superficial scanning field to 50 μm below the deepest scanning field; each field was 500 × 800 μm^2^, together tiling a 800 × 800 μm^2^ field of view (300 μm overlapped). This was used to register the scan average image into a shared *xyz* frame of reference between scans across days. To match cells across imaging scans, each two-dimensional scanning plane was registered to the 3D stack through an affine transformation (with nine d.f.) to maximize the correlation between the average recorded plane and the extracted plan from the stack. Based on its estimated coordinates in the stack, each cell was matched to its closest cell across scans. To further evaluate the functional stability of neurons across scans, in each scan we included an identical set of 100 natural images with each repeated ten times (referred as oracle images). For each pair of matched neurons from two different scans, we compute the correlation between their average trial responses to the oracle images. To be included for downstream analyses, the matched cell pair need to (1) have an intercellular distance smaller than 10 μm; (2) achieve a functional correlation equal to or greater than the top 1 percentile of correlation distribution between all unmatched cell pairs (estimated as 0.42); and (3) survive manual curation of the matched pair’s physical appearance in the processed average frame. Among all closed-loop scans, 56 ± 16% of closed-loop neurons per scan survived all three criteria.

### Analysis of in vivo neuronal responses

#### In vivo response comparisons and statistical analysis

Recorded responses were normalized across all presented images within each scan. For matched neurons, we averaged responses across either 20 repetitions of a single image (for MEIs and individual VEIs) or single presentations of 20 different images (for VEIs, texture-based VEIs and control stimuli). To assess the statistical significance of response differences for individual neurons, we employed two-sided Welch’s *t*-tests. For evaluating the overall difference in average responses across all neurons, we utilized two-sided Wilcoxon signed-rank tests.

#### Individual VEI response analysis

To compare the in vivo responses of individual VEIs to their corresponding MEI, we randomly selected ten VEIs for each neuron and presented each VEI 20 times. Using a two-sided Welch’s *t*-test, we assessed whether responses to individual VEIs differed from 85% of their corresponding MEI response. To determine whether our experimental procedure can reliably detect reductions in neuronal responses relative to the MEI, we analyzed the empirical probability of observing lower activation levels given our sample size. For each predefined activation level below 85%, we generated two sets of 20 MEI trials per neuron by sampling with replacement: a reference set scaled to 85% and a comparison set scaled to the selected activation level. We then applied a one-tailed Welch’s *t*-test to assess whether the comparison set exhibited lower activation. Repeating this procedure across all neurons tested in closed-loop experiments allowed us to quantify the relationship between response reductions compared to MEI activation in vivo and the fraction of significant tests (statistical power; Extended Data Fig. 2b). Finally, the proportion of individual VEIs eliciting responses below 85% of their corresponding MEI was used as a proxy for statistical power to estimate the 95% confidence intervals of the normalized individual VEI responses.

To investigate whether the relative strength of VEI responses to MEI depended on presenting single versus multiple VEIs, we implemented two bootstrapping strategies on the same dataset: averaging across 20 repeats of the same VEI, and averaging across 20 single trials from different VEIs. For each bootstrap iteration, we estimated a robust linear coefficient between VEIs and MEI using the RANSAC algorithm^67^. We then examined whether the difference in linear coefficients estimated from the two bootstrapping strategies differed from zero.

#### Decoding VEIs from population responses

To assess whether differences across VEIs can be represented by V1 population responses, we randomly selected a pair of VEIs (VEI_1_ and VEI_2_) for each neuron and presented each VEI 20 times. To quantify the neuronal discriminability between these VEIs, we implemented a fivefold cross-validated logistic classification with L2 regularization on the V1 population responses. This classifier was trained to distinguish whether each single-trial population response was originated from VEI_1_ or VEI_2_. The optimal regularization strength was determined empirically by fitting the logistic regression model on an independent pilot dataset.

### In silico quantification and analysis

#### In silico stimuli presentation

To ensure the most reliable predictions from our model, we standardized all images to match the training set statistics before presenting them in silico. The training set images on average had approximately a mean of zero and RMS contrast of 0.8 (after *z*-scoring) within the MEI mask. By synthesizing MEIs under a range of full-field statistics constraints, we determine that full-field images with a mean of zero and RMS contrast of 0.25 best replicated these statistics. Therefore, we adopted a uniform preprocessing procedure for all images: we applied the corresponding MEI mask to each image and then normalized the entire image to mean of zero and RMS contrast of 0.25.

#### Simulation of simple and complex cells

The response of an idealized simple cell was modeled as convolution with a 2D Gabor filter followed by a rectified linear activation function^17^. An idealized complex cell was formulated by the classical energy model^68^, where the response was modeled as the square root of the summation of squared outputs to a quadrature pair of 2D Gabor filters. A Gabor image was generated as

12 $$\begin{array}{l}{I}_{\mathrm{Gabor}}(x,y)=\exp -(\frac{{(x-{\mu }_{x})}^{2}+{(y-{\mu }_{y})}^{2}}{2{\sigma }^{2}}) \cos \left(\frac{2\pi (x\cos \theta +y\sin \theta )}{\lambda }+\psi \right)\end{array},$$

where *μ*_*x*_ and *μ*_*y*_ control the center of the Gabor, *σ* is the standard deviation of the Gaussian envelope, and *θ*, *λ* and *ψ* control the orientation, spatial frequency, and phase of the grating, respectively. For all simulated cells, *μ*_*x*_ and *μ*_*y*_ were set to zero; *θ* and *psi* were randomly sampled from the range of [0,*π*] and [0,2*π*], respectively. We then selected *σ* and *λ* values that closely match neuronal properties in our dataset. *σ* values were selected from the range of [4.4°, 10.9°] visual angle, as inferred from MEI mask sizes from 1,000 random neurons in eight pilot datasets. For *λ*, we first searched for Gabor images with the highest predicted activation for real neurons using a range of [0.02° per cycle, 0.12° per cycle]^36^, and then randomly selected *λ* values from those corresponding to the optimal Gabor images. We then randomly combined these parameters to simulate the ground-truth Gabor stimuli for 60 simple and 60 complex cells. To ensure sufficient frequency representation within the Gaussian window, we constrained *λ* to be no more than twice the value of *σ*.

For each simulated cell, we collected idealized responses to 5,000 random ImageNet images, using each response as the mean of a Poisson distribution from which we sampled a noisy response. This noisy input–response dataset was then used to train a predictive model with an architecture identical to that used for real neurons. Finally, we applied the same image optimization procedure described above to generate MEI and VEIs using the simple and complex cell predictive models. This procedure aims to simulate the noise inherent in biological systems and create predictive models for simulated cells that more closely resemble those of real neurons.

#### Representational similarity

To quantify similarity of visual stimuli in a space that is relevant to mouse V1 population functionality, we first obtained a low-dimensional latent representation for each stimulus and then assessed the similarity between stimulus pairs using this latent representation. We used a model ensemble trained on a held-out dataset to predict population responses to a random set of MEIs from 14 different animals (500 per animal) after these MEIs were centered and standardized. We then performed principal component analysis (PCA) and retained the top 53 principal components, which together explained 95% of the response variance across all neurons. For any given stimulus, we centered and standardized it, passed it through the designated model ensemble, and projected the resultant population response onto the 53-dimensional space to derive its latent neuronal representation. We then computed representational similarity of each pair of stimuli using cosine similarity in this latent space. To compute similarity between sets of stimuli (for example, sets of VEIs generated from various conditions), we calculated the average pairwise representational similarity.

#### The CUB and CUB-grating datasets

To study the relationship between invariance and natural stimuli, we sampled over 1 million crops from the Caltech-UCSD Birds-200-2011 (CUB) image dataset^25^. This dataset contains 11,788 natural images across 200 bird categories, each featuring a single bird in its natural habitat. We resized original images to 64 × 64 pixels and sampled them using a 36 × 36pixel window with a stride of 2. Each image included a manual semantic segmentation label identifying the bird region as a probability map, which was binarized using a threshold of 0.5. To test whether object boundaries defined by spatial frequency differences strongly activate V1 neurons, we created a modified dataset, ‘CUB-grating’, by replacing object and background content with grating patterns. We generated four equally sized image types (2 million images each):

1. Homogeneous grating pattern without using segmentation labels (‘single grating’).
2. Gratings with identical spatial frequency but varying orientations.
3. Gratings with identical orientation but varying spatial frequencies.
4. Gratings with both spatial frequency and orientation varied.

To determine the frequency range for high- and low-frequency patterns, we sampled 1,000 random neurons across eight pilot mice and fitted optimal Gabor filter stimuli for each neuron using their corresponding predictive model. We defined high frequency as 5.83° per cycle and 15.55° per cycle (5th to 50th percentile) and low frequency as 15.55° per cycle to 58.3° per cycle (50th to 95th percentile). We independently and uniformly sampled frequency, orientations and phases for the object and background gratings, and normalized them to have identical mean and RMS contrast. We masked the grating images with their corresponding object and background masks from the binarized segmentation label. To minimize edge artifacts, we applied a Gaussian filter (*σ* = 1.5) to blur object-background boundaries.

#### Analyses on highly activating crops in the CUB and CUB-grating datasets

To assess the alignment of the spatial structure between neurons’ bipartite RF and the object-background division in the CUB dataset, we screened over 1 million CUB image crops in silico for each target neuron to identify the 100 most highly activating ones. Each crop was masked from a full-field image with the target neuron’s MEI mask and standardized to match the MEI’s mean and RMS contrast within the mask. We classified a crop as containing object boundary if it comprised at least 20% of both object and background within the target neuron’s RF. We also reproduced our findings with 10% and 30% thresholds. Crops without object boundaries were excluded from downstream analyses. To obtain a bipartite mask (*m*_bipartite_) for each neuron, we binarized its MEI mask (${m}_{{\mathrm{MEI}}_{b}}$) and variable subfield mask (${m}_{{V}_{b}}$) by thresholding at 0.3, assigning a value of 1 for each pixel within ${m}_{{V}_{b}}$ and −1 for each remaining pixel within ${m}_{{\mathrm{MEI}}_{b}}$. Similarly, using the manual segmentation label for each image crop, we assigned a value of 1 if the pixel is within the object and −1 if the pixel belongs to the background. We quantified the alignment between a crop’s segmentation label and the neuron’s bipartite mask using a matching score defined as $s=\frac{\sum {m}_{\mathrm{bipartite}}\times {m}_{seg}}{\sum {m}_{{\mathrm{MEI}}_{b}}}$, where a score of 1 indicates perfect alignment of the variable subfield with the object and fixed subfield with the background, and 0 indicates the reverse. The same procedure was applied on 100 random images to serve as a baseline to account for inherent bias of the dataset. This protocol was also used to evaluate matching in the CUB-grating dataset.

#### Spatial frequency tuning analysis in bipartite subfields

To study systematically how neuronal responses vary with spatial frequency in each subfield, we performed additional analyses using natural images. For each target neuron, we applied the fixed subfield mask to the CUB natural image dataset to extract 10,000 random crops and computed their median spatial frequency. These crops were then combined with the original variable subfield (masked directly from the MEI), and the resulting images were fed into our predictive model to obtain predicted responses. For each neuron, we calculated the Pearson correlation coefficient between the median frequency of the crops and the predicted responses. We repeated this process for the variable subfield as well.

### Functional connectomics analyses on the MICrONS dataset

#### Replication of VEIs in MICrONS and closed-loop validation

Recently, a large-scale functional connectomics dataset of mouse visual cortex (‘MICrONS’), including responses of >75,000 neurons to full-field natural videos and the reconstructed subcellular connectivity of the same cells from electron microscopy data^8^. A dynamic digital twin model^9^ of mouse visual cortex exhibits high predictive performance for natural videos and generalizes accurately to out-of-domain stimuli, including drifting Gabor filters, directional pink noise, and random dot kinematograms. Leveraging this cross-domain generalization, we extracted specific functional properties from this digital twin model and related them to the neuronal connectivity and anatomical properties. Specifically, we first trained a dynamic digital twin based on a convolutional vision transformer with a long short-term memory architecture (CvT-LSTM)^9^ on population responses from the MICrONS dataset population using all video clips from the MICrONS stimulus set. We then presented a video of 5,100 unique natural images as described in ‘Presentation of natural stimuli’ (except that every image was shown once as the model prediction is deterministic) to the dynamic digital twin and used these in silico responses to train a static model (‘dynamic-static’ model). MEIs, VEIs and the bipartite parameterization were subsequently extracted from the ‘dynamic-static’ model.

To validate the images generated from this ‘dynamic-static’ model, we recorded the visual responses of the same neuronal population to static natural images as well as to the identical natural movies that were used in the MICrONS dataset in three new mice. Based on neuronal responses we trained two versions of static models: one directly on in vivo static image responses as described in previous sections, and one ‘dynamic-static’ model. We then presented MEIs, VEIs, and partial-texture VEIs extracted from both static models back to the mice in closed-loop experiments. As the static and dynamic stimuli were presented in two separate scans on day 1, only neurons that had unique one-to-one matching between the two scans (54 ± 3%) and had matching distance smaller than 5 μm (76 ± 5%) were considered for image synthesis. We further excluded neurons in the bottom 1st percentile of CC_max_ and CC_abs_ and then randomly selected neurons from the remaining population for closed-loop validation. For scans with synthesized images, only neurons with reliable matching (see criteria in ‘Cell matching across days’) to both day 1 static and dynamics scans were included for data analysis. On average, 33 ± 2% of closed-loop neurons per scan were kept for data analysis.

#### Neuron selection for functional connectomics analysis

We focused our analysis exclusively on V1 L2/3 excitatory neurons, using per-neuron area membership labels provided by the MICrONS release^8,26^. Neurons with reliable visual responses (CC_max_ > 0.4) and that were well predicted by the digital twin model (CC_abs_ > 0.2) were included in the downstream analysis, following the methodology described in Schoppe et al.^12^ and Ding et al.^26^. These criteria resulted in 19 presynaptic neurons and 706 connected pairs for downstream analysis.

To control for neuronal connectivity at a finer synaptic level, we followed the procedure outlined in Ding et al.^26^ to identify axonal–dendritic proximity (ADP) controls. These neurons had a dendritic skeleton passing within 5 μm of the presynaptic neuron’s axonal skeleton (measured in 3D Euclidean distance) but were not observed to form a synapse with the presynaptic neuron. This process produced 2,486 ADP neurons and 18,162 pairs of ADP controls.

#### Functional analysis on the MICrONS dataset

To elucidate functional differences between connected pairs and ADP controls, we aggregated data across all presynaptic neurons; however, naive aggregation is problematic due to varying functional and connectivity metrics of different presynaptic neurons.

To address this, we performed the following corrections:

Correction on functional metric: We implemented a two-step standardization process for each pairwise metric (MEI and VEI pairwise similarity, and diversity index difference). First, we adjusted the pairwise value by subtracting the mean of its presynaptic neuron, calculated across that neuron’s connected pairs and ADP control pairs. We then added back a regional baseline level, computed as the global mean value across all connected pairs and ADP controls within V1 L2/3. This correction was applied to all pairwise metrics for both connected pairs and ADP controls.

Correction on connectivity metric: When aggregating connected pairs, we weighted each pair by the number of synapses observed between them and then adjusted for presynaptic neuron synapse conversion rates. We calculated the synapse conversion rate for each presynaptic neuron as the ratio between the total number of synapses formed from its axon and the total co-traveling distance with dendrites from its postsynaptic targets and ADP controls within V1 L2/3. The expected number of synapses between a pair was then calculated as the product of this rate and the co-traveling distance. We adjusted the observed number of synapses by this expected value and then added back a regional expected number of synapses based on the pair’s co-traveling distance and the regional synapse conversion rate.

We then performed weighted bootstrapping on connected and ADP pairs independently, using the adjusted number of synapses as weight for connected pairs and co-traveling distance for ADP controls. To quantify synapse conversion rate as a function of functional similarity, we adapted the procedure from Ding et al.^26^. We binned all neuron pairs (both connected and ADP control) according to their pairwise value. For each bin, synapse conversion rate was defined as the ratio of the number of observed synapses to the total co-traveling distance between presynaptic neurons’ axon arbors and their targets’ dendritic skeletons within the bin. We included only bins containing more than ten connected neuron pairs and representing at least than 2.5% of all connected neuron pairs. To estimate the standard deviation (s.d.) of the synapse conversion rate, we resampled the connected and ADP pairs with replacement, binned the resampled distributions, and calculated the s.d. within each bin.

### Statistics

Given the exploratory nature of this study, no statistical methods were used to predetermine sample size. We acquired two-photon calcium imaging data from 17 mice (>33,000 neurons) for network training and downstream analyses, and Neuropixels recordings from six mice (>300 neurons) for complementary analyses. Sample sizes matched or exceeded those of previous studies with similar designs. All statistical tests were reported directly in figure captions with corresponding sample sizes, test statistics and *P* values. Permutation tests and bootstrapping procedures were conducted using 10,000 permutations or resamplings with replacement. *P* values for permutation tests and bootstrapping <10^−4^ were reported as *P* < 10^−4^; otherwise, exact *P* values were provided. The linear coefficient was computed as the average of values obtained from 1,000 independent robust regressions using the RANSAC algorithm^67^. For Welch’s *t*-tests and one-sample *t*-tests, normality was assumed but not explicitly tested. For multiple comparisons, we applied the Benjamini–Hochberg correction and reported both the fraction of significant comparisons before and after correction, along with the corrected *P* values.

### Software

Experiments and analysis were conducted with custom-built data pipelines (https://github.com/cajal/pipeline) and a custom stimulus optimization pipeline (https://github.com/cajal/featurevis). The data pipeline was developed in MATLAB (v.2016a, v.2018b) and Python (v.3.6, v.3.8) and Psychtoolbox3, ScanImage (v.2017b), DeepLabCut (v.2.0.5), CAIMAN (v.1.0) and LabView (v.2016) were used for data collection. DataJoint (v.0.12.9), MySQL (v.5.7.37) and CAVE (v.4.12,4.14,4.16) were used for storing and managing data. Numpy (v.1.22.2), pandas (v.1.5.3), SciPy (v.1.8.0), statsmodels (v.0.13.5), scikit-learn (v.1.0.2) and PyTorch (v.1.7.0+cu110) were used for model training and statistical analysis. Matplotlib (v.3.2.2) and seaborn (v.0.11.2) were used for graphical visualization. Jupyter (v.4.4.0), Docker (v.19.09.13) and Kubernetes (v.1.19.4) were used for code development and deployment.

### Reporting summary

Further information on research design is available in the Nature Portfolio Reporting Summary linked to this article.

## Online content

Any methods, additional references, Nature Portfolio reporting summaries, source data, extended data, supplementary information, acknowledgements, peer review information; details of author contributions and competing interests; and statements of data and code availability are available at 10.1038/s41593-026-02213-3.

## Supplementary information

Supplementary Information Supplementary Figs. 1–15.

Reporting Summary

## Source data

Source Data Statistical source data for Figs. 1–6 and Extended Data Figs. 1–10.

## Data Availability

The public image dataset CUB-200-2011 used in this study is available at https://www.vision.caltech.edu/datasets/cub_200_2011. All other data supporting the findings of this work have been deposited on GIN (G-Node) and are publicly available at https://gin.g-node.org/cajal/microns-vei-2025 (10.12751/g-node.w7feg2). Source data are provided with this paper.

## Code availability

Custom developed code used in the analysis can be found at https://github.com/cajal/microns-vei-2025, tag 1.0.0.
