# Supplementary material for: Functional bipartite invariance in mouse primary visual cortex receptive fields
Source: Nat Neurosci. 2026 Feb 25;29(4):851–63. doi: 10.1038/s41593-026-02213-3 (PMC13061618; doi:10.1038/s41593-026-02213-3)
Supplement: Supplementary file 1 — Supplementary Figs. 1–15. [file 41593_2026_2213_MOESM1_ESM.pdf]

nature neuroscience

Supplementary information

https://doi.org/10.1038/s41593-026-02213-3

Functional bipartite invariance in mouse primary visual cortex receptive fields

In the format provided by the authors and unedited

Supplementary Information

Supplementary Fig. 1 - Upper bound of the correlation coefficient and test correlation coefficient of V1 neurons.
Supplementary Fig. 2 - Example stimuli presented in closed-loop experiments.
Supplementary Fig. 3 - MEI and VEIs activated neurons with high specificity in all mice.
Supplementary Fig. 4 - Similarity between MEI and VEI in vivo responses was not inflated by trial-to-trial eye movement.
Supplementary Fig. 5 - Excluding neurons with small variable subfields did not alter the similarity between VEI and partial-texture VEI in vivo responses.
Supplementary Fig. 6 - VEI closed-loop verification for randomly selected neurons.
Supplementary Fig. 7 - Example MEI, VEIs, and partial-texture VEIs from electrophysiological recordings.
Supplementary Fig. 8 - Bipartite receptive field cannot be explained by either trial-to-trial eye movement or spatial readout location variation.
Supplementary Fig. 9 - Bipartite masks aligned with object boundaries in highly activating natural crops.
Supplementary Fig. 10 - Alignment between bipartite mask and natural object boundaries was robust across different thresholds for classifying patches as containing object boundary.
Supplementary Fig. 11 - Dynamic static model in vivo validation.
Supplementary Fig. 12 - MEI activated neurons with high specificity in both static and dynamic-static models.
Supplementary Fig. 13 - VEIs activated neurons with high specificity in both static and dynamic-static models.
Supplementary Fig. 14 - Partial-texture VEIs activated neurons with high specificity in both static and dynamic-static models.
Supplementary Fig. 15 - Postsynaptic neurons and ADP controls exhibited similar diversity indices.

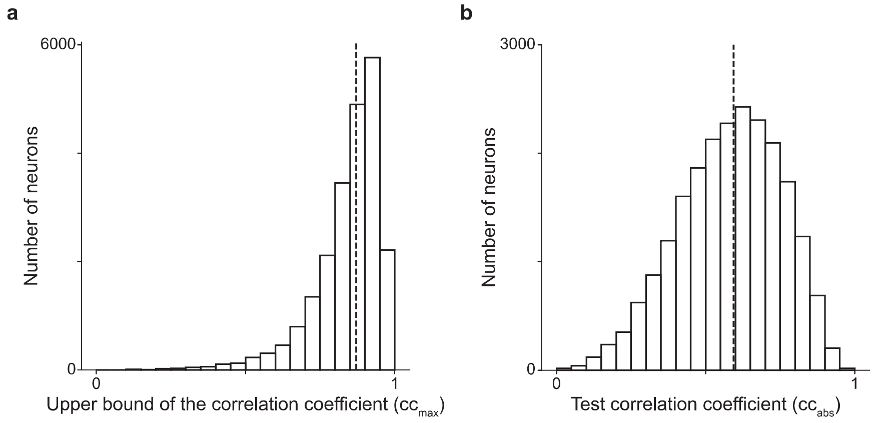

Supplemental Fig. S1. Upper bound of the correlation coefficient and test correlation coefficient of V1 neurons. **a**, Histogram of the upper bound of the correlation coefficient (CC_{max}) for V1 neurons. The dashed line indicates median value at 0.87. **b**, Histogram of the test correlation coefficient (CC_{abs}) for V1 neurons. The dashed line indicates median value at 0.60. Excessively noisy neurons ($CC_{max} < 0.1$) were excluded (0.19% of all neurons) and values outside of 0 and 1 are clipped (0.01% and 0.02% for **a** and **b**, respectively) for visualization. Data were pooled over 33,714 neurons from 14 mice.

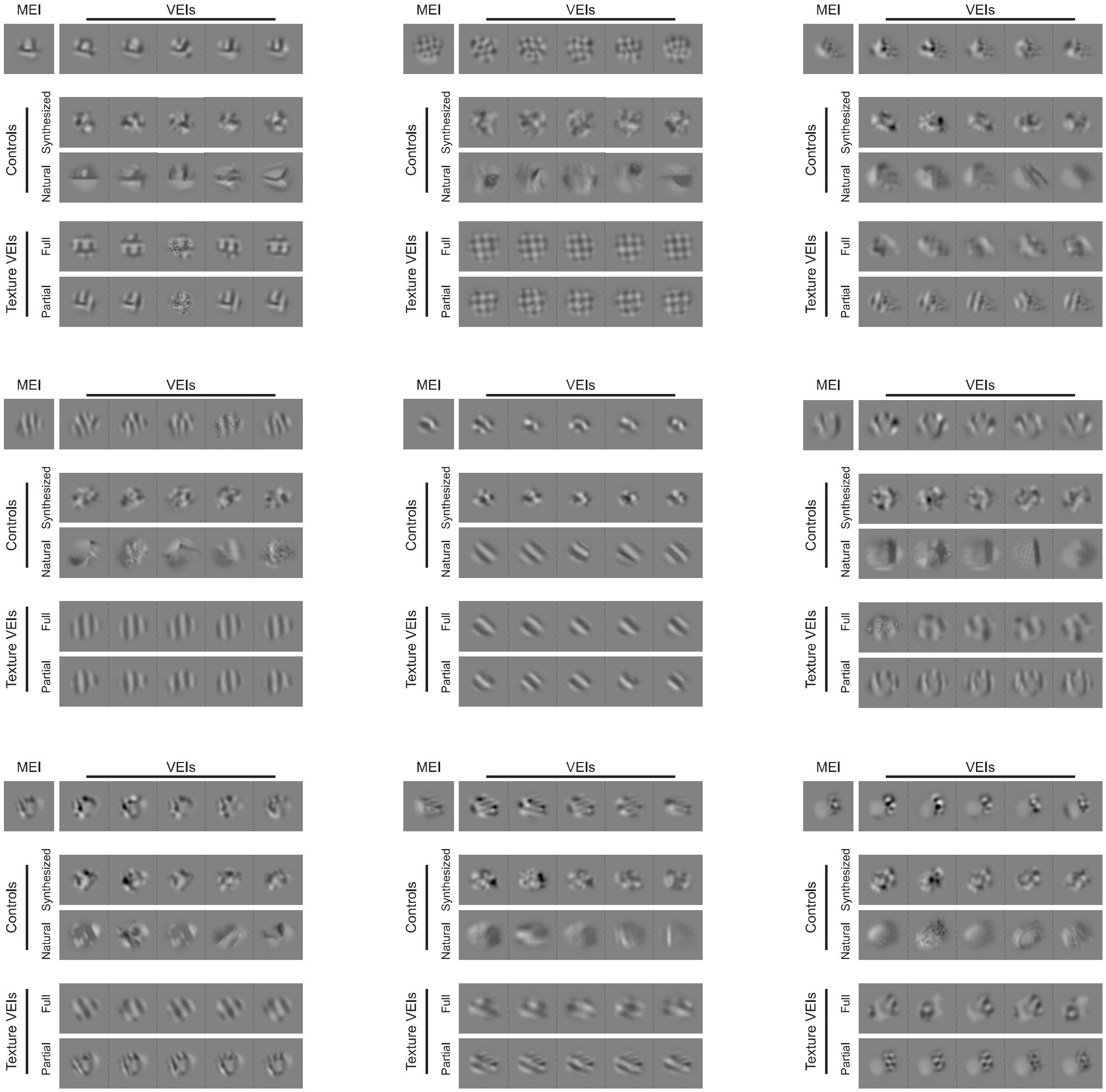

Supplemental Fig. S2. Example stimuli presented in closed-loop experiments. MEI, VEIs, VEI controls, and texture-based VEIs that were presented back to the animals in closed-loop experiments for nine example neurons.

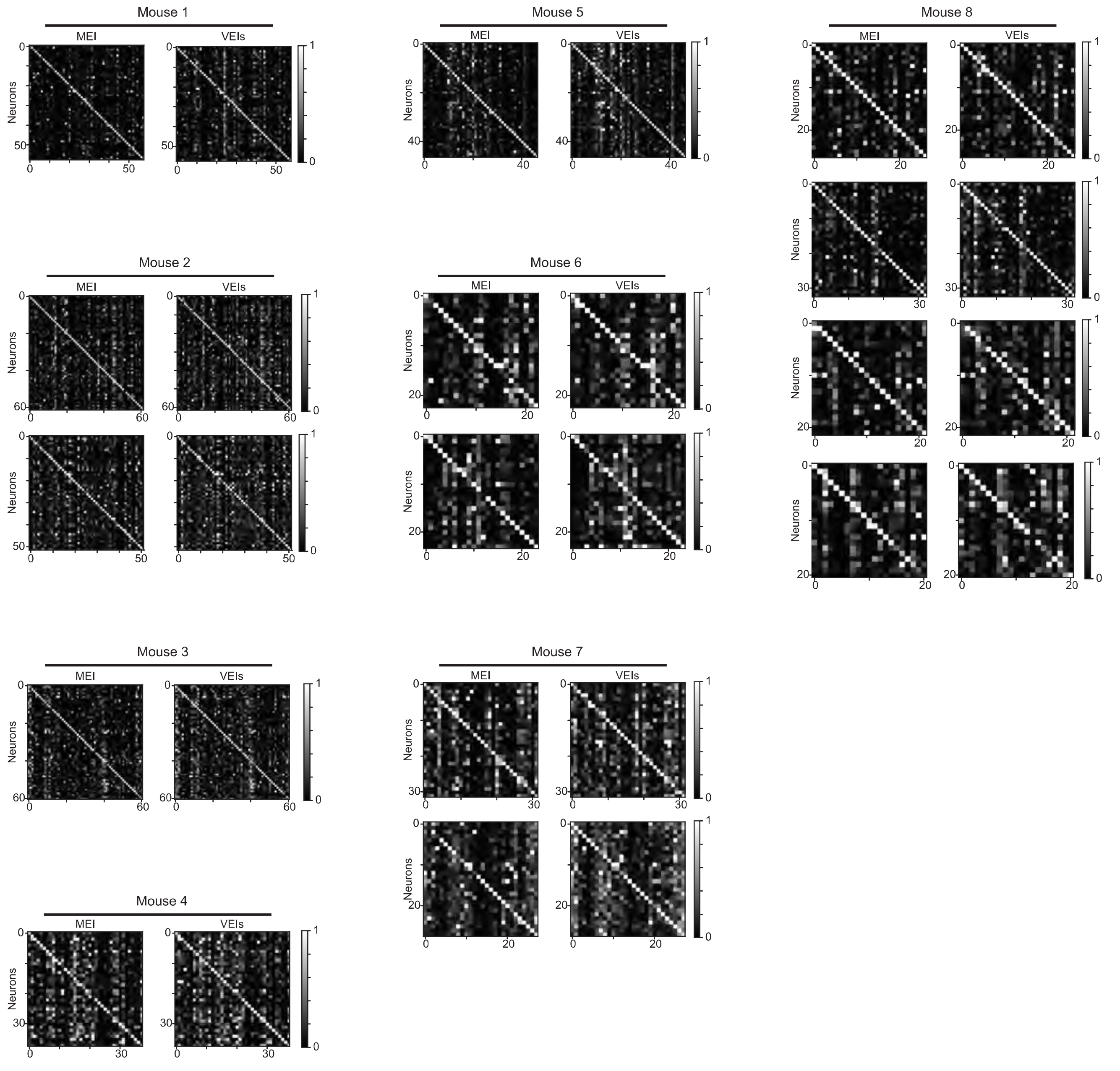

Supplemental Fig. S3. MEI and VEIs activated neurons with high specificity in all mice. The confusion matrices showed the responses of each neuron to the MEI (left) and VEIs (right) of all target neurons in individual scans where we presented the stimuli back to the mouse in closed-loop experiments. MEI responses were averaged across 20 repeats of the same image while VEIs responses were averaged across 20 different images with single repeat. The responses of each neuron were normalized, and each row was scaled so the maximum response across all images equaled 1. Responses of neurons to their own MEI and VEIs (along the diagonal) were larger than to other MEIs and VEIs, respectively (two-sided permutation test, $P < 10^{-4}$ for both cases across all mice after BH correction).

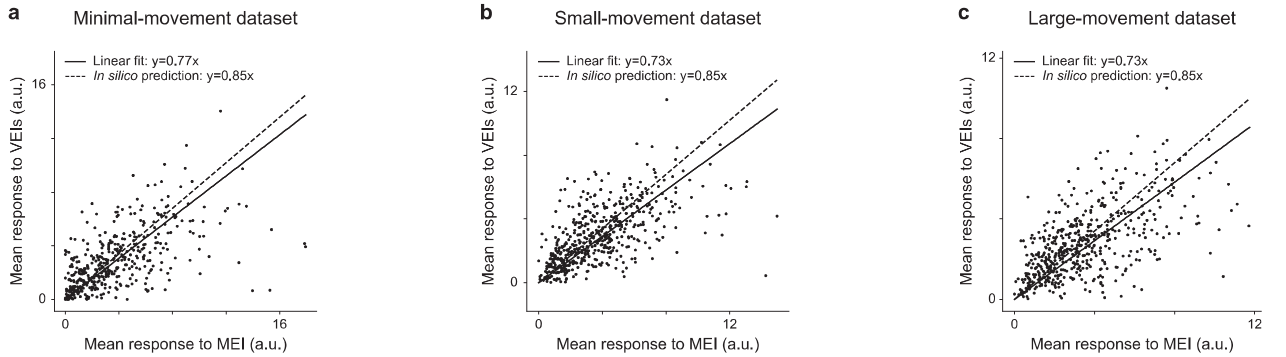

Supplemental Fig. S4. Similarity between MEI and VEI in vivo responses was not inflated by trial-to-trial eye movement. a–c, We selected MEI and VEI trials based on different thresholds of eye movement size: minimal-movement trials **a**, including only trials with minor deviation from the average pupil position (approximately $19.3 \pm 12.3\%$ of all trials), small-movement trials **b**, including trials within the bottom 50th percentile of pupil movement, and large-movement trials **c**, including trials within the top 50th percentile (Methods). The similarity between DEIs and MEIs in vivo responses were highly robust across all these three conditions ($77 \pm 5\%$, $73 \pm 4\%$, $73 \pm 3\%$ for minimal-movement, small-movement, and large-movement trials, respectively). Data were pooled over 500 neurons from eight mice.

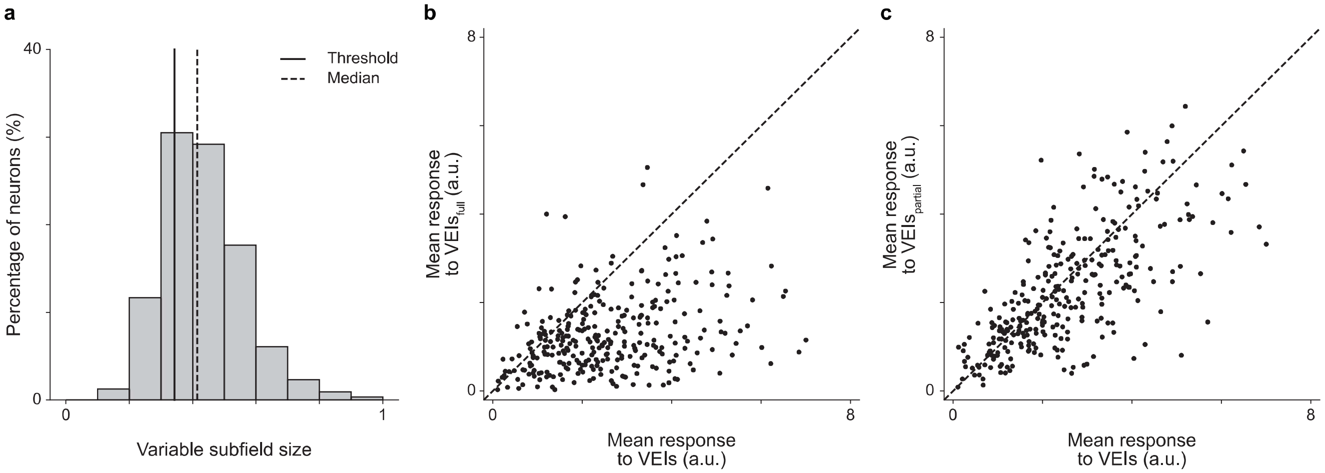

Supplemental Fig. S5. Excluding neurons with small variable subfields did not alter the similarity between VEI and partial-texture VEI in vivo responses. a, Histogram of variable subfield size for closed-loop neurons from Fig. 4e,f (median 0.40). **b, c**, Neurons with variable subfield sizes smaller than or equal to the 25th percentile (0.34) in **a** were excluded. **b**, VEIs$_{full}$ elicited weaker responses in their target neurons compared to VEIs (two-sided Wilcoxon signed-rank test, $W = 2853$, $P < 10^{-9}$) with 40.3% of all neurons showing different responses (25.7% after BH correction; $P < 0.05$, two-sided Welch's t-test with 30.1 average d.f.). **c**, VEIs$_{partial}$ activated their target neurons similarly to VEIs (two-sided Wilcoxon signed-rank test, $W = 15920$, $P = 9.6 \times 10^{-6}$) with only 7.7% of all neurons showing different responses (0.0% after BH correction; $P < 0.05$, two-sided Welch's t-test with 33.6 average d.f.). Data were pooled over 401 neurons from eight mice.

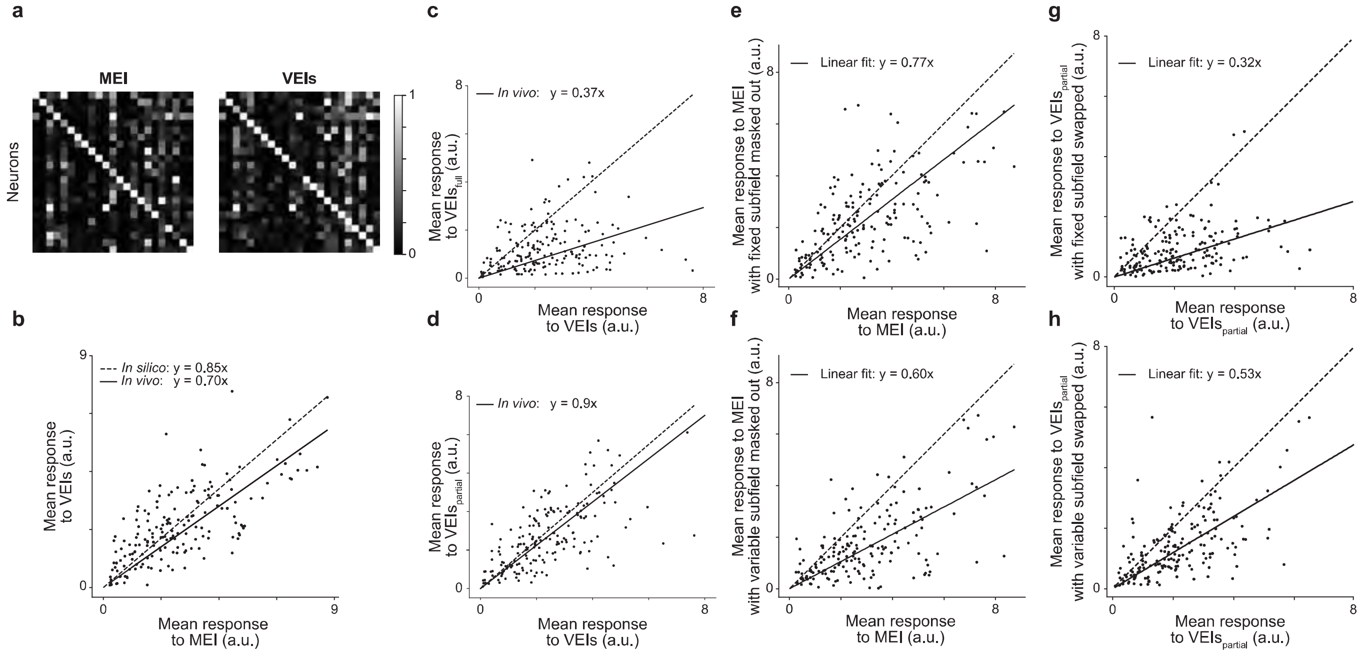

Supplemental Fig. S6. VEI closed-loop verification for randomly selected neurons. a, Confusion matrices for MEI and VEIs showed the responses of each neuron to the MEI (left) and VEIs (right) across all target neurons. The responses of each neuron were normalized, and each row was scaled so the maximum response across all images equaled 1. Responses of neurons to their own MEI and VEIs (along the diagonal) were larger than to other MEIs and VEIs (two-sided permutation test, $P < 10^{-4}$ for both cases). **b–h**, Each point represented the average response of a single neuron over 20 repeats of its MEI or 20 different stimuli of the same type with single repeat. **b**, VEIs stimulated neurons in vivo closely to the level predicted in silico with respect to MEI ($70 \pm 6\%$ versus 85%) (two-sided Wilcoxon signed-rank test, $W = 7257$, $P = 0.25$), with only 9.5% neurons showing different responses between VEIs and 85% of MEI (0.0% after BH correction; $P < 0.05$, two-sided Welch's t-test with 32.5 average d.f.). **c**, Full-texture VEIs ($\text{VEIs}_{\text{full}}$) evoked weaker responses in their target neurons than VEIs (two-sided Wilcoxon signed-rank test, $W = 1988$, $P < 10^{-9}$) with 35.2% of all neurons showing different responses than VEIs (15.1% after BH correction; $P < 0.05$, two-sided Welch's t-test with 29.4 average d.f.) **d**, Partial-texture VEIs ($\text{VEIs}_{\text{partial}}$) activated their target neurons similarly to VEIs (two-sided Wilcoxon signed-rank test, $W = 6693$, $P = 0.05$) with only 8.9% of neurons showing different responses from corresponding VEIs (0.0% after BH correction; $P < 0.05$, two-sided Welch's t-test with 33.1 average d.f.). **e, f**, MEIs with either fixed or variable subfields masked out evoked weaker responses in their target neurons than MEIs (two-sided Wilcoxon signed-rank test, $W = 4484$, $P < 10^{-9}$, and $W = 1401$, $P = 2.7 \times 10^{-7}$, respectively) with 20.1% and 31.3% of neurons showing weaker responses than MEIs (3.9% and 16.2% after BH correction; $P < 0.05$, two-sided Welch's t-test with 32.6 and 31.1 average d.f., respectively). **g, h**, $\text{VEIs}_{\text{partial}}$ with either fixed or variable subfield swapped evoked weaker responses in their target neurons than $\text{VEIs}_{\text{partial}}$ (two-sided Wilcoxon signed-rank test, $W = 1005$, $P < 10^{-9}$, and $W = 2026$, $P < 10^{-9}$, respectively) with 43.0% and 17.3% neurons showing weaker responses than $\text{VEIs}_{\text{partial}}$ (22.9% and 0.6% after BH correction; $P < 0.05$, two-sided Welch's t-test with 28.0 and 31.2 average d.f., respectively). Data were pooled over 179 neurons from three mice.

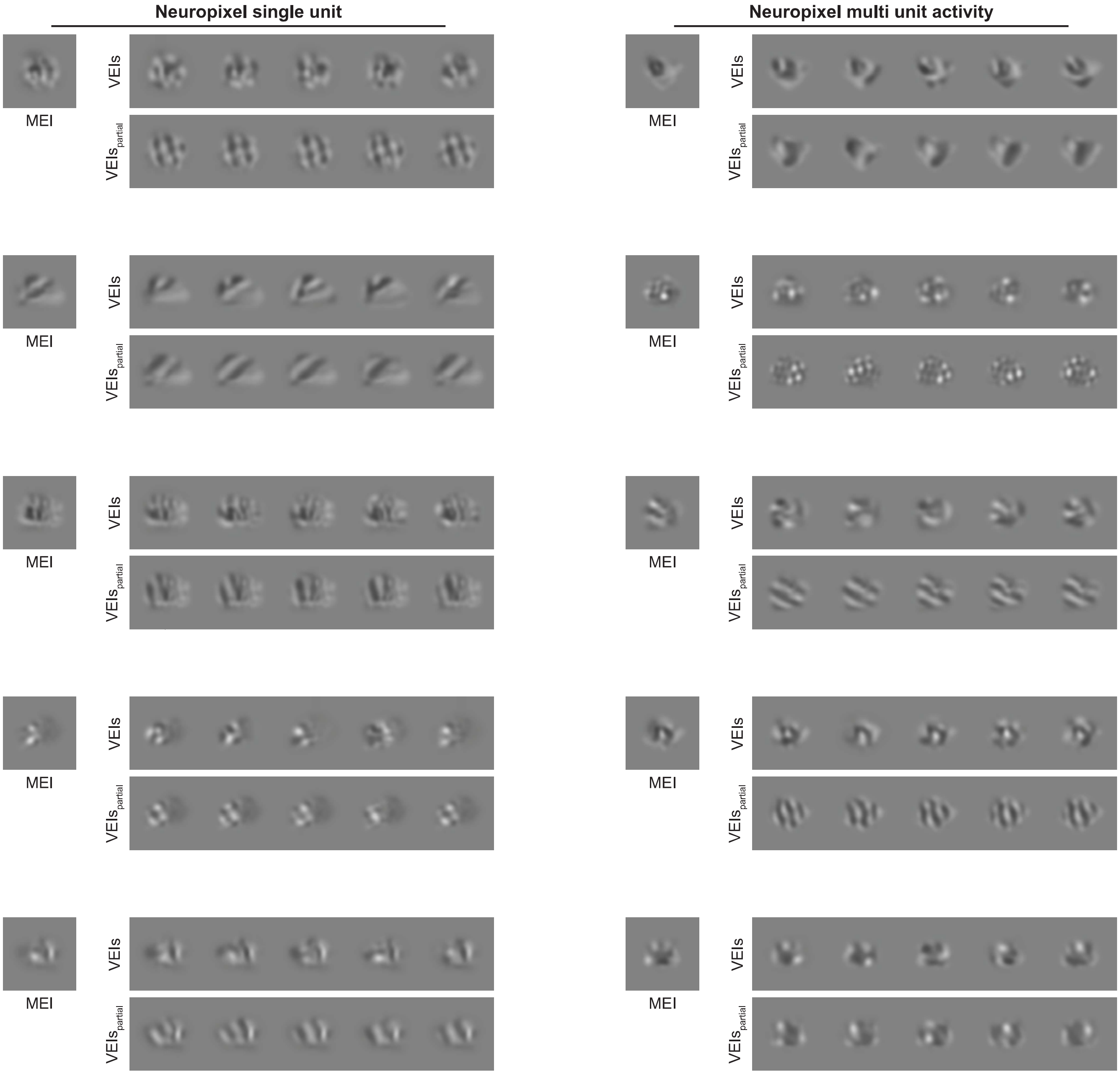

Supplemental Fig. S7. Example MEI, VEIs, and partial-texture VEIs from electrophysiological recordings. Left, Neuropixels single unit; right, Neuropixels multi-unit activity.

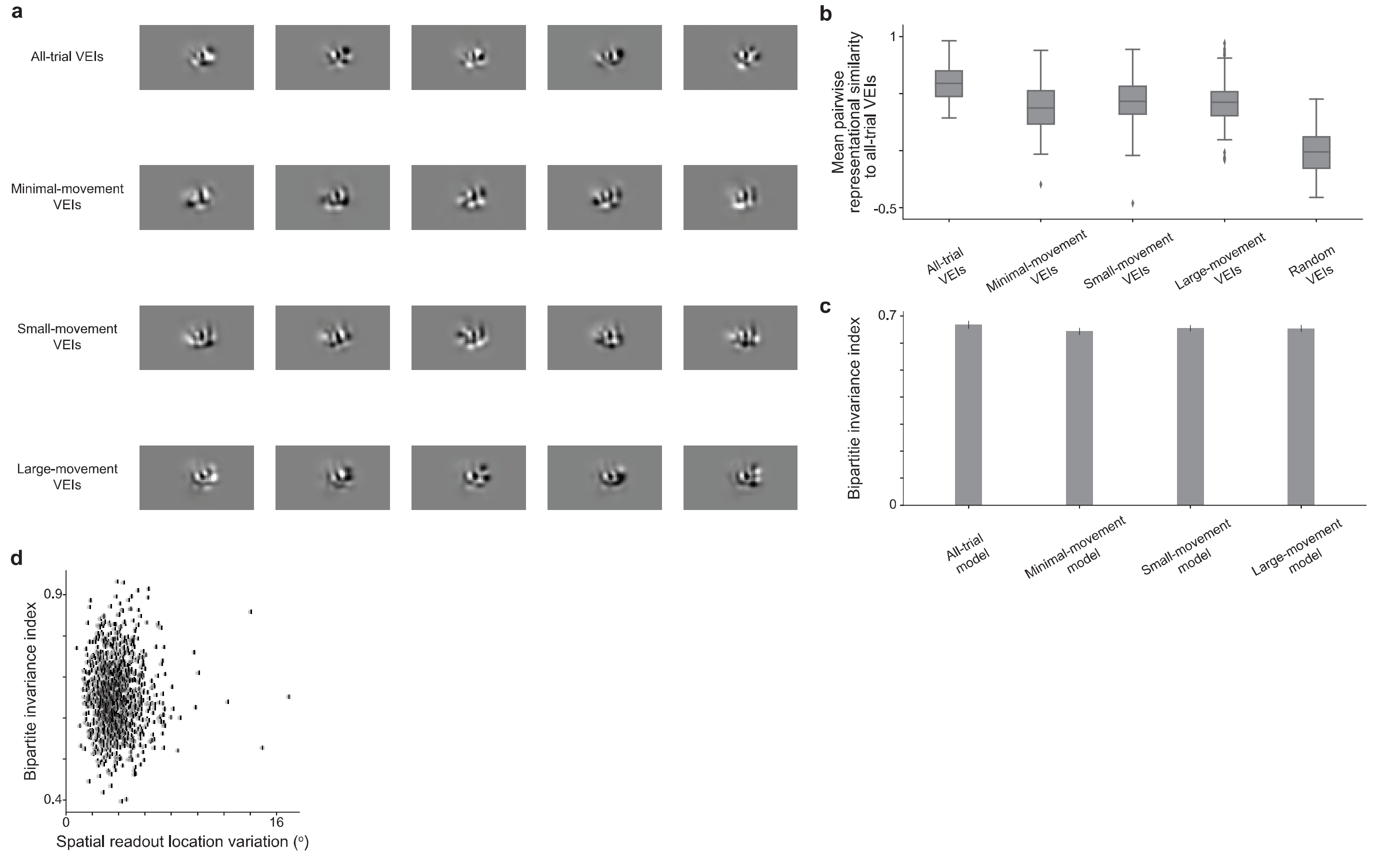

Supplemental Fig. S8. Bipartite receptive field cannot be explained by either trial-to-trial eye movement or spatial readout location variation. **a**, We created three sub-datasets from full-field natural image trials using inclusion criteria based on different thresholds of eye movement size: 1) minimal-movement trials only, 2) small-movement trials only, and 3) large-movement trials only (Methods). For each sub-dataset, an additional model was trained for the same neurons, in addition to the original model trained on all trials. VEIs from the original model as well as the three additional models for one example neuron were shown for visualization. **b**, VEIs synthesized from all three additional models were more similar to original VEIs than to random neuron VEIs as measured by the representational similarity (two-sided Wilcoxon signed-rank test, $W = 213, 121,$ and $101,$ respectively, with $P < 10^{-9}$ for all conditions after BH correction). **c**, Bipartite invariance indices were similar across all four models (one-way ANOVA $F = 0.97, P = 0.38$). **d**, Bipartite invariance indices were not correlated with the spatial readout location variation (Pearson $r = 0.037$, $P = 0.99$). Spatial readout location variation was defined as the mean Euclidean pairwise distance between individual model readout locations in visual degree. Data were pooled over 1200 randomly selected neurons from six mice.

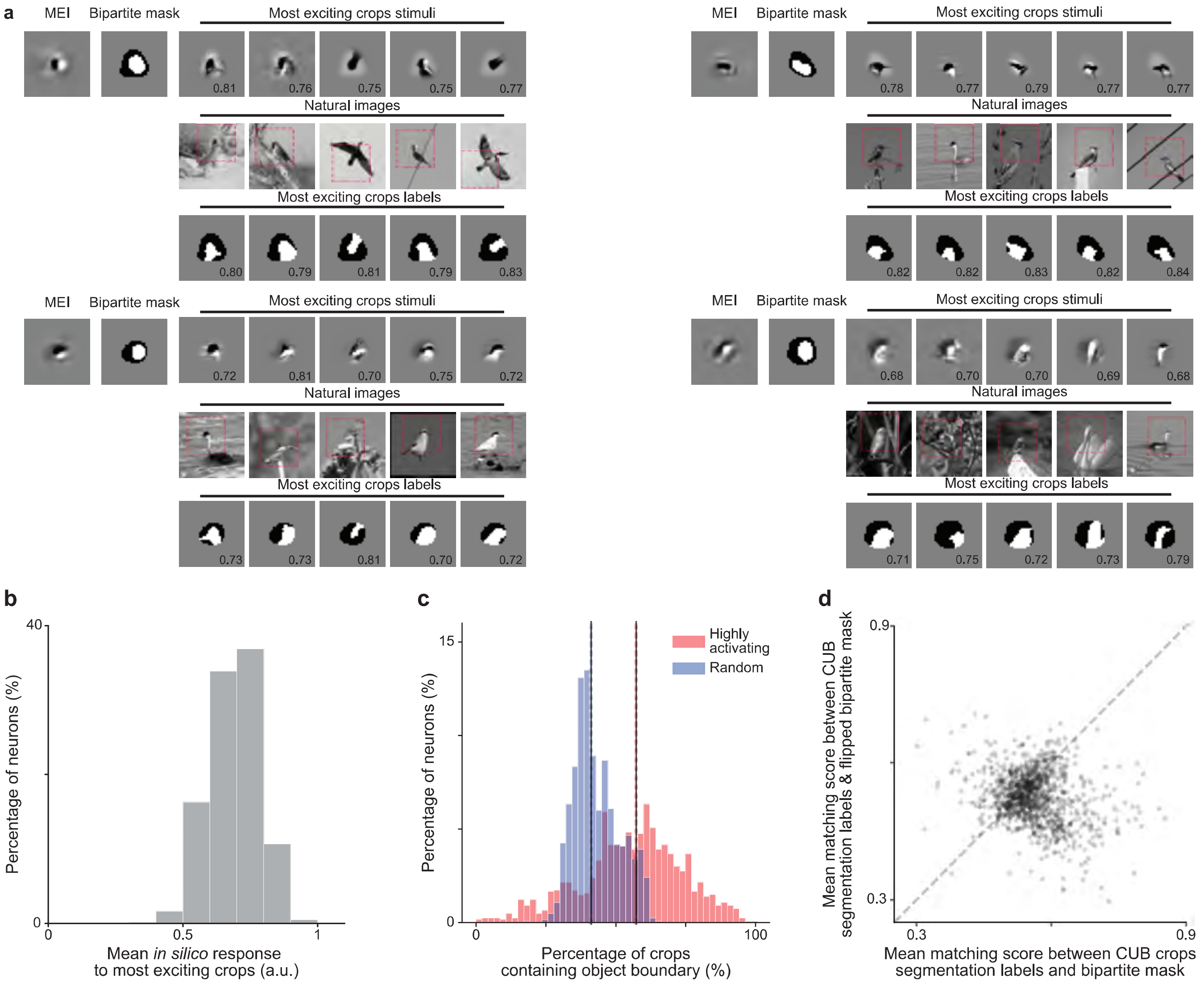

Supplemental Fig. S9. Bipartite masks aligned with object boundaries in highly activating natural crops. **a**, For each of the four example neurons, we showed the MEI and the bipartite mask (black, fixed subfield; white, variable subfield), along with five randomly selected crops from the top 100 most activating natural crops, the corresponding full-field natural images, and the segmentation labels. Red dashed boxes on the full-field natural images indicated the regions from which the crops were taken. For each crop, the in silico response normalized to the MEI response is shown in the bottom-right corner, and the corresponding matching score is shown in the bottom-right corner of the segmentation label. **b**, Histogram of mean in silico response for the top 100 most activating natural crops, normalized by the corresponding MEI in silico response (median 0.70). **c**, Highly activating natural crops were more likely to contain object boundaries compared to random crops (two-sided Wilcoxon signed-rank test, $W = 573606$, $P < 10^{-9}$). **d**, Flipping the bipartite mask with respect to the center of the RF decreased the matching score for the highly activating natural crops (two-sided Wilcoxon signed-rank test $W = 495850$, $P < 10^{-9}$) with 41.9% of all neurons showing weaker matching scores than original bipartite mask (37.6% after BH correction; $P < 0.05$, two-sided Welch's t-test with 99.0 average d.f.). One neuron was excluded from the analysis as it strictly preferred patches without object boundary. Data were pooled over 1200 randomly selected neurons from six mice.

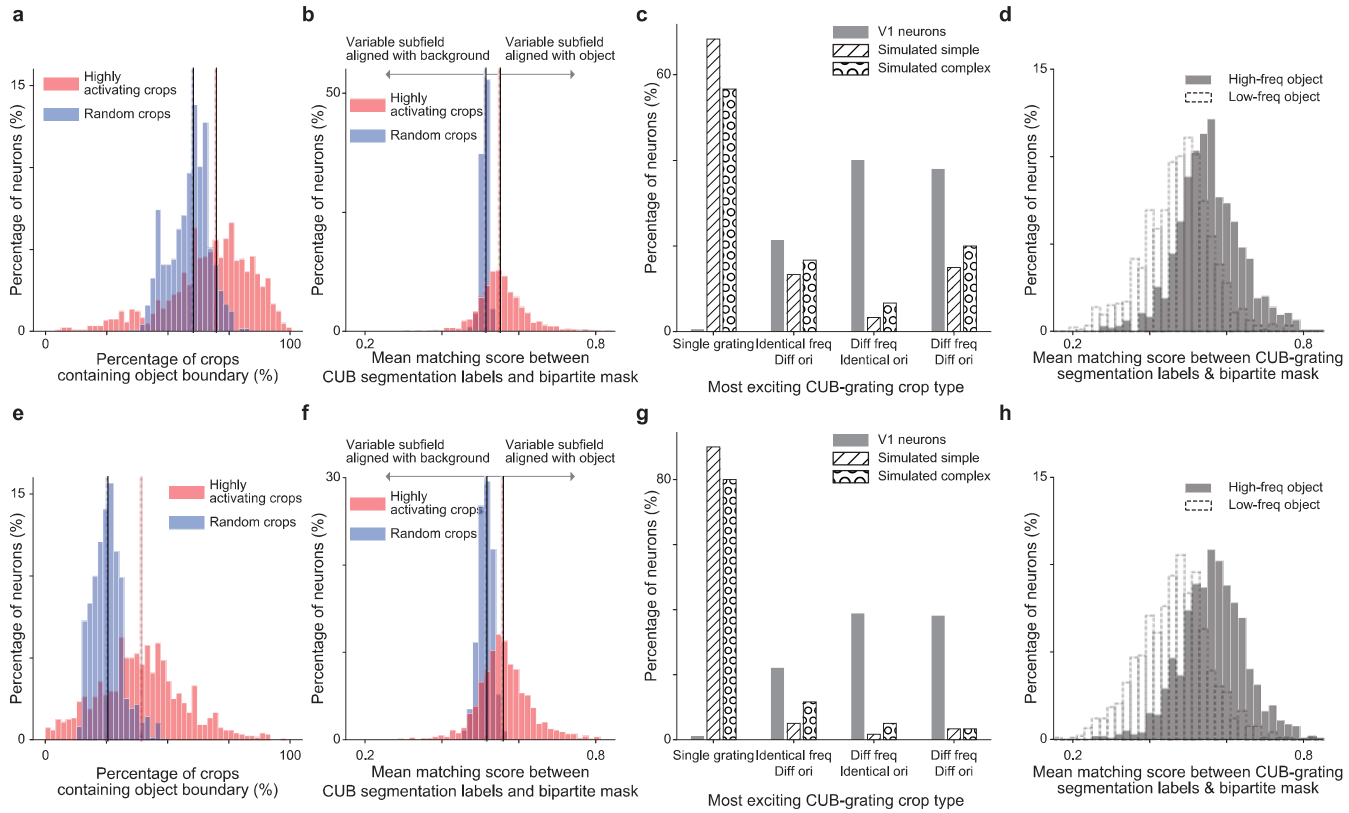

Supplemental Fig. S10. Alignment between bipartite mask and natural object boundaries was robust across different thresholds for classifying patches as containing object boundary. We systematically varied the minimum fraction of both object and background required within the RF to classify a patch as containing object boundary. **a–d, e–h**, Patches were classified as containing an object boundary if they contained more than 10% (Condition 1) or 30% (Condition 2) of both object and background. **a, e**, Highly activating natural crops were more likely to contain object boundaries compared to randomly crops (two-sided Wilcoxon signed-rank test, $W = 545046$, $P < 10^{-9}$ and $W = 603265$, $P < 10^{-9}$ respectively). **b, f**, Highly activating CUB crops with object boundary yielded higher matching scores than random natural crops with object boundary (two-sided Wilcoxon signed-rank test, $W = 85378$, $P < 10^{-9}$ and $W = 92707$, $P < 10^{-9}$). Across neurons, 54.7% and 44.7% exhibited greater matching scores to highly activating crops than to random crops (50.8% and 38.2% after BH correction), while only 5.3% and 4.4% showed lower matching scores to highly activating crops (4.6% and 3.3% after BH correction; $P < 0.05$, two-sided Welch's t-test with 107.2 and 42.3 average d.f. respectively). Six cells were excluded from condition 2 for this analysis since they preferred patches without object boundaries (fewer than two of the top 100 most activating crops containing object boundaries). **c, g**, Simulated simple and complex cells predominantly prefer "single grating" image type (68.3%, 56.7% for condition 1 and 90.0%, 80.0% for condition 2, respectively). In contrast, only 0.5% and 1.2% of V1 neurons prefer 'single grating' across the two conditions (one-way chi-squared tests comparing V1 neurons to simulated simple and complex cells: condition 1, $\chi^2 = 6152$ and $\chi^2 = 2889$, both $P < 10^{-9}$; $\chi^2 = 15994$ and $\chi^2 = 8123$, both $P < 10^{-9}$). Instead, most neurons preferred 'different frequency but identical orientation' (40.1% and 38.8%), followed by 'different frequency and orientation' (38% and 38.1%), and 'identical frequency but different orientation' (21.4% and 22.0%) in the two conditions, respectively. **d, h**, Among neurons preferring different frequencies within the RF, matching score for highly activating crops were higher in 'high-frequency object' dataset than 'low-frequency object' dataset (two-sided Wilcoxon signed-rank test, $W = 336493$, $P < 10^{-9}$ and $W = 338329$, $P < 10^{-9}$ respectively) with 64.6% and 67.2% of all neurons showing greater matching scores (64.6% and 67.0% after BH correction) while only 24.7% and 19.5% of all neurons showing smaller matching scores (24.2% and 19.0% after BH correction) ($P < 0.05$, two-sided Welch's t-test with 182.8 and 145.5 average d.f., respectively). Simulated simple and complex cells composed of 60 neurons for each population. Data were pooled over 1200 randomly selected neurons from six mice.

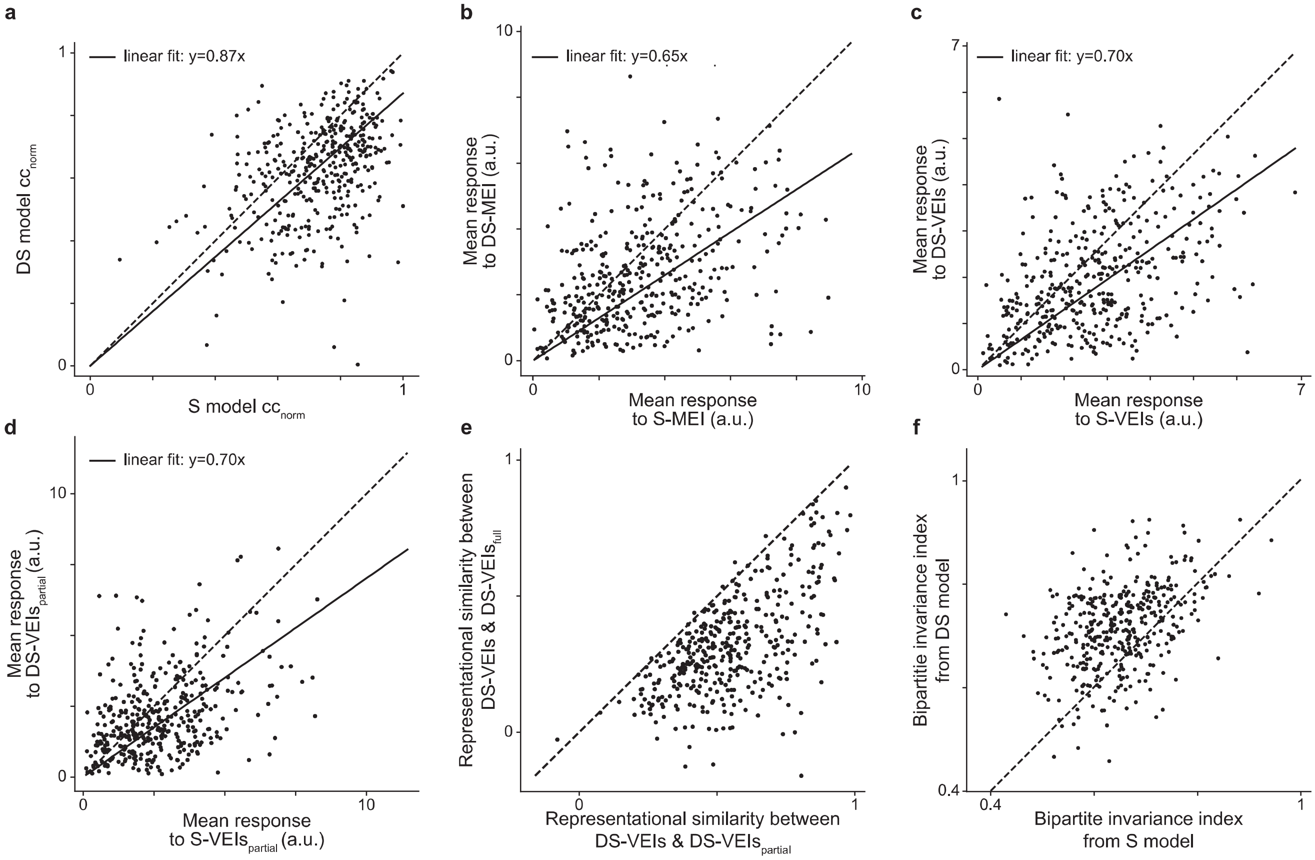

Supplemental Fig. S11. Dynamic static model in vivo validation a, Both static ('S') and dynamic-static ('DS') model yield high normalized correlation coefficient (CC_{norm}) for well-matched neurons (median $0.75, 0.65$, respectively). **b**, Both static MEI ('S-MEI') and dynamic-static MEI (('DS-MEI')) evoked high in vivo responses but static MEI evoked larger responses in target neurons than dynamic-static MEI (two-sided Wilcoxon signed-rank test, $W = 24493$, $P < 10^{-9}$) with 22.3% of all neurons showing higher responses to static MEI while 6.3% showing lower responses (10.8% and 2.3% after BH correction; $P < 0.05$, two-sided Welch's t-test with 32.0 average d.f.). **c**, Similarly, static VEIs evoked larger in vivo responses in target neurons more than dynamic-static VEIs (two-sided Wilcoxon signed-rank test, $W = 21414$, $P < 10^{-9}$) with 14.8% of all neurons showing higher responses to static VEIs while 2.5% showing lower responses (2.5% and 0.3% after BH correction; $P < 0.05$, two-sided Welch's t-test with 32.1 average d.f.). **d**, Static $\text{VEIs}_{\text{partial}}$ drove target neurons more than dynamic-static $\text{VEIs}_{\text{partial}}$ (two-sided Wilcoxon signed-rank test, $W = 25738?$, $P < 10^{-9}$) with 16.3% of all neurons showing higher responses to static $\text{VEIs}_{\text{partial}}$ while 5.5% showing lower responses (1.8% and 0.3% after BH correction; $P < 0.05$, two-sided Welch's t-test with 31.5 average d.f.). **e**, Dynamic-static $\text{VEIs}_{\text{partial}}$ were more similar to dynamic-static non-parametric VEIs than dynamic-static $\text{VEIs}_{\text{full}}$ (two-sided Wilcoxon signed-rank test, $W = 180$, $P < 10^{-9}$). **f**, Bipartite invariance indices from the dynamic-static were highly correlated with those from the static model (Pearson $r = 0.66, P < 0.05$, two-sided t-test). Data was collected from three mice, displaying a total of 399 neurons.

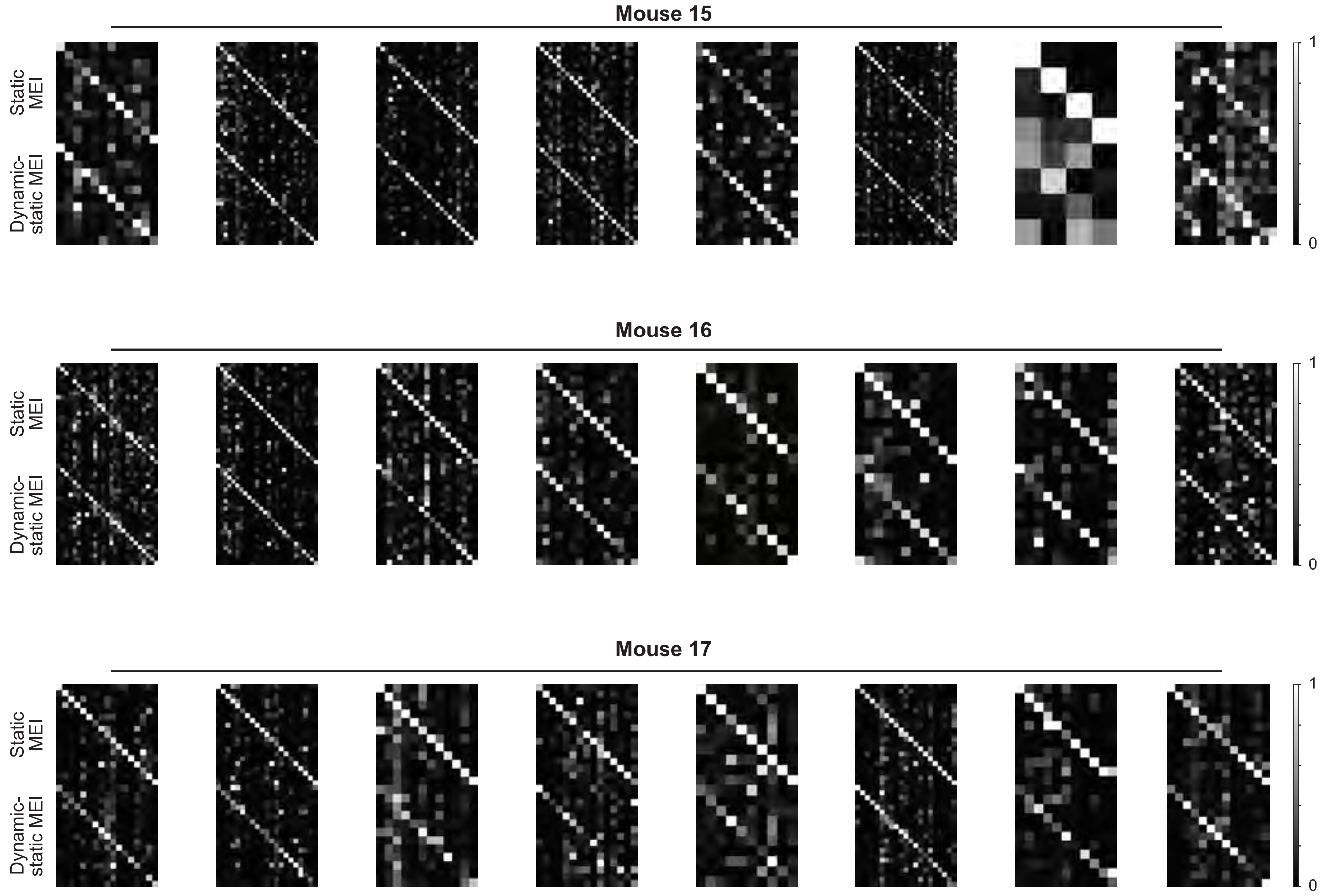

Supplemental Fig. S12. MEI activated neurons with high specificity in both static and dynamic-static models. The confusion matrices showed the responses of each neuron to static MEI (top) and dynamic-static MEI (bottom) of all target neurons in individual scans where we presented both models' stimuli back to the mouse in closed-loop experiments. MEI responses were averaged across 20 repeats of the same image. The responses of each neuron were normalized, and each row was scaled so the maximum response across all images equaled 1. Responses of neurons to their own MEI (along the diagonal) were larger than to other MEIs (two-sided permutation test, $P < 10^{-4}$ across all mice after BH correction).

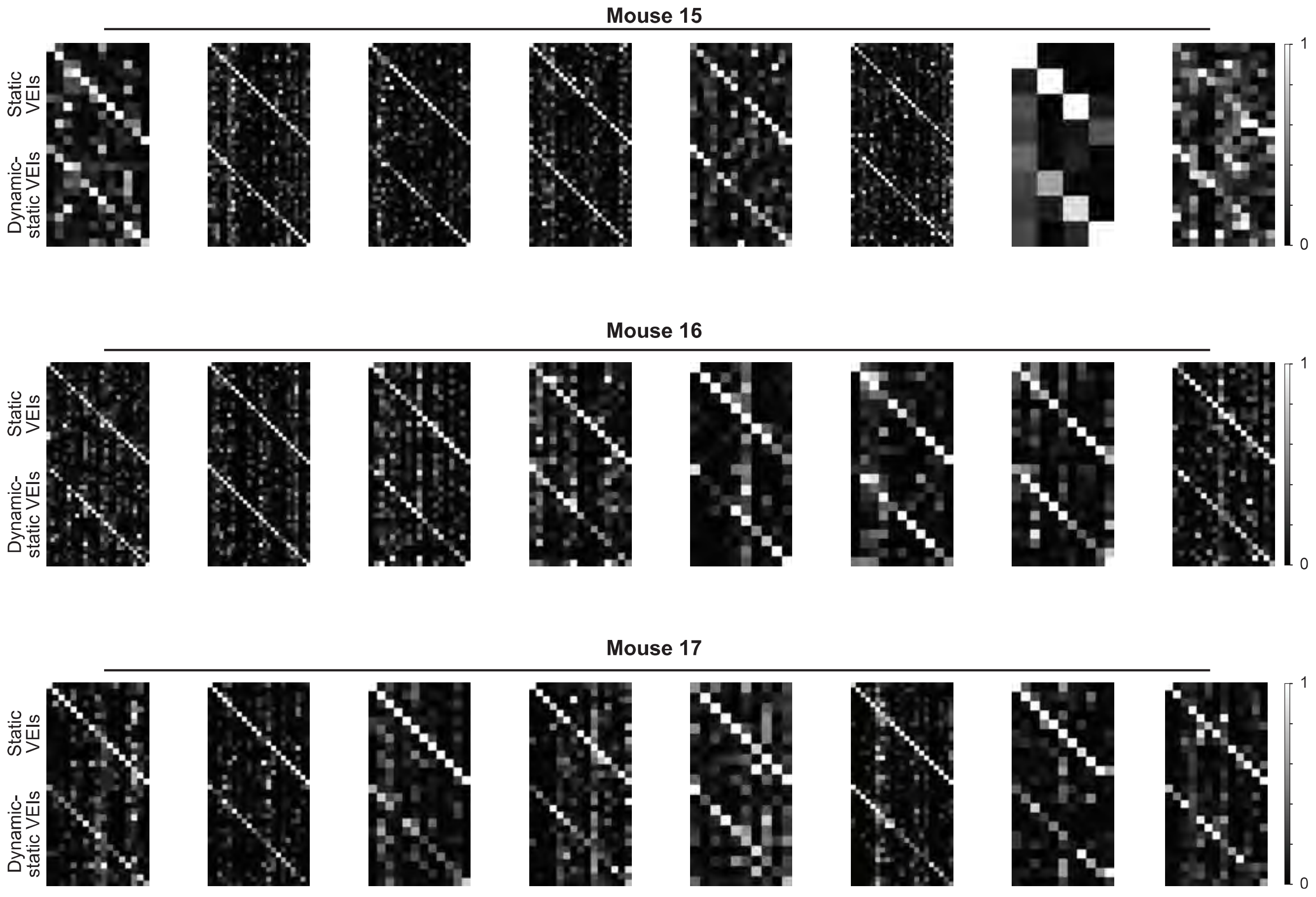

Supplemental Fig. S13. VEIs activated neurons with high specificity in both static and dynamic-static models. The confusion matrices showed the responses of each neuron to static VEIs (top) and dynamic-static VEIs (bottom) of all target neurons in individual scans where we presented both models' stimuli back to the mouse in closed-loop experiments. VEI responses were averaged across 20 different images with single repeat. The responses of each neuron were normalized, and each row was scaled so the maximum response across all images equaled 1. Responses of neurons to their own VEIs (along the diagonal) were larger than to other VEIs (two-sided permutation test, $P < 10^{-4}$ across all mice after BH correction).

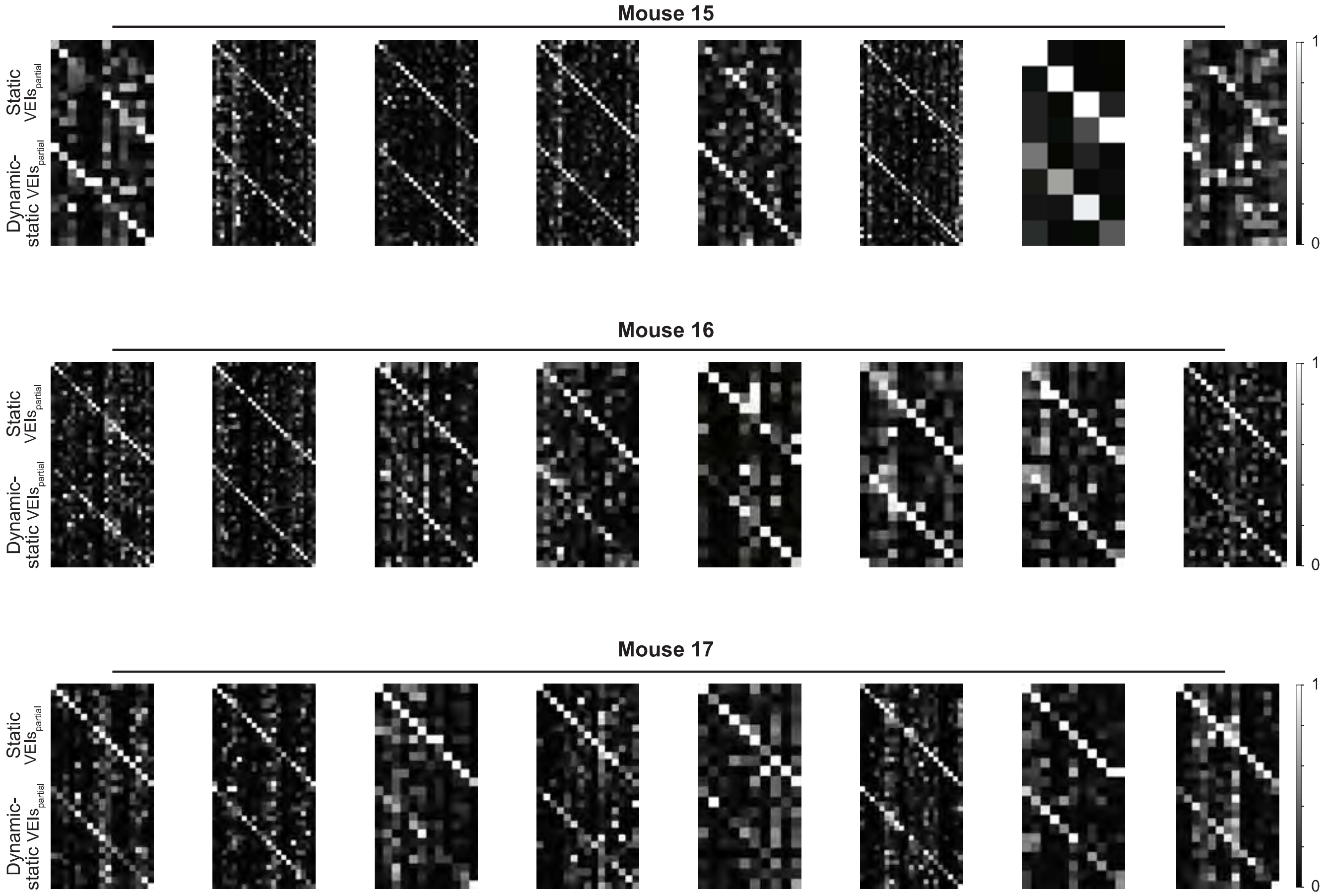

Supplemental Fig. S14. Partial-texture VEIs activated neurons with high specificity in both static and dynamic-static models. The confusion matrices showed the responses of each neuron to static VEIs$_{partial}$ (top) and dynamic-static VEIs$_{partial}$ (bottom) of all target neurons in individual scans where we presented both models' stimuli back to the mouse in closed-loop experiments. VEI$_{partial}$ responses were averaged across 20 different images with single repeat. The responses of each neuron were normalized, and each row was scaled so the maximum response across all images equaled 1. Responses of neurons to their own VEIs$_{partial}$ (along the diagonal) were larger than to other VEIs$_{partial}$ (two-sided permutation test, $P < 10^{-4}$ across all mice after BH correction).

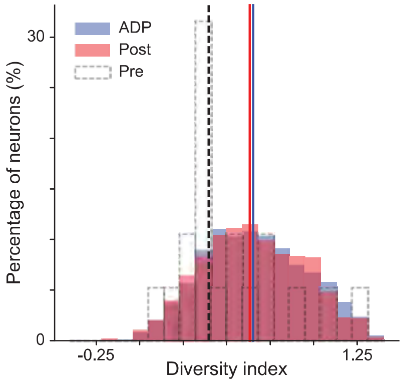

Supplemental Fig. S15. Postsynaptic neurons and ADP controls exhibited similar diversity indices. Histogram of diversity index of presynaptic, postsynaptic, and ADP controls. Diversity indices were similar between postsynaptic and ADP neurons ($P = 0.4$, two-sided Welch's t-test with 869.0 d.f.). The postsynaptic and ADP control groups were formed by pooling data across all presynaptic neurons. Data were shown from 19 presynaptic, 570 postsynaptic, and 2,486 ADP neurons, resulting in 706 connected pairs and 18,162 ADP controls.
