# Supplementary material for: Functional bipartite invariance in mouse primary visual cortex receptive fields
Source: Nat Neurosci. 2026 Feb 25;29(4):851–63. doi: 10.1038/s41593-026-02213-3 (PMC13061618; doi:10.1038/s41593-026-02213-3)
Supplement: Supplementary file 2 — Reporting Summary [file 41593_2026_2213_MOESM2_ESM.pdf]

nature portfolio

Corresponding author(s): Andreas Tolias

Last updated by author(s): Nov 21, 2025

Reporting Summary

Nature Portfolio wishes to improve the reproducibility of the work that we publish. This form provides structure for consistency and transparency in reporting. For further information on Nature Portfolio policies, see our Editorial Policies and the Editorial Policy Checklist.

Statistics

For all statistical analyses, confirm that the following items are present in the figure legend, table legend, main text, or Methods section.

n/a	Confirmed	
☐	☒	The exact sample size (n) for each experimental group/condition, given as a discrete number and unit of measurement
☐	☒	A statement on whether measurements were taken from distinct samples or whether the same sample was measured repeatedly
☐	☒	The statistical test(s) used AND whether they are one- or two-sided<br>*Only common tests should be described solely by name; describe more complex techniques in the Methods section.*
☒	☐	A description of all covariates tested
☒	☐	A description of any assumptions or corrections, such as tests of normality and adjustment for multiple comparisons
☐	☒	A full description of the statistical parameters including central tendency (e.g. means) or other basic estimates (e.g. regression coefficient) AND variation (e.g. standard deviation) or associated estimates of uncertainty (e.g. confidence intervals)
☐	☒	For null hypothesis testing, the test statistic (e.g. F, t, r) with confidence intervals, effect sizes, degrees of freedom and P value noted<br>*Give P values as exact values whenever suitable.*
☒	☐	For Bayesian analysis, information on the choice of priors and Markov chain Monte Carlo settings
☒	☐	For hierarchical and complex designs, identification of the appropriate level for tests and full reporting of outcomes
☐	☒	Estimates of effect sizes (e.g. Cohen's d, Pearson's r), indicating how they were calculated

Our web collection on statistics for biologists contains articles on many of the points above.

Software and code

Policy information about availability of computer code

Data collection	Data collection was performed using custom software developed using the following tools: ScanImage (2017a), CAIMAN (1.0), PsychToolBox 3, and Labview (2016).
Data analysis	Experiments and analysis are carried out with custom built data pipelines (https://github.com/cajal/pipeline) and custom stimulus optimization pipeline (https://github.com/cajal/featurevis). The data pipeline is developed in Matlab (2016a, 2018b) and Python (3.6, 3.8) with the following tools: DataJoint (0.12.9), MySQL (5.7.37), and CAVE (4.12,4.14,4.16) were used for storing and managing data. Numpy (1.22.2), pandas (1.5.3), SciPy (1.8.0), statsmodels (0.13.5), scikit-learn (1.0.2), and PyTorch (1.7.0+cu110) were used for model training and statistical analysis. Matplotlib (3.2.2), seaborn (0.11.2) were used for graphical visualization. Jupyter (4.4.0), Docker (19.09.13), and Kubernetes (1.19.4) were used for code development and deployment.

For manuscripts utilizing custom algorithms or software that are central to the research but not yet described in published literature, software must be made available to editors and reviewers. We strongly encourage code deposition in a community repository (e.g. GitHub). See the Nature Portfolio guidelines for submitting code & software for further information.

Data

Policy information about availability of data

All manuscripts must include a data availability statement. This statement should provide the following information, where applicable:

- Accession codes, unique identifiers, or web links for publicly available datasets
- A description of any restrictions on data availability
- For clinical datasets or third party data, please ensure that the statement adheres to our policy

The public image dataset Caltech–UCSD Birds-200-2011 (CUB-200-2011) used in this study is available at https://www.vision.caltech.edu/datasets/cub_200_2011. All other data supporting the findings of this work have been deposited on GIN (G-Node) and are publicly available at https://gin.g-node.org/cajal/microns-dei-2025 (DOI: 10.12751/g-node.9m698z).

Human research participants

Policy information about studies involving human research participants and Sex and Gender in Research.

Reporting on sex and gender	No human participant was involved in the study.
Population characteristics	No human participant was involved in the study.
Recruitment	No human participant was involved in the study.
Ethics oversight	No human participant was involved in the study.

Note that full information on the approval of the study protocol must also be provided in the manuscript.

Field-specific reporting

Please select the one below that is the best fit for your research. If you are not sure, read the appropriate sections before making your selection.

☒ Life sciences ☐ Behavioural & social sciences ☐ Ecological, evolutionary & environmental sciences

For a reference copy of the document with all sections, see nature.com/documents/nr-reporting-summary-flat.pdf

Life sciences study design

All studies must disclose on these points even when the disclosure is negative.

Sample size	Given the exploratory nature of this study, no statistical methods were used to predetermine sample size. We acquired two-photon calcium imaging data from 17 mice (>33,000 neurons) for network training and downstream analyses, and Neuropixels recordings from 6 mice (>300 neurons) for complementary analyses. Sample sizes matched or exceeded those of previous studies with similar designs.
Data exclusions	Cells were excluded a priori from analysis based on the reliability of their responses to visual stimuli and model prediction performance. For in vivo verification, cells with inconsistent anatomical and functional properties across days as described in the paper were excluded a priori from analysis.
Replication	All analyses were conducted and independently reproduced in 3–10 mice, with one analysis conducted in 2 mice.
Randomization	Within each scan, stimuli from different types were presented in a pseudo-random order.
Blinding	Experimenters were blinded to group allocation during data collection. The analyses were performed without blinding, however, the same process was applied to all groups.

Reporting for specific materials, systems and methods

We require information from authors about some types of materials, experimental systems and methods used in many studies. Here, indicate whether each material, system or method listed is relevant to your study. If you are not sure if a list item applies to your research, read the appropriate section before selecting a response.

Materials & experimental systems

n/a	Involved in the study	
☒	☐	Antibodies
☒	☐	Eukaryotic cell lines
☒	☐	Palaeontology and archaeology
☐	☒	Animals and other organisms
☒	☐	Clinical data
☒	☐	Dual use research of concern

Methods

n/a	Involved in the study	
☒	☐	ChIP-seq
☒	☐	Flow cytometry
☒	☐	MRI-based neuroimaging

Animals and other research organisms

Policy information about studies involving animals; ARRIVE guidelines recommended for reporting animal research, and Sex and Gender in Research

Laboratory animals	Seventeen mice (Mus musculus: 9 male, 8 female) aged from 6 to 17 weeks, expressing GCaMP6s in excitatory neurons via Slc17a7-Cre and Ai162 transgenic lines (stock nos. 023527 and 031562, respectively; The Jackson Laboratory) were selected for two-photon calcium imaging experiments. In addition, six mice (Mus musculus: 2 male, 4 female) aged from 14 to 27 weeks were selected for electrophysiological experiments, with 2 females expressing GCaMP6s in excitatory neurons via Slc17a7-Cre and Ai162 transgenic lines (stock nos. 023527 and 031562, respectively; The Jackson Laboratory) and the rest being C57BL/6J wildtype (stock no. 000664; The Jackson Laboratory).
Wild animals	No wild animals were used in this study.
Reporting on sex	Twenty three mice (Mus musculus) used in the study included 11 male and 12 female. Analysis disaggregated for sex was not performed due to expected generalization of principles under study across genders.
Field-collected samples	No field-collected samples were used in this study.
Ethics oversight	All procedures were approved by the Institutional Animal Care and Use Committee of Baylor College of Medicine.

Note that full information on the approval of the study protocol must also be provided in the manuscript.
